# Exploring Regioisomeric Indole–Furanone Tubulin Inhibitors

**DOI:** 10.1021/acsomega.5c07360

**Published:** 2025-09-21

**Authors:** Marcella Venettozzi, Taylor E. Coburn, Blake A. Evans, Merrelle S. Grillo, Aivy N. Le, Alex M. Minayev, Ameer H. Muse, Joed G. Otchere, Keira L. Potvin, Aidan P. Staunton, Charles M. Watroba, Delaney M. Williams, Kathryn E. Cole, Patricia Mowery, Erin T. Pelkey

**Affiliations:** † Department of Chemistry, 3494 Hobart and William Smith Colleges, Geneva, New York 14456, United States; ‡ Department of Biology, Hobart and William Smith Colleges, Geneva, New York 14456, United States; § Department of Molecular Biology and Chemistry, 6013 Christopher Newport University, Newport News, Virginia 23606, United States

## Abstract

Tubulin is involved in microtubule function and affects mitosis, cell shape, migration, and the movement of organelles. Consequently, tubulin inhibitors have emerged as promising targets for cancer treatment. We previously identified a novel antitubulin motif that combines a furanone, indole, and electron-rich dimethoxyphenyl ring. The lead indole–furanone compound (**3**) demonstrated submicromolar potency on cancer cells and inhibited tubulin polymerization. To advance these findings, we synthesized a small library of analogs of **3**, analyzed their biological activities, and used molecular modeling to elucidate binding interactions in the tubulin colchicine binding site. To assess the impact on potency, we compared: (1) dimethoxy vs trimethoxy substitution of the phenyl A-ring, (2) *N*-indole substitution of the indole B-ring, and (3) regioisomers and anhydrides of the furanone C-ring. In the process of developing the synthesis of the furanone C-ring regioisomers, we identified that a modification of conditions (NaH/inert vs DBU/air) could be used to give either the corresponding furanones or maleic anhydrides. Of the 18 synthesized compounds, six are biologically active with two exhibiting submicromolar activity against HL-60 cells. Of the six active compounds, (1) three contained dimethoxyphenyl A-rings and three contained trimethoxyphenyl A-rings largely oriented toward the tubulin α-subunit, (2) the *N*-indole substitution appeared to be less impactful on activity although having the indole nitrogen pointing down into the colchicine binding site was favored, and (3) the furanone carbonyl group located *cis* to the di- or trimethoxyphenyl A-ring and pointing toward the α-subunit was favored.

## Graphical abstract

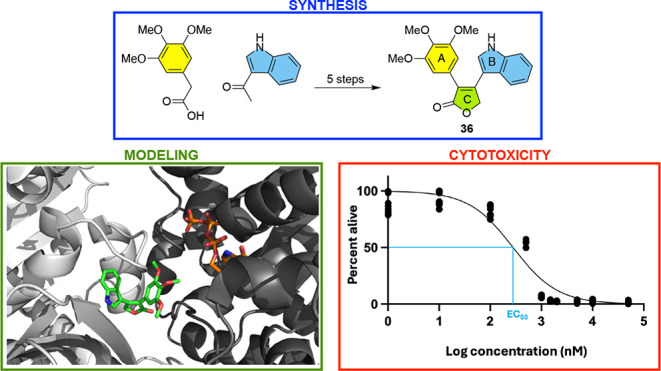

## Introduction

Microtubules are composed of tubulin α/β dimers that polymerize into protofilament chains. They are dynamic, polymerizing and depolymerizing in events called rescue and catastrophe, respectively, and are essential for mitosis, cell shape, migration, and the movement of organelles.^1^ As such, they are validated targets for drug design, particularly anticancer therapeutics. Indeed, a number of tubulin inhibitors have been FDA approved for the treatment of cancer with more in current clinical trials. However, many of these drugs target the vinca or taxane binding sites, which can exhibit multidrug resistance.^2–4^ In contrast, inhibitors that bind to the colchicine binding site (CBS) are less susceptible to common multidrug resistance mechanisms,^5,6^ and therefore, offer a promising route for improved drug development.

One important CBS microtubule-targeting agent (MTA) is the stilbenoid natural product combretastatin A-4 (CA-4, **1**), which was first isolated from *Combretum caffrum*.^7^ Compound **1** contains two different aryl groups: the A-ring containing a trimethoxyphenyl group and the B-ring containing an α-methoxyphenol group. The trimethoxyphenyl group found in **1** mirrors that of the MTA natural product colchicine (**2**)^8^ (Figure 1a, highlighted in yellow), and subsequent structure–activity relationship (SAR) studies found this moiety to be essential for antitubulin activity.^9^ Further SAR studies involving **1** have shown that the *cis* geometry of the central alkene bond is required and, subsequently, a limitation of **1** is that it is susceptible to isomerization to the inactive *trans* geometry.^10^ A significant amount of research has been aimed at identifying novel MTAs based on **1**, where the *cis* geometry of the two aryl groups are constrained by replacement of the alkene linker with a heterocyclic ring linker.^11^

**Figure 1:**
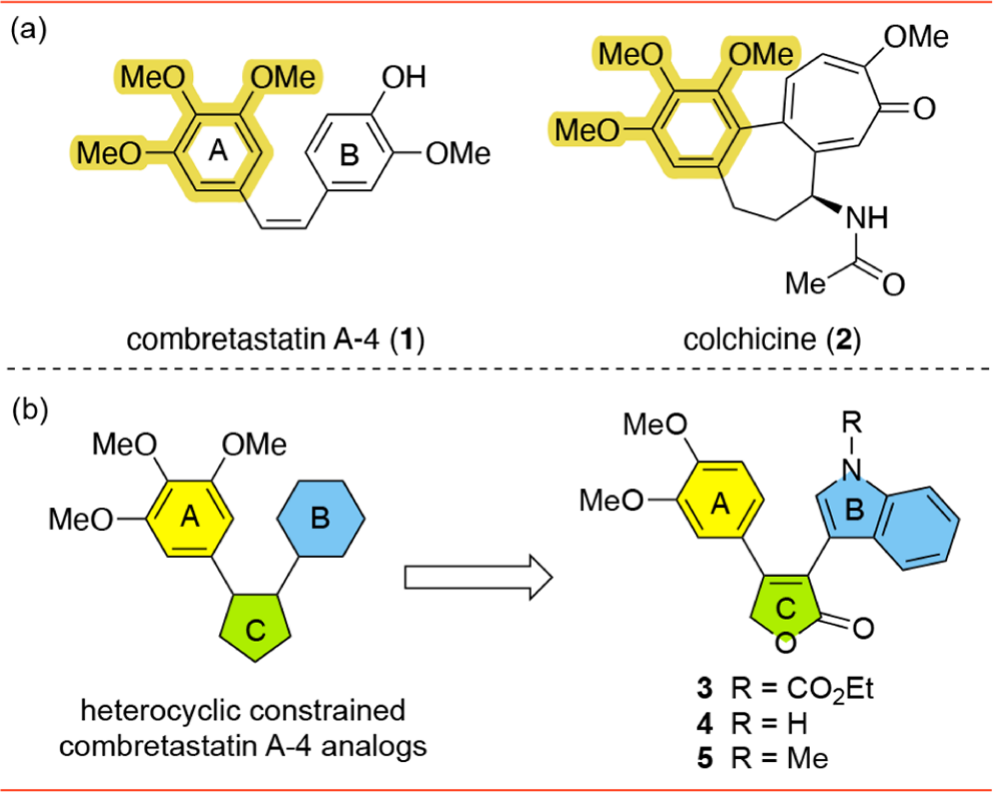
(a) Colchicine-binding site (CBS) inhibitors; (b) previously studied compounds.

We previously explored the use of pyrrolinone^12^ and furanone^13,14^ heterocyclic linkers and found that furanone **3** had submicromolar toxicity.^14^ Subsequent analysis by the National Cancer Institute (NCI-60 assay)^15^ found that **3** inhibited the majority of cell lines with a 50% growth inhibition between 10 and 100 nM, suggesting strong cytotoxicity and multicancer targeting.^14^ Profile analysis of the NCI-60 data to previously studied compounds suggested that **3** bound tubulin, which was then confirmed *in vitro*.^14^ Given the structural similarity between **3** and **1**, we hypothesize that **3** also binds in the CBS (Figure 1b).

To further expand upon the binding and potency of **3**, we synthesized a small library of analogs exploring three SAR aspects (Figure 2). First, since we had previously shown that compounds with dimethoxy groups had similar bioactivity as trimethoxy groups,^12^ we systematically tested both of these groups on the A-ring. Second, we studied the impact of different substituents (H, Me, or CO_2_Et) on the nitrogen of the indole B-ring. Finally, we investigated regioisomers of the furanone C-ring where the carbonyl group of the furanone was either located *cis* to the indole B-ring or *cis* to the A-ring, hereby referred to as Type-1 vs Type-2, respectively. We included the corresponding maleic anhydrides, thus allowing further expansion of the library and investigation of the impact of carbonyl location.^13,14^ The work described here includes synthesis of the analogs, cytotoxicity analysis, and molecular modeling to elucidate predicted binding modes that give rise to the more potent compounds.

**Figure 2:**
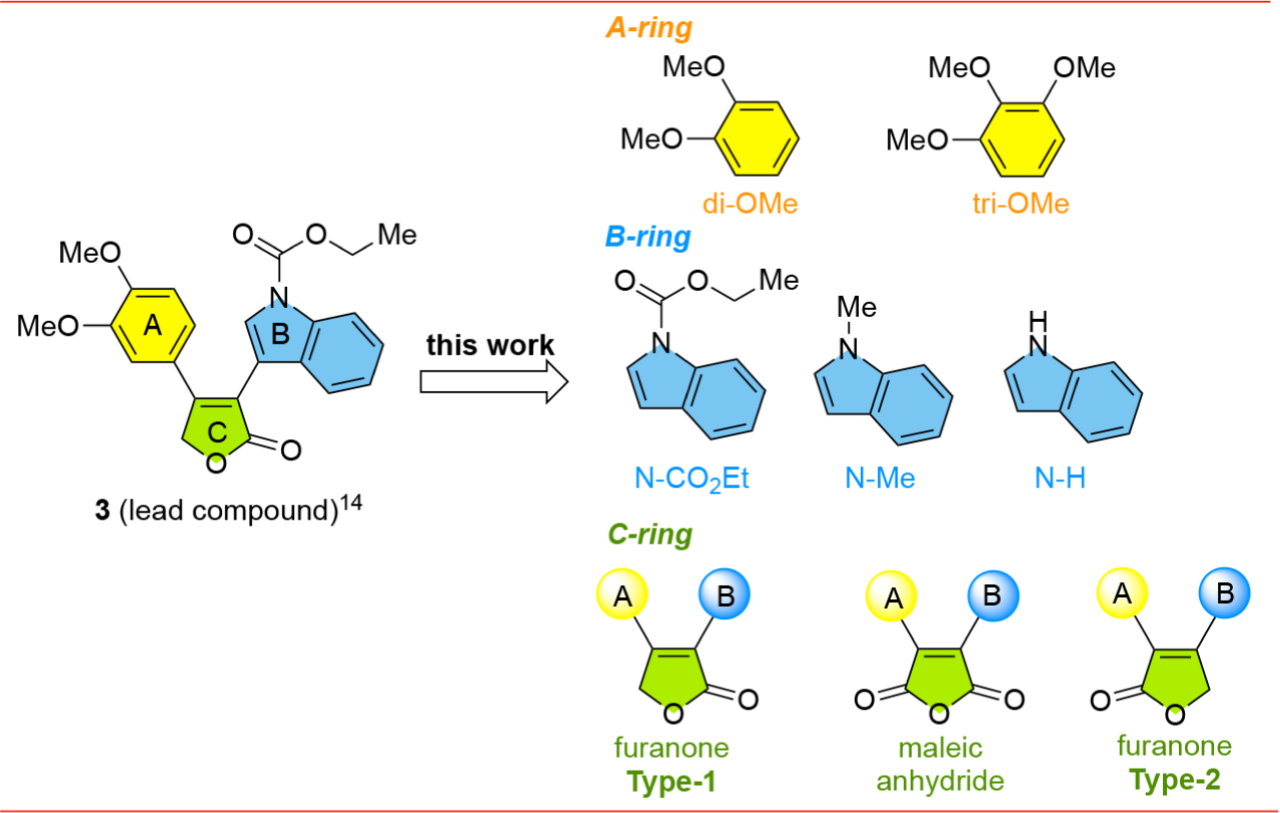
Rationally designed analogs of **3** investigated in this study.

## Results and Discussion

### Synthesis

We began the synthesis of the focused library of **3** analogs by extending our synthesis of dimethoxyphenyl A-ring analogs **3**-**5** (Figure 1b) to the corresponding Type-1 trimethoxyphenyl A-ring analogs **10**-**12** (Scheme 1), compounds that had not previously been described. Tetronic acid triflates **6**-**8** were easily accessed following our previously reported methods.^14^ Suzuki-Miyaura cross-coupling of **6** with 3,4,5-trimethoxyphenylboronic acid (**9**) gave indole–furanone **10** in 81% yield. Similarly, reactions with triflates **7** and **8** with boronic acid **9** gave the corresponding indole–furanones **11** and **12**. It is worth noting that in the case of **12**, a few experiments were run open to the air and significant amounts of 3,4,5-trimethoxyphenol were obtained as a byproduct; presumably, this compound was formed by the oxidative decomposition of the boronic acid functionality **9** under the reaction conditions. The nonproductive formation of phenols in low yields during cross-coupling reactions involving arylboronic acids have been reported by others.^16–18^ In addition to complicating the purification of **12**, the initial yields of **12** under these conditions were in the ∼20% range. Running the cross-coupling reaction under argon eliminated this nonproductive pathway and improved the yield of **12** to 57%.

**Scheme 1:**
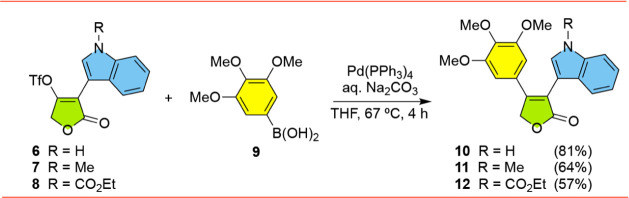
Synthesis of Type-1 Furanones **10**-**12**

We next turned our attention to the synthesis of regioisomeric Type-2 furanones of **20** and **21**, which proved to be a larger challenge than originally anticipated. Previously, we were able to access *N*-methylindole analogs **18** and **19** in good yields using a BF_3_-mediated reaction of *N*-methylindole (**16**) and the corresponding tetronic acids **13** and **14**, respectively (Scheme 2).^13^ Unfortunately, all attempts to realize this Friedel–Crafts type reaction with either indole (**15**) or known *N*-carboethoxyindole (**17**)^19^ failed to give access to the corresponding Type-2 furanones, forcing us to find a new approach.

**Scheme 2:**
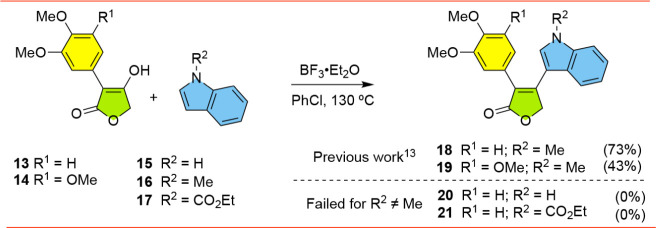
Attempted Synthesis of Type-2 Furanones **20**-**21**

We investigated the synthesis of Type-2 furanones **20** and **21**, inspired by the original Merck synthesis of rofecoxib (Vioxx), which involved the nucleophilic substitution of an α-bromoketone with an arylacetic acid followed by a DBU-mediated intermolecular aldol reaction.^20^ In the event, the requisite α-bromoketone **24** was prepared in two steps from 3-acetylindole **22** (Scheme 3). Compound **22** was converted to known *N*-carboethoxyindole **23** ^19^ in 94% yield by treatment with NaH and ethyl chloroformate. Bromination^21^ with NBS and *p*-TsOH gave α-bromoketone **24**. We then attempted to prepare the corresponding furanones by a tandem substitution/aldol sequence involving **24** and arylacetic acid **25**. The reaction of **25** with **24** under basic conditions (Et_3_N and then DBU) led to a mixture of products that included trace amounts of the desired furanone **21** and a lower polarity byproduct that was later determined to be the corresponding maleic anhydride **29**. Attempts to negate anhydride formation by running the reaction under argon, at lower temperatures (−15 °C), or with different bases (*t*-BuOK) failed to change the reaction outcome.

**Scheme 3:**
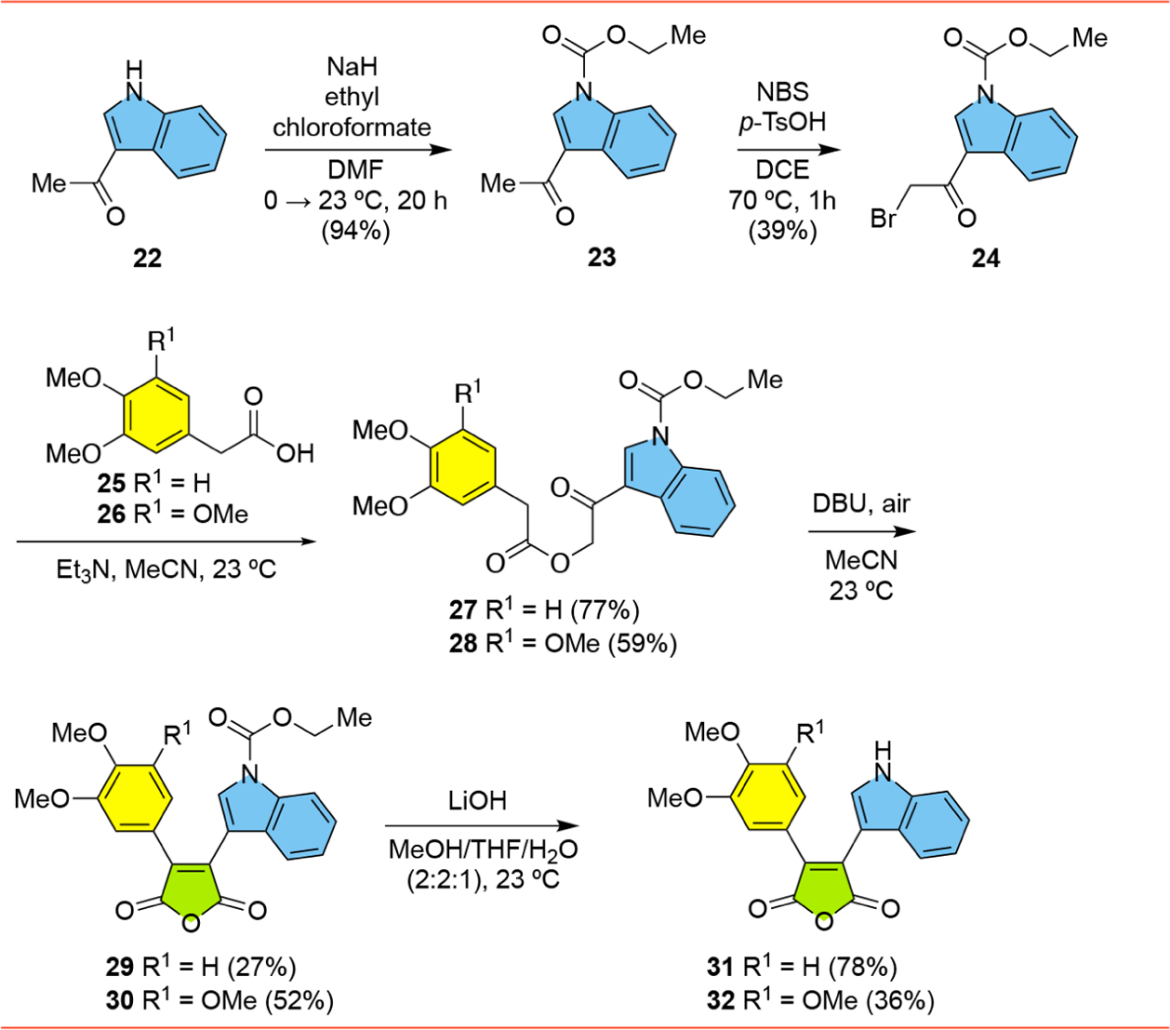
Synthesis of Indolylmaleic Anhydrides **29**-**32**

We chose to include the maleic anhydride derivatives in our library as the cytotoxicity and antitubulin activity of cyclic anhydrides remains mostly understudied, with only a few reports to date.^22,23^ Guan and coworkers disclosed the synthesis of a maleic anhydride analog of **1** with micromolar activity against three different cell lines;^24^ however, in all of these previous reports, the maleic anhydrides were compared to the corresponding maleimides. To expand on the limited SAR profile, we proposed to compare the maleic anhydrides to the corresponding furanones.

Returning to the synthesis, this type of transformation leading to anhydrides from α-acyloxyketones has been separately communicated by Pattabiraman and coworkers utilizing similar conditions to what had been used in the original rofecoxib synthesis (S_N_2 substitution in the presence of Et_3_N followed by intramolecular aldol reaction mediated by DBU).^25^ To further explore this chemistry in our system, we chose to isolate the intermediate ketoesters and explore the cyclization reactions independent of the substitution reactions. Subsequently, treatment of **24** with arylacetic acids **25** and **26** produced the corresponding ketoesters **27** and **28** in 77% and 59% yield, respectively (Scheme 3). Treatment of the latter with DBU in MeCN open to the air led to the smooth formation of maleic anhydrides **29** and **30** in modest yield. Removal of the *N*-carboethoxy groups by treatment with lithium hydroxide then gave the NH indole substrates **31** and **32** in 78% and 36% yield, respectively.

To complete our series of analogs, we prepared maleic anhydrides **33** and **34** by oxidation of the previously synthesized furanones **5** and **11**. Using the conditions previously established by Pattabiraman and coworkers,^25^ this proved to be relatively straightforward. In the event, treatment of **5** and **11** with DBU open to the air gave rise to the corresponding anhydrides **33** and **34** in 41% and 49% yields, respectively (Scheme 4). The use of O_2_ in place of air did not improve the reaction in our hands.

**Scheme 4:**
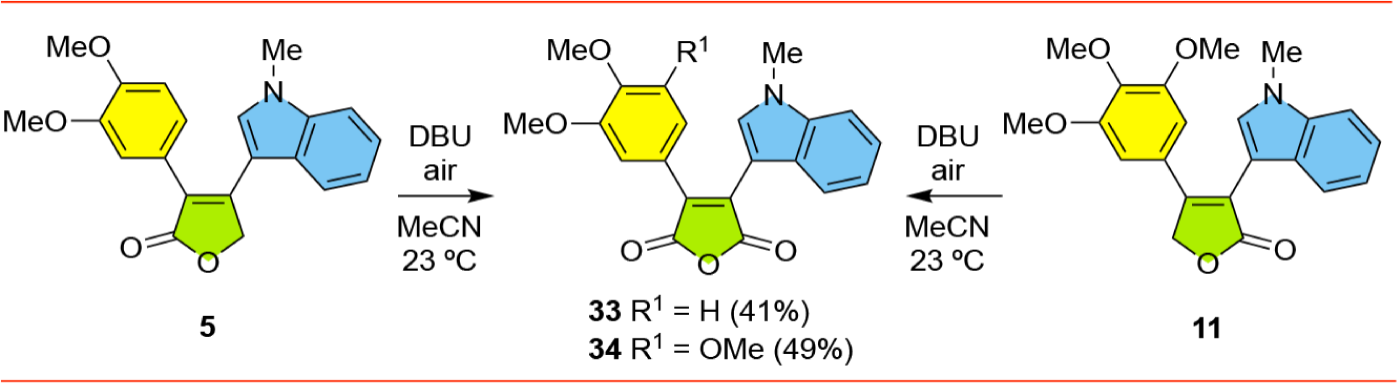
Synthesis of Indolylmaleic Anhydrides **33**, **34**

Finally, we continued investigating basic conditions that could be used to convert ketoesters **27** and **28** into Type-2 furanones (Scheme 5). A number of research groups had achieved this transformation using NaH as the base,^26–29^ and in our hands with our substrates, we were delighted that the use of NaH proved fruitful. Treatment of **27** with NaH led to the formation of both expected furanone **21** in 66% yield and also a less polar byproduct which turned out to be furanone **20** (in 22% yield). Since chromatography allowed for the separation of **20** and **21** and gave sufficient quantities of each compound to allow for subsequent cytotoxicity assays, no attempt was made to preclude the loss of the protecting group. Similarly, the reaction of the corresponding trimethoxyphenyl-containing **28** with NaH led to the formation of furanones **35** and **36**, in 36% and 19% yields.

**Scheme 5:**
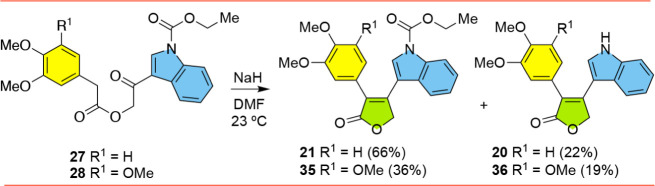
Synthesis of Type-2 Furanones **20**, **21**, **35**, **36**

### Cytotoxicity Studies

The potencies of the compounds were tested using an MTT [3-(4,5-dimethylthiazol-2-yl)-2,5-diphenyltetrazolium bromide] assay (Table 1).^30^ We previously published the EC_50_ values (EC_50_ is the effective concentration that killed 50% of the cells) of five of these analogs on U-937 lymphoma cells (**3**, **4**, **5**, **18**, and **19**).^13,14^ Herein we report the cytotoxicity activity for these five compounds plus the 13 additional compounds on HL-60 leukemia cells,^31^ a more commonly used cell line. We also tested the cytotoxicity of **1** and **2**, which were found to have low nanomolar EC_50_ values, which is consistent with published results.^32,33^

**Table 1:**
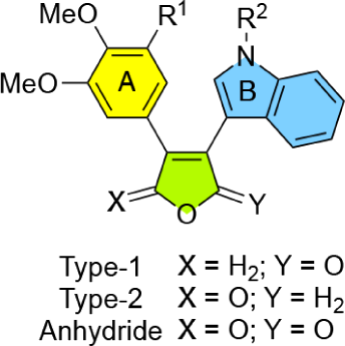
Cytotoxicity of Compounds in HL-60 Cells^a^

**Compound**	**Heterocycle**	**X**	**Y**	R^a^	R^b^	**EC**_**50**_ **+** SE^b^
**3**	Type-1 Furanone	H_2_	O	H	CO_2_Et	1.0 μM ± 0.2^a^ ^,^ ^b^ ^,^ ^d^
**21**	Type-2 Furanone	O	H_2_	H	CO_2_Et	>10 μM
**29**	Anhydride	O	O	H	CO_2_Et	Insoluble^c^
**5**	Type-1 Furanone	H_2_	O	H	Me	2.8 μM ± 0.6^c^ ^,^ ^d^
**18**	Type-2 Furanone	O	H_2_	H	Me	>10 μM
**33**	Anhydride	O	O	H	Me	>10 μM
**4**	Type-1 Furanone	H_2_	O	H	H	>10 μM
**20**	Type-2 Furanone	O	H_2_	H	H	2.4 μM ± 0.4^a^ ^,^ ^c^ ^,^ ^d^
**31**	Anhydride	O	O	H	H	>10 μM
**12**	Type-1 Furanone	H_2_	O	OMe	CO_2_Et	>10 μM
**35**	Type-2 Furanone	O	H_2_	OMe	CO_2_Et	0.8 μM ± 0.2^b^ ^,^ ^d^
**30**	Anhydride	O	O	OMe	CO_2_Et	insoluble^c^
**11**	Type-1 Furanone	H_2_	O	OMe	Me	>10 μM
**19**	Type-2 Furanone	O	H_2_	OMe	Me	1.1 μM ± 0.3^a^ ^,^ ^b^ ^,^ ^d^
**34**	Anhydride	O	O	OMe	Me	>10 μM
**10**	Type-1 Furanone	H_2_	O	OMe	H	>10 μM
**36**	Type-2 Furanone	O	H_2_	OMe	H	0.6 μM ± 0.3^b^ ^,^ ^d^
**32**	Anhydride	O	O	OMe	H	>10 μM
**CA-4** (**1**)		-	-	-	-	1.4 nM ± 0.6
**Colchicine** (**2**)		-	-	-	-	3.0 nM ± 1.0

a Cells were treated with an analog concentration range with eight replicate wells/concentration for 48 h at 37 °C before addition of MTT and solubilization. Averages and standard error from a minimum of three independent experiments were calculated.

b EC_50_ values = effective concentration that kills 50% of the cells. SE = standard error.

c Insoluble when increased to 20 mM in DMSO.

d Compounds with the same superscript letters (a–c) are statistically overlapping as measured by ANOVA, where differences are *p* < 0.05.

While **36** was the most potent compound with submicromolar EC_50_ values, analogs **3**, **19**, **35** statistically overlapped with **36** (Table 1). Therefore, the cytotoxicity patterns for these four analogs were further analyzed. Compounds **19**, **35**, and **36** form a cohesive group, as they all have trimethoxy A-rings, which aligns with literature noting the importance of trimethoxy for CBS binding.^34^ Within this group, the *N*-indole B-ring substitutions had no effect. Interestingly for **1**, the methoxy on the B-ring is known to be important, but not essential, for binding.^34^ Finally, this group had a Type-2 orientation of the furanone, suggesting the importance of carbonyl positioning for binding. Compound **3** formed its own second group, as it had a Type-1 regioisomer with a dimethoxy A-ring, where CO_2_Et substitution of the B-ring *N*-indole was important. Given the structural differences between **3** and analogs containing the trimethoxyphenyl A-ring, **3** may make different interactions in the binding pocket that compensate for the lack of a third methoxy group and varied structural parameters (see below).

The literature regarding the stability of anhydrides in a cellular environment is less clear; it is possible that they may be modified *in vivo*, but the diaryl substituents may also enhance stability. Regardless, we found that the anhydride compounds were either insoluble at appropriate concentrations for cytotoxicity studies or inactive, and so we did not further explore their stability in this study.

Given the encouraging biological results, we examined the general “druggability” of the four statistically overlapping most potent compounds.^35^ All of the molecules obey Lipinski’s Rules, suggesting that they would be reasonably absorbed, although they are only moderately soluble. They also fall slightly outside of the insaturation range, which may minimally affect bioavailability. As described above, all display synthetic feasibility. A table of representative data is provided in Table S1.

### Molecular Modeling Studies

To further understand inhibitor binding, we turned to molecular modeling. The methoxy A-ring of **36**, the most potent compound, points toward the α-tubulin subunit and makes hydrogen bonds with αN101 and αT179. The furanone C-ring hydrogen bonds with αV181, and the nitrogen of the indole B-ring is oriented down toward the CBS (Figure 3). Compound **36** also makes a number of hydrophobic interactions with both subunits (Table S2). Like **36**, the trimethoxyphenyl A-rings of **19** and **35**, which are also Type-2 compounds, are oriented toward, and hydrogen bond with, the α-subunit. Their indole rings are also pointed down, suggesting that this binding mode is favorable for activity (Figure S1). In all three inhibitors, the trimethoxyphenyl A-ring makes hydrogen bonds with αN101, hydrophobic interactions with αS178 and βA250, and hydrophobic and/or hydrogen bonds with αT179. A comparative cartoon is shown in Figure 4 and a full list of interactions is provided in Table S2.

**Figure 3:**
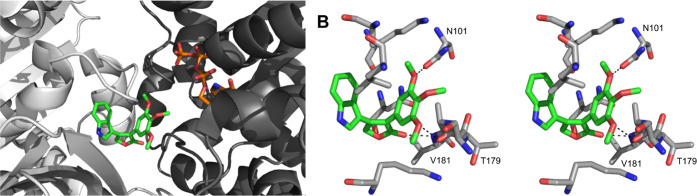
Molecular modeling of **36**. (A) The lowest predicted free energy of binding of **36** in the CBS. The α-subunit is in dark gray and the β-subunit is in light gray. (B) A stereoview of interactions between **36** and tubulin. **36** is shown in green (C = green, N = blue, O = red), GTP is in orange (C = orange, N = blue, O = red), and the side chains of the interacting residues are shown in gray (C = gray, N = blue, O = red). Hydrogen bonds in B are represented as dashed lines.

**Figure 4:**
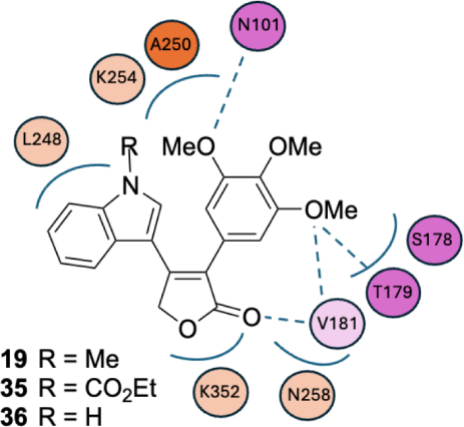
Cartoon representation of the main inhibitor-tubulin interactions of the most potent trimethoxy inhibitors (**19**, **35**, and **36**). Purple residues are from the tubulin α-subunit, and orange residues are from the tubulin β-subunit. Darker colors indicate interactions in all 3 inhibitors, while lighter colors represent interactions in 2 of 3 inhibitors. The dashed lines indicate hydrogen bonding, and the ellipses represent hydrophobic interactions.

The dimethoxy analogs, on the other hand, are less consistent in their binding. Compounds **5** and **20** bind as described above: the dimethoxyphenyl A-ring is oriented toward the α-subunit and the nitrogen of the indole B-ring is oriented down toward the CBS (Figure S1). Their furanone C-rings are opposite, however, as **5** and **20** are Type-1 and Type-2 compounds, respectively. They each make a single hydrogen bond, **5** with βM259 and **20** with αV181. Compound **3** is an anomaly being the only compound with a “flipped” orientation; the pose with the most negative predicted free energy of binding has the dimethoxyphenyl A-ring faces the β-subunit and the nitrogen of the indole B-ring is oriented up (Figure S1). A full list of interactions is provided in Table S2.

While both **3** and **19** were previously shown to bind tubulin *in vitro*,^13,14^ this is the first structural analysis of **3**, and it is not surprising that a dimethoxy compound might bind differently in the pocket. However, **3** still made similar overall interactions; the dimethoxyphenyl A-ring makes hydrophobic interactions with βA250 and the inhibitor hydrogen bonds to αN101 through the indole B-ring (rather than the methoxy A-ring); the indole B-ring also hydrogen bonds with αA180. Taken together, with the exception of **3**, the modeling results suggest that activity is largely governed by (1) the methoxy A-ring orienting toward the α-subunit, (2) the nitrogen of the indole B-ring pointing down, and (3) the furanone being a Type-2 regioisomer. Furthermore, the relative predicted free energies of binding are similar (−9.4 ± 0.4 kcal/mol), which is consistent with the observed comparable activity. Lastly, overall interactions are dominated by hydrophobic interactions, rather than hydrogen bonding, which can be used in the design and synthesis of future compounds.

## Conclusions

We set out to systematically examine the biological activity of a set of 18 indole–furanone analogs based on lead compound **3**. The analogs were categorized into two groups based on the location of the indole substituent: 3-indolylfuranones (Type-1 furanones) and 4-indolylfuranones (Type-2 furanones). The synthesis of the full range of Type-2 furanones involved an aldol condensation approach that proved challenging. Ultimately, the careful selection of the reaction conditions could be used to prepare either the desired Type-2 furanones or the synthesis of the corresponding indolylmaleic anhydrides. The complete set of analogs allowed us to probe the impact of the following structural features on cytotoxicity: (1) substitution on the methoxy A-ring, (2) *N*-substitution of the indole B-ring, and (3) regioisomers and anhydrides of the furanone C-ring. We found that analogs with submicromolar potency had trimethoxy-substituted methoxy A-rings with Type-2 orientation, and that substitution on the indole B-ring had no impact. Molecular modeling showed that these compounds made similar binding interactions in the CBS pocket. In addition, we report that an equally cytotoxicity compound, **3**, was a Type-1 furanone with a dimethoxy-substituted A-ring and CO_2_Et substitution of the *N*-indole B-ring, but may be effective due to making overlapping contacts as the trimethoxy potent group.

## Supplementary Material



## Notes

Experimental procedures, spectral data, biological data, molecular modeling figures, and copies of ^1^H and ^13^C NMR spectra (PDF)
