# Supplementary material for: Exploring Regioisomeric Indole–Furanone Tubulin Inhibitors
Source: ACS Omega. 2025 Sep 21;10(38):44675–82. doi: 10.1021/acsomega.5c07360 (PMC12489669; doi:10.1021/acsomega.5c07360)

Supporting Information

Exploring Regioisomeric Indole-Furanone Tubulin Inhibitors

Marcella Venettozzi,[b] Taylor E. Coburn,[a] Blake A. Evans[a] Merrelle S. Grillo,[a] Aivy N. Le,[c] Alex M. Minayev,[c] Ameer H. Muse,[a] Joed G. Otchere,[c] Keira L. Potvin,[a] Aidan P. Staunton,[b] Charles M. Watroba,[b] Delaney Williams,[b] Kathryn E. Cole,[c,]* Patricia Mowery,[b,]* and Erin Pelkey[a,]*

[a]*Department of Chemistry, Hobart and William Smith Colleges, Geneva, NY, 14456*

[b]*Department of Biology, Hobart and William Smith Colleges, Geneva, NY, 14456*

[c]*Department of Molecular Biology and Chemistry, Christopher Newport University, Newport News, VA, 23606*

**Corresponding authors: pelkey@hws.edu, mowery@hws.edu, kathryn.cole@cnu.edu*

Table of Contents

Modeling SI-2

Biological Assays SI-8

Synthesis SI-27

NMR Spectra SI-45

Modeling.

Molecular Modeling Studies. Docking studies were performed using AutoDock Vina.[†] Compounds were first energy-minimized and converted to .pdb files using ChemBioDraw Ultra 14.0 and Chem3D Pro 14.0 (CambridgeSoft). Chains A and B of a tubulin-colchicine complex (PBD 4O2B, less the ions, solvent, and inhibitor) were used as the receptor; GTP was included in the model. The search space was defined around the CBS as following: center_x = 17.056, center_y = 66.401, and center_z = 42.197, with size_x = 40, size_y = 40, size_z = 40, with the spacing set to 0.375 Å and the exhaustiveness set to 50 iterations. Colchicine was modeled as a control; the modeling results are overlaid with its X-ray crystal structure below (**Figure S2**).

Model Analysis. The .pdbqt output files were visualized in Pymol. Models were assessed based on binding in the CBS, along with the predicted free energies of binding; the most negative predicted free energies of binding are compared in the main text. Tubulin-inhibitor interactions were measured using Coot,[‡] and SwissADME was used to predict the general drug characteristics of the molecules.[*]

[†]*J. Comput. Chem.* **2010***, 31*, 455

[‡]*Acta Crystallogr. Sect. D: Biol. Crystallogr*. **2004***, 60,* 2126-2132.

[*]*Sci. Rep.* **2017**, *7*, 42717

Table S1. Representative ADME data of 4 statistically overlapping most potent compounds suggesting overall favorable druggable characteristics.

Compound	MW (g/mol)	Consensus Log P	ESOL Log S	Synthetic Accessibility
**3**	407	3.59	-4.61	3.55
**19**	379	3.24	-4.16	3.28
**35**	437	3.58	-4.69	3.74
**36**	365	3.22	-4.12	3.17

*All have 0 Lipinski violations. Applicable ranges/values are as follows: molecular weight < 500 g/mol; consensus Log P < 5; ESOL Log S: log S scale: insoluble < -10 < poorly < -6 < moderately < -4 < soluble < -2 <very < 0 < highly; and synthetic accessibility: 1 (easy) , SA score < 10 (difficult)

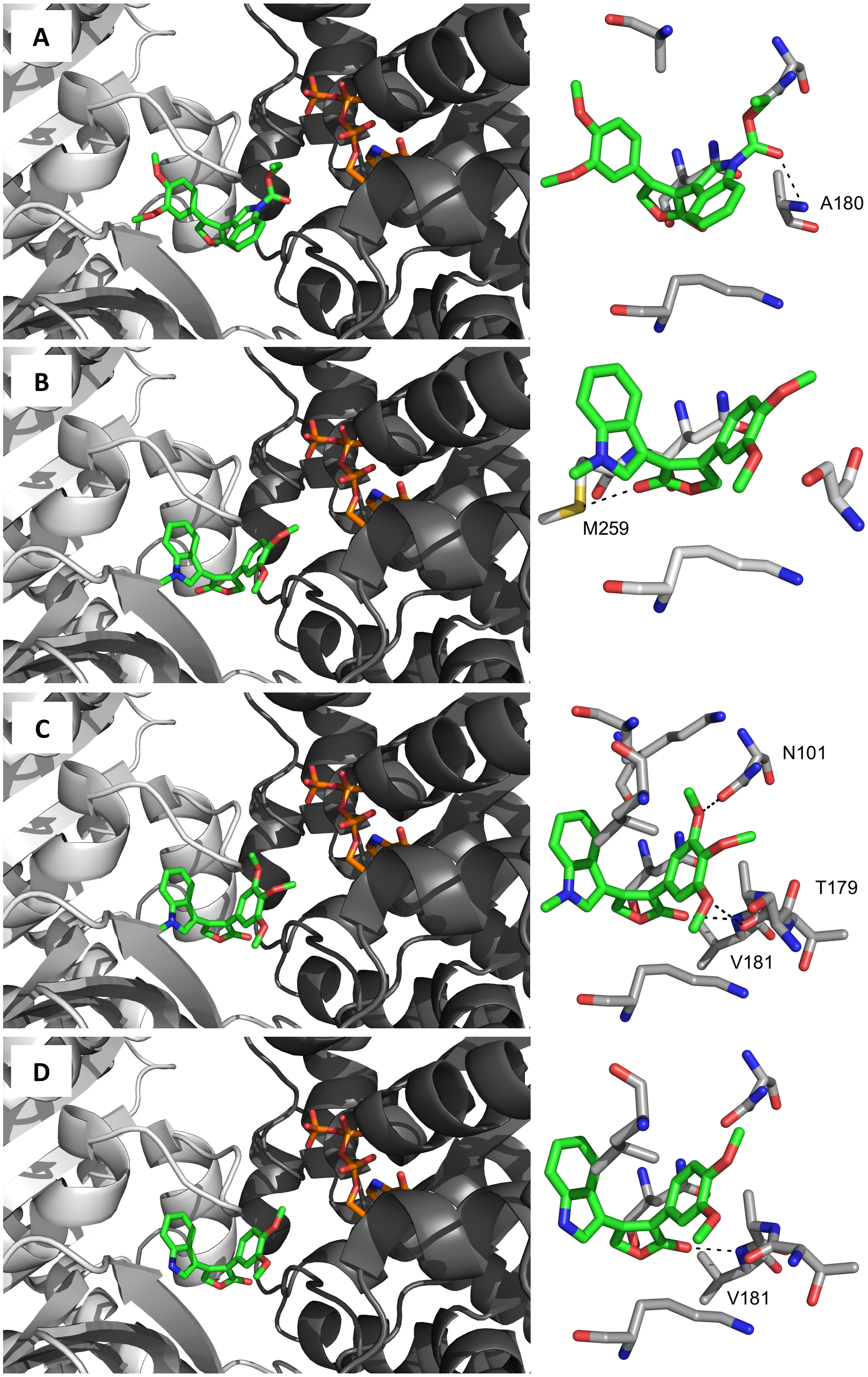
A
A180
B
M259
C
N101
T179
V181
D
V181

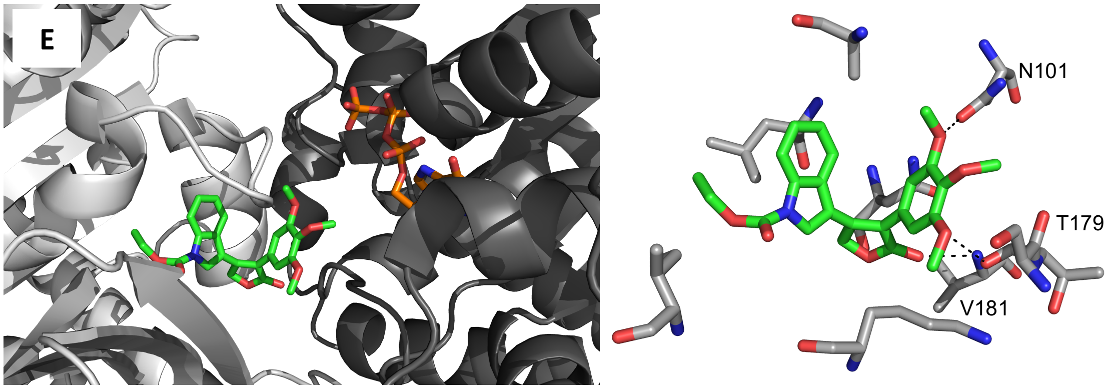

Figure S1. Molecular modeling of compounds (A) **3** (B) **5** (C) **19** (D) **20** and (E) **35** in the CBS. The molecules are shown in green (C = green, N = blue, O = red), GTP is in orange (C = orange, N = blue, O = red), and the sidechains of the interacting residues are shown in gray- the alpha subunit is dark gray and the beta subunit is light gray (C = gray, N = blue, O = red). Hydrogen bonds are represented as dashed lines.

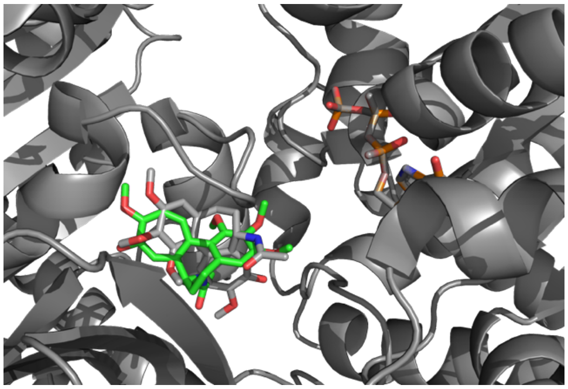

Figure S2. Overlay of colchicine (**2**) modeling with its crystal structure (PDB: 4O2B). **2** is shown as green sticks (C = green, N = blue, O = red); colchicine from the crystal structure is in gray sticks (C = gray, O = red); GTP is in orange (C = orange, N = blue, O = red); and the alpha subunit is dark gray and the beta subunit is light gray.

Table S2. Tubulin-inhibitor interactions.

3	Ligand	Interaction	Distance (Å)	Tubulin Chain	Residue
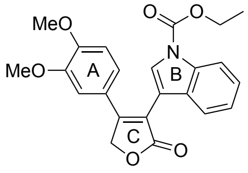	ring A	hydrophobic		β	A250
	ring B	hydrogen	3.12	α	N101
	ring B	hydrophobic		α	A180
	ring B	hydrogen	3.26	α	A180
	ring C	hydrophobic		β	N258
	ring C	hydrophobic		β	K352

5	Ligand	Interaction	Distance (Å)	Tubulin Chain	Residue
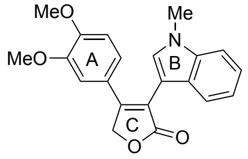	ring A	hydrophobic		α	S178
	ring C	hydrophobic		β	N258
	ring C	hydrogen	3.40	β	M259
	ring C	hydrophobic		β	K352

19	Ligand	Interaction	Distance (Å)	Tubulin Chain	Residue
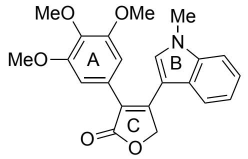	ring A	hydrogen	2.97	α	N101
	ring A	hydrophobic		α	S178
	ring A	hydrophobic		α	T179
	ring A	hydrogen	3.43	α	T179
	ring A	hydrophobic		α	A180
	ring A	hydrogen	3.19	α	V181
	ring A	hydrophobic		β	A250
	ring A	hydrophobic		β	K254
	ring A	hydrophobic		β	N258
	ring A	hydrophobic		β	K352
	ring B	hydrophobic		β	L248

20	Ligand	Interaction	Distance (Å)	Tubulin Chain	Residue
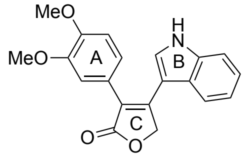	ring A	hydrophobic		α	N101
	ring A	hydrophobic		α	T179
	ring A	hydrophobic		β	N258
	ring B	hydrophobic		β	L248
	ring C	hydrophobic		α	A180
	ring C	hydrogen	3.12	α	V181
	ring C	hydrophobic		β	N258
	ring C	hydrophobic		β	K352

35	Ligand	Interaction	Distance (Å)	Tubulin Chain	Residue
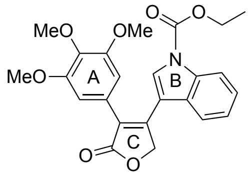	ring A	hydrogen	2.91	α	N101
	ring A	hydrophobic		α	S178
	ring A	hydrophobic		α	T179
	ring A	hydrogen	3.37	α	T179
	ring A	hydrophobic		β	A250
	ring B	hydrophobic		β	A250
	ring B	hydrophobic		β	L255
	ring B	hydrophobic		β	I318
	ring B	hydrophobic		β	K352
	ring C	hydrophobic		α	N101
	ring C	hydrogen	3.21	α	V181
	ring C	hydrophobic		β	K352
	ring C	hydrophobic		β	N258

36	Ligand	Interaction	Distance (Å)	Tubulin Chain	Residue
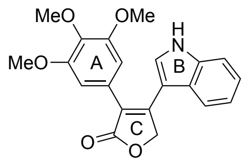	ring A	hydrogen	2.98	α	N101
	ring A	hydrophobic		α	S178
	ring A	hydrophobic		α	T179
	ring A	hydrogen	3.36	α	T179
	ring A	hydrophobic		β	A250
	ring A	hydrophobic		β	K254
	ring B	hydrophobic		β	L248
	ring C	hydrophobic		α	A180
	ring C	hydrogen	3.14	α	V181
	ring C	hydrophobic		β	N258
	ring C	hydrophobic		β	K352

Biological Assays.

Cells and media. HL-60 cells (American Type Culture Collection, Manassas, VA) were grown in IMDM with high glucose, L-glutamine, and HEPES (ATCC), supplemented with penicillin/streptomycin (Sigma Aldrich, St. Louis, MO), and 20% fetal bovine serum (Gibco Thermo Fisher, Waltham, MA). Cells were grown at 37 °C in 5% CO_2.

MTT Cytotoxicity Assay [3-(4,5-dimethylthiazol-2-yl)-2,5-diphenyltetrazolium bromide]. Assay was performed as previously described.[†] Cells were diluted to $1x10^5$ cells/mL and 95 μL were plated/well in a 96 well plate (NEST Biotech, Wuxi, China). "Background absorbance" wells contained 100 μL of media and "no analog treatment absorbance" wells contained 95 μL of cells with 5uL of dimethyl sulfoxide (DMSO) (Fisher Scientific, Waltham, MA) in PBS (phosphate buffered saline) to the highest concentration of analog sample. Analogs were dissolved in DMSO at 20 mM, diluted to appropriate concentrations in PBS, and 5 μL of analog solutions were added to the plate with eight replicates per treatment. Cells were incubated for 48 hr at 37 °C, then 10μL of 5 mg/mL MTT (Sigma Aldrich) in PBS was added, and cells were incubated for 4 hr at 37 °C. Cells were treated with 100 μL solubilization solution [10% Triton-X 100 in acidic isopropanol (0.1N HCl)] and the absorbance was measured at 570 nM on a Multiskan Plus plate reader (Waltham, MA). Cell viability curve was calculated as: (avg. analog treated absorbance – avg. background absorbance) / (avg. no analog treatment absorbance – avg. background absorbance). Cell viability was normalized and plotted against the log of the analog concentration.

Relative EC_{50} calculations were determined on GraphPad Prism 10 (Boston, MA). Each compound was tested in a minimum of three independent trials. Where compounds induce full cell death, values were normalized and EC_{50} numbers calculated. A representative curve is in the abstract. This EC_{50} curve is compound **36** tested with the following concentrations: 1 nM, 10 nM, 100 nM, 500 nM, 1 μM, 1.5 μM, 2 μM, 5 μM, 10 μM, 50 μM. Statistics were calculated with GraphPad Prism 10 using one-way ANOVA.

[†]*J. Immunolog. Methods.* **1983**, *65*, 55

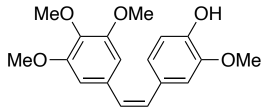

combretastatin A-4 (**1**)

Figure S3a.

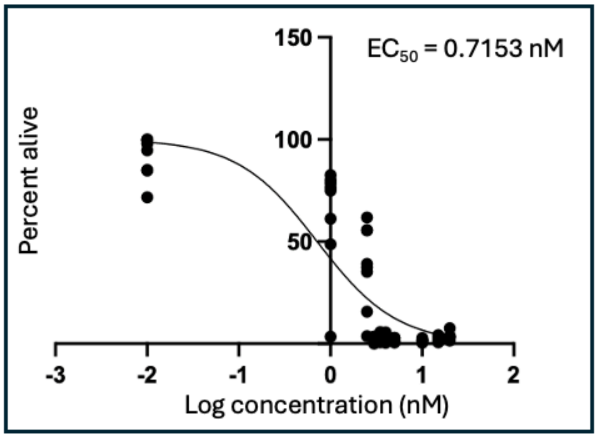

Figure S3d.

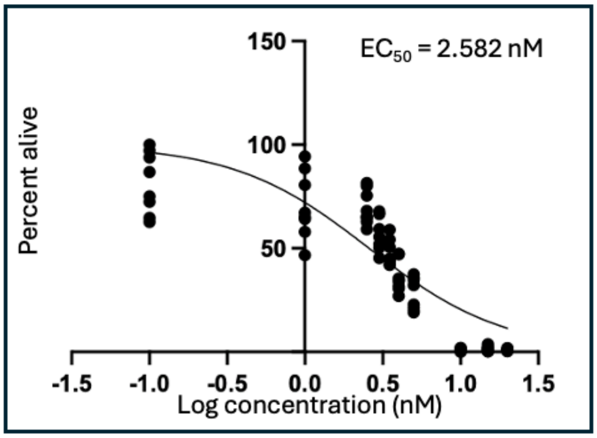

Figure S3b.

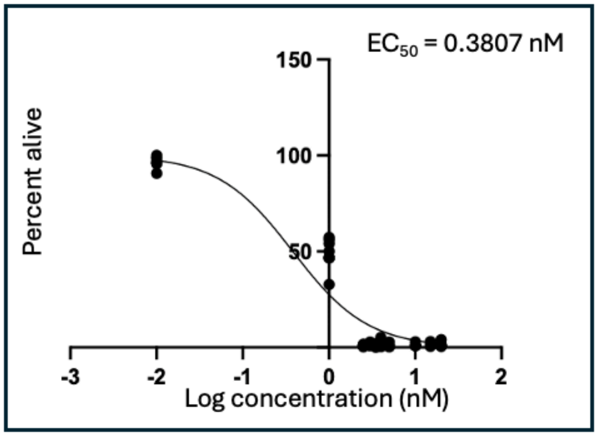

Figure S3e.

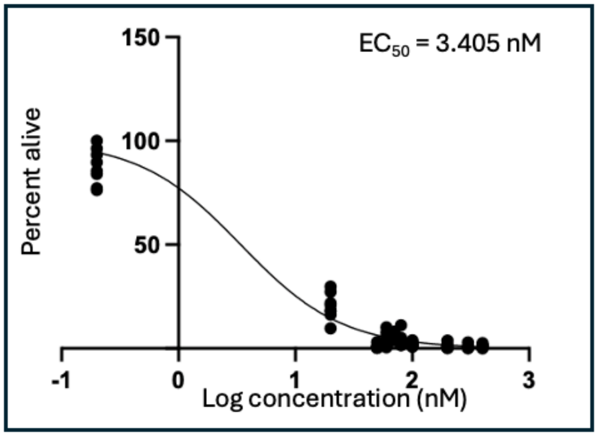

Figure S3c.

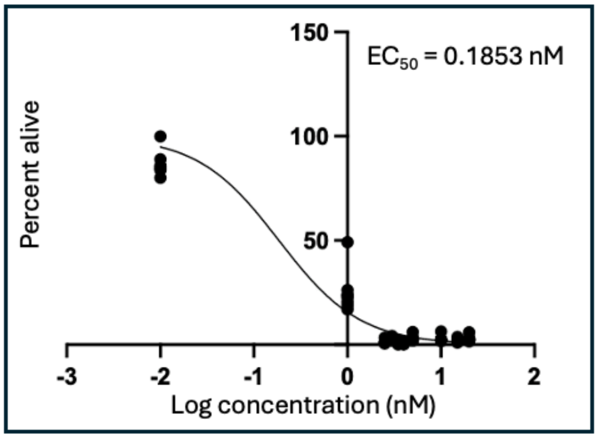

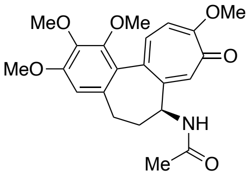

colchicine (**2**)

Figure S4a.

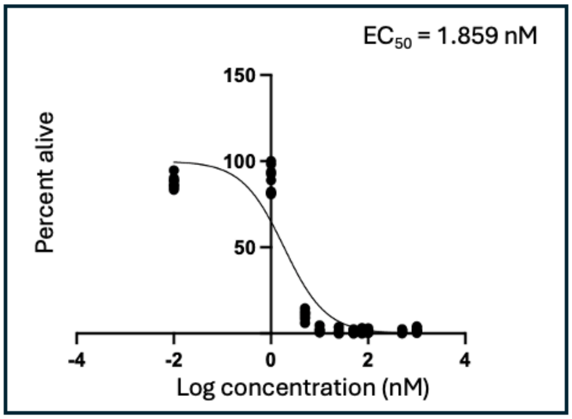

Figure S4d.

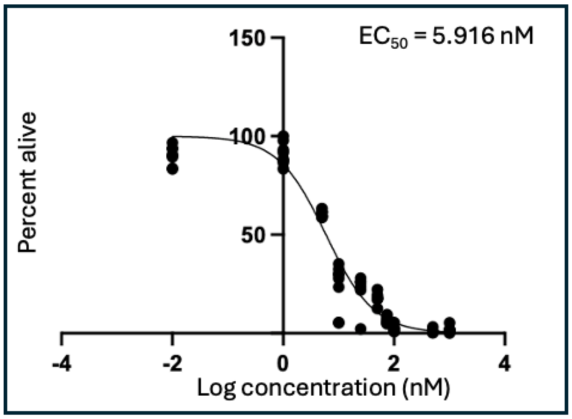

Figure S4b.

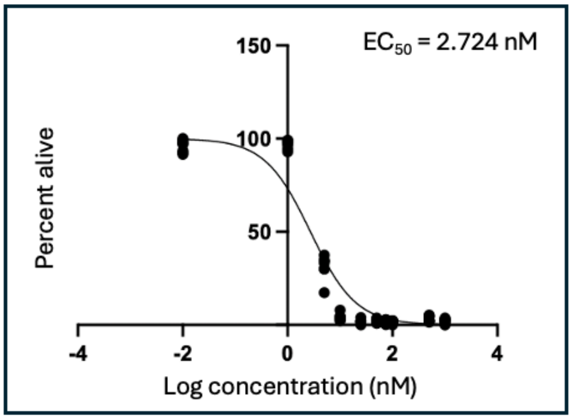

Figure S4c.

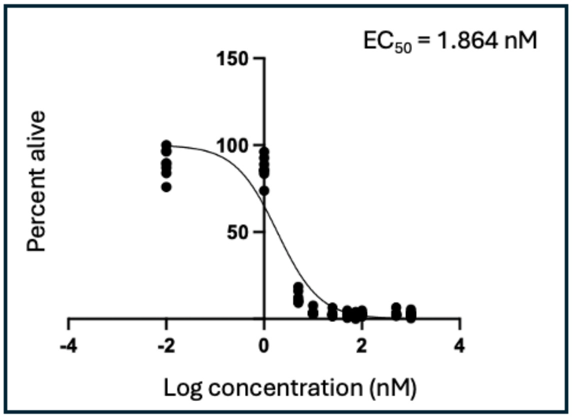

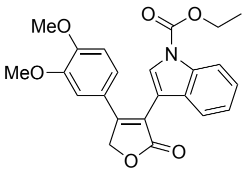

3

Figure S5a.

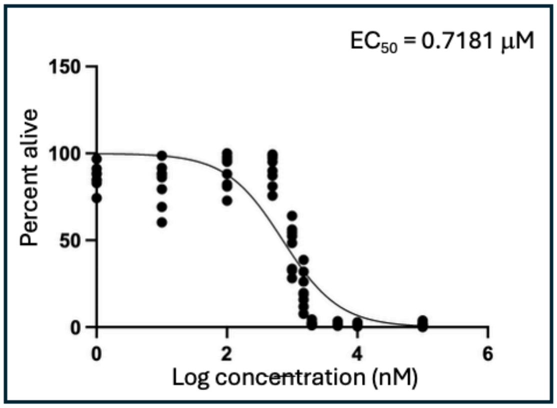

Figure S5d.

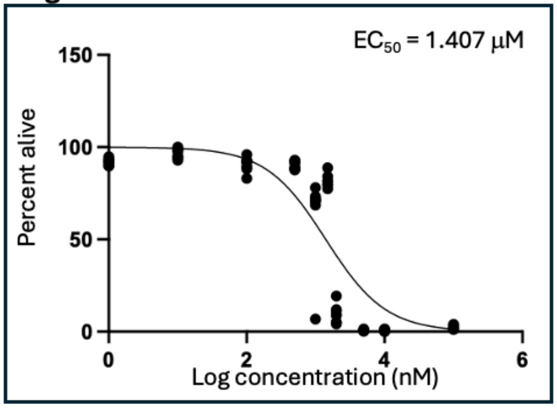

Figure S5b.

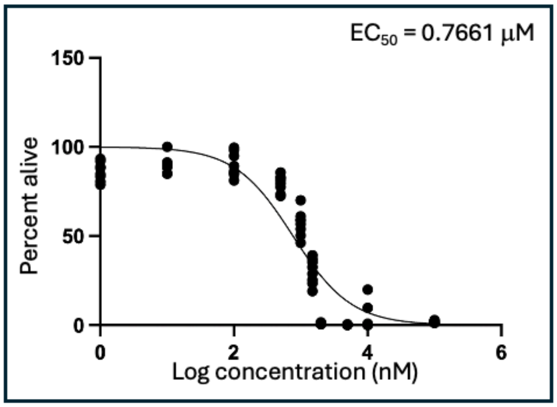

Figure S5c.

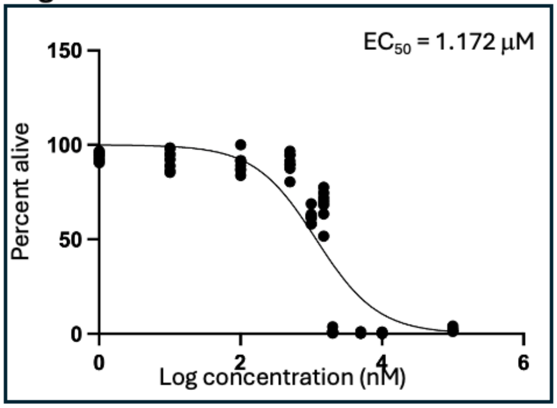

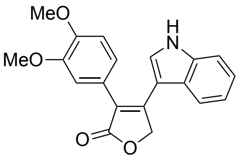

4

Figure S6a.

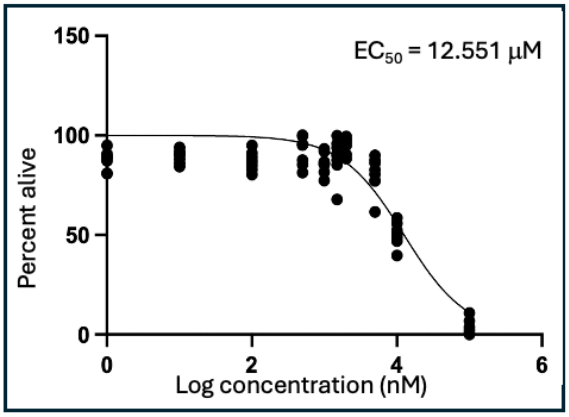

Figure S6b.

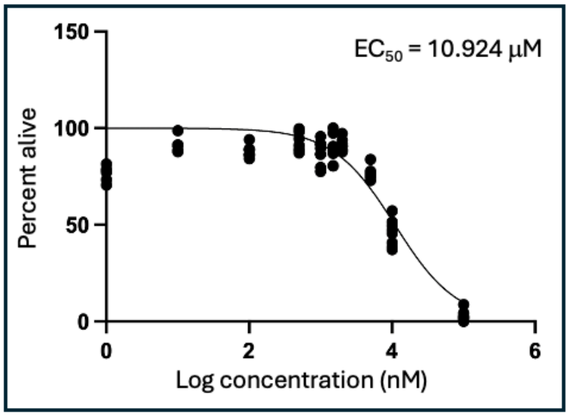

Figure S6c.

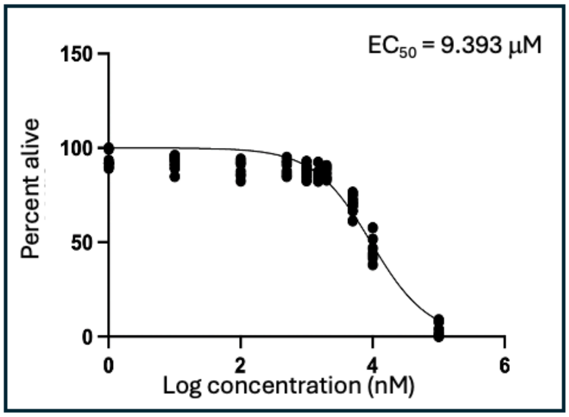

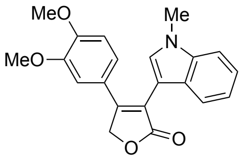

5

Figure S7a.

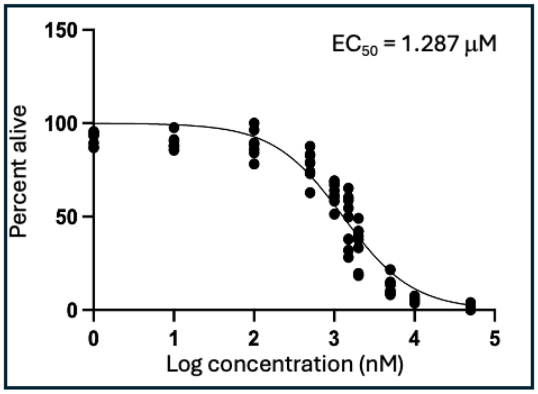

Figure S7d.

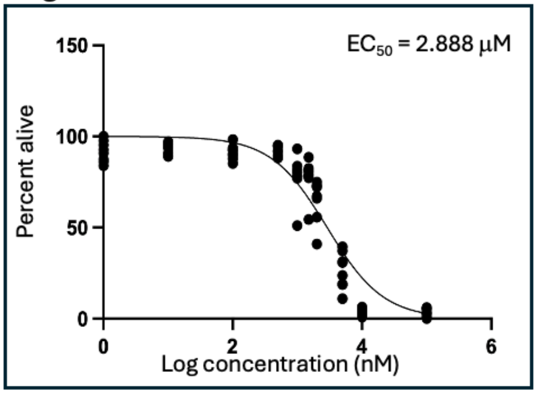

Figure S7b.

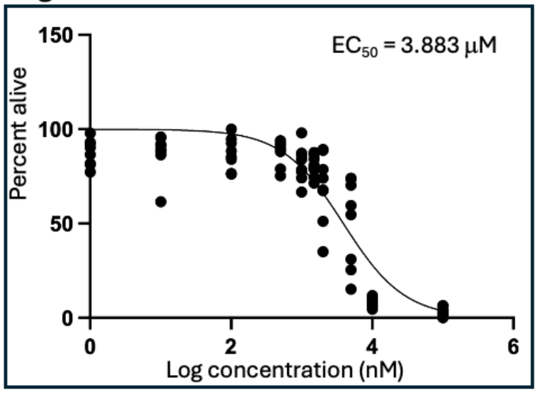

Figure S7c.

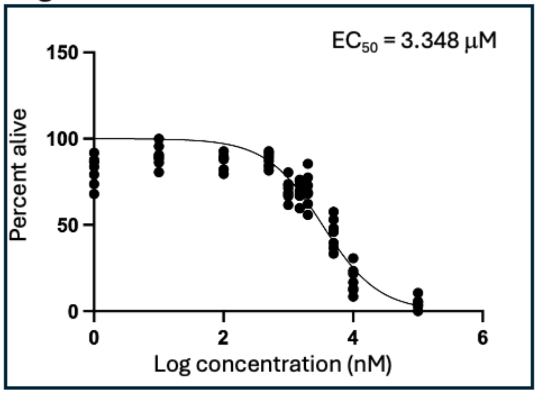

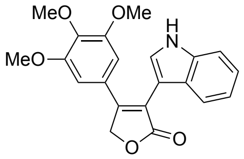

10

10

Figure S8a.

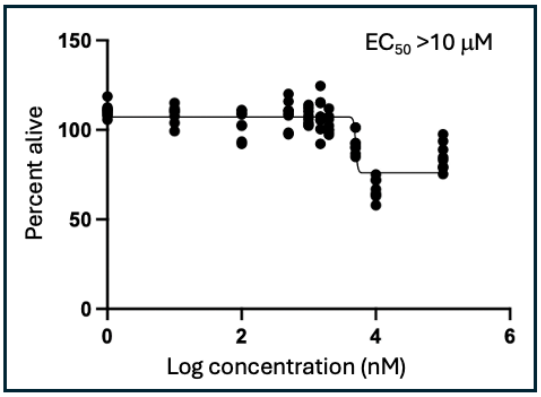

Figure S8d.

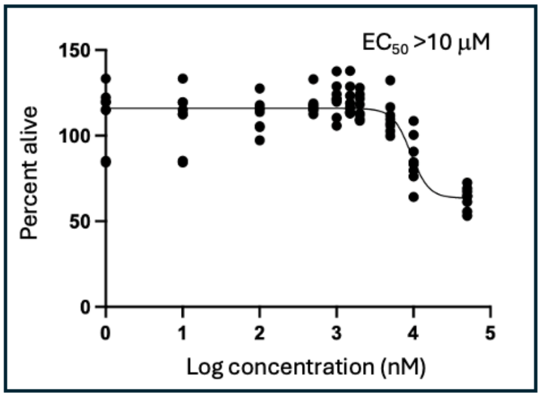

Figure S8b.

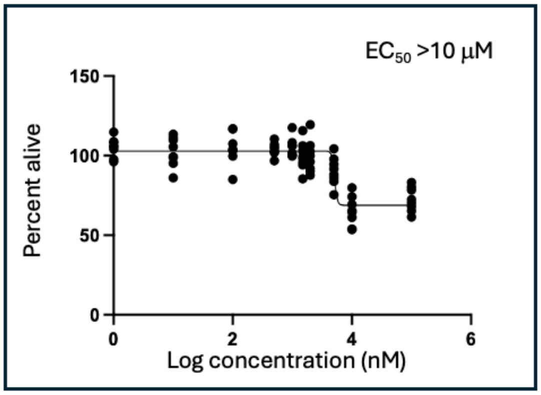

Figure S8c.

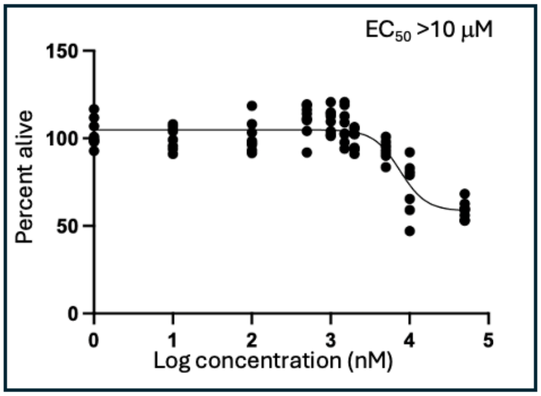

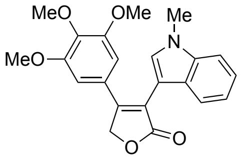

11

Figure S9a.

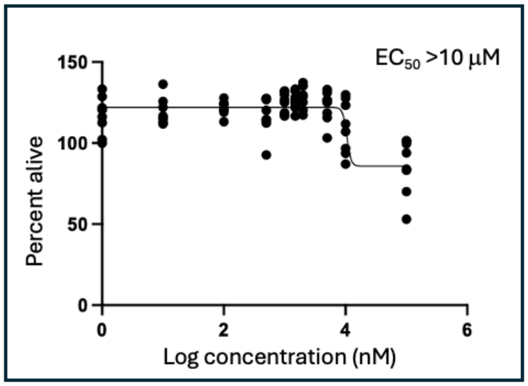

Figure S9b.

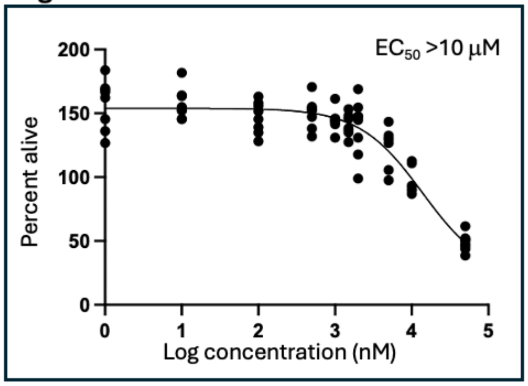

Figure S9c.

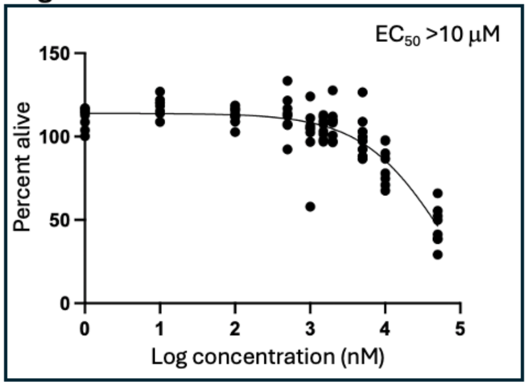

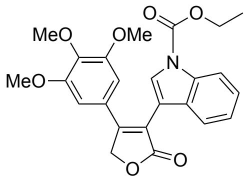

12

Figure S10a.

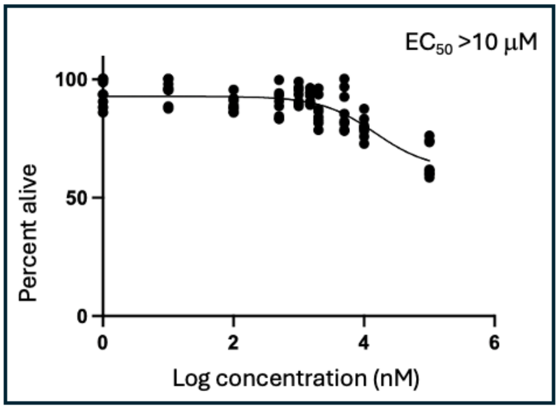

Figure S10d.

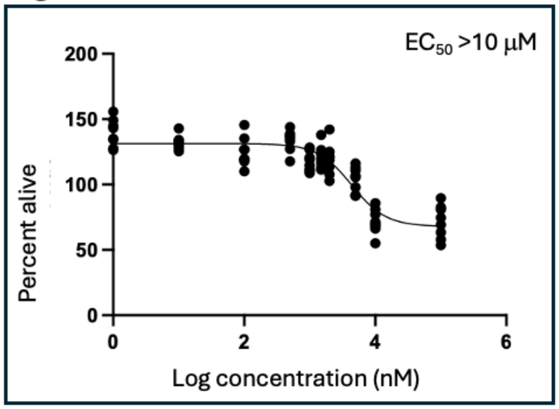

Figure S10b.

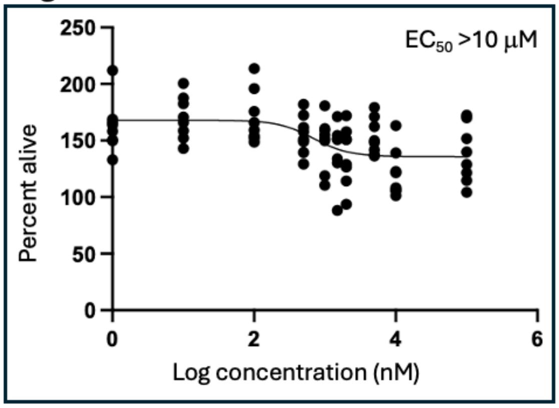

Figure S10e.

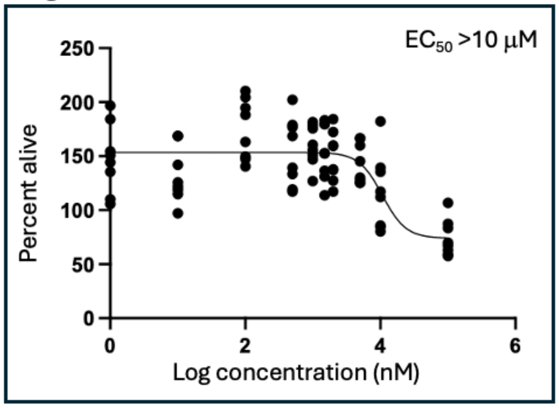

Figure S10c.

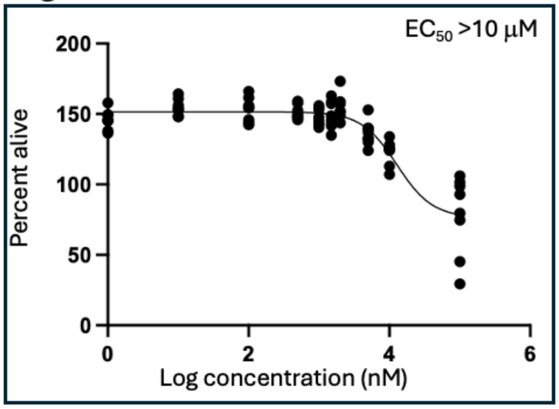

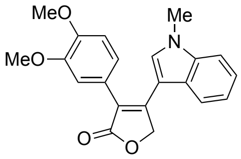

18

Figure S11a.

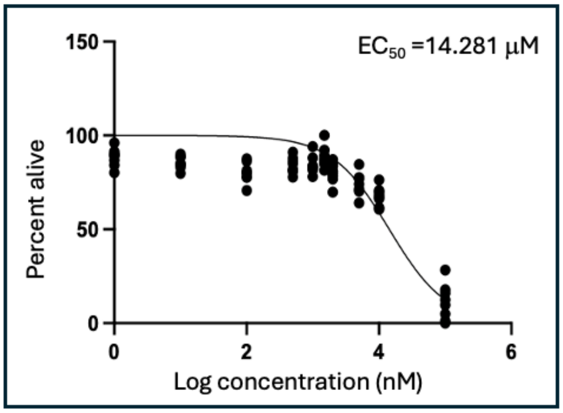

Figure S11b.

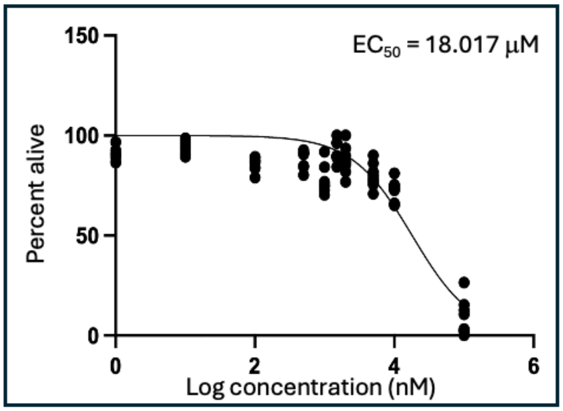

Figure S11c.

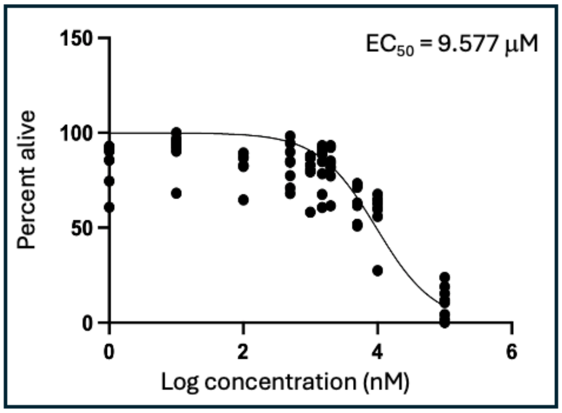

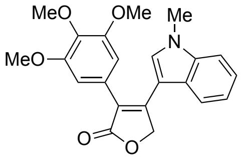

19

Figure S12a.

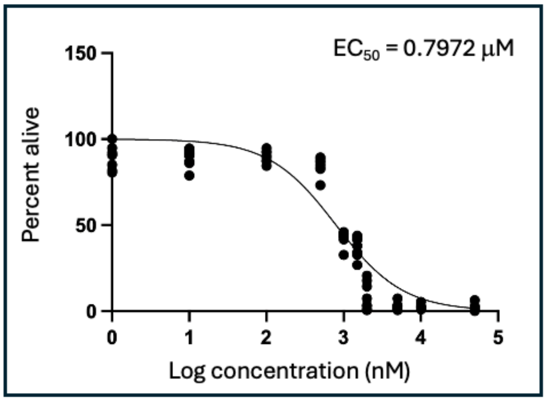

Figure S12c.

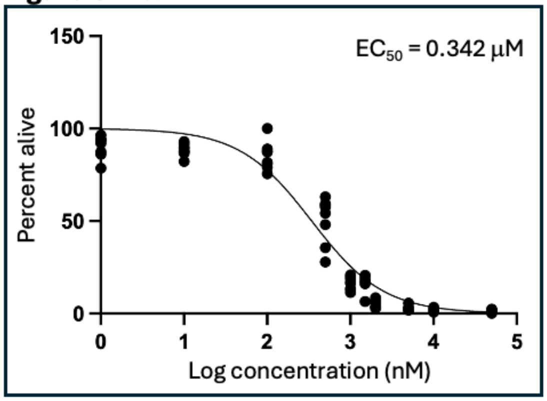

Figure S12b.

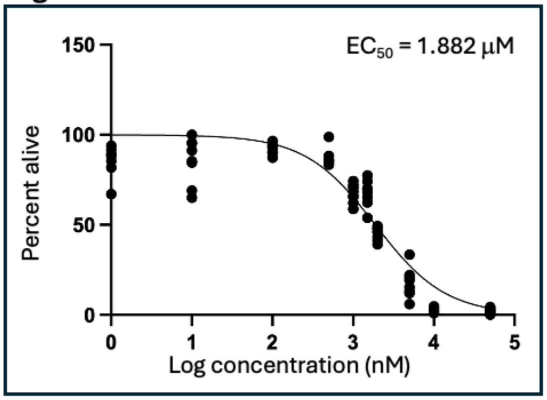

Figure S12e.

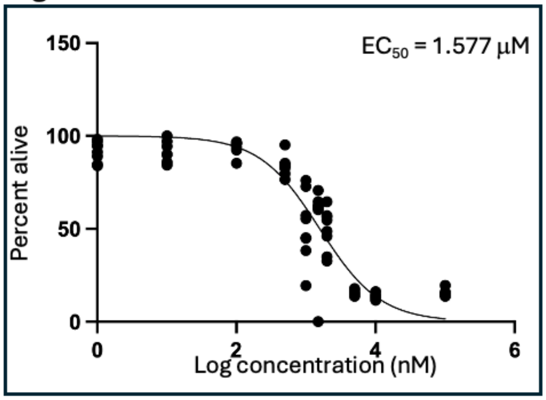

Figure S12c.

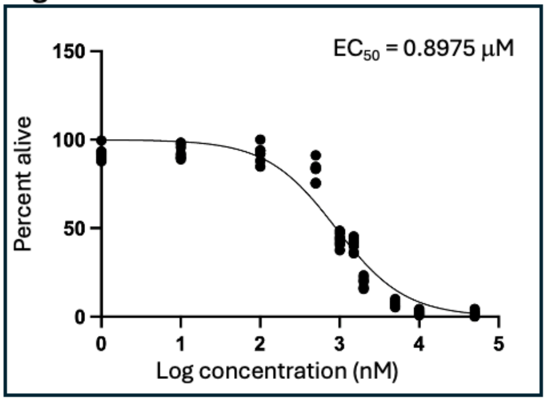

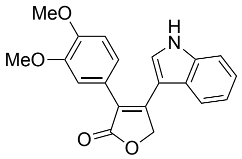

20

Figure S13a.

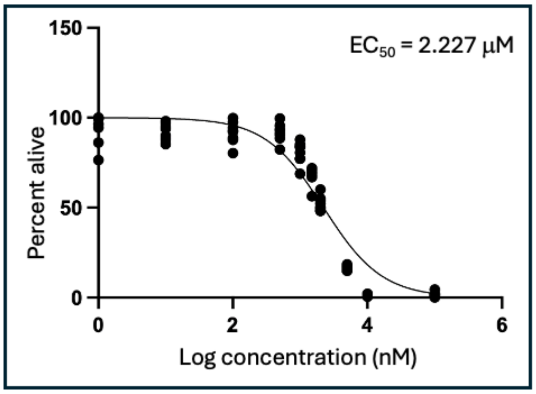

Figure S13d.

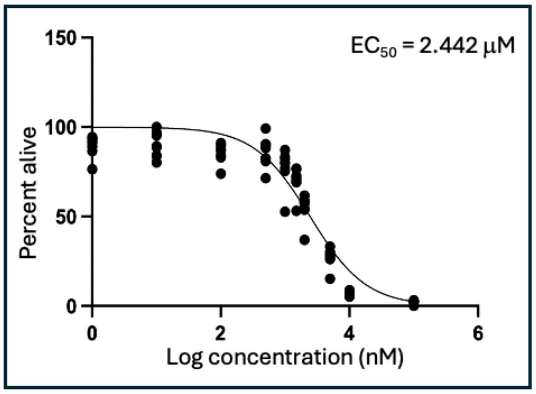

Figure S13b.

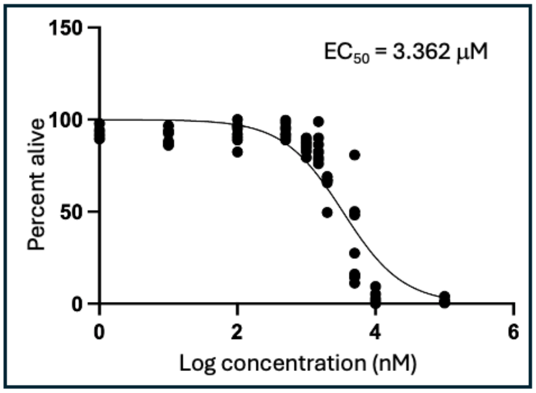

Figure S13c.

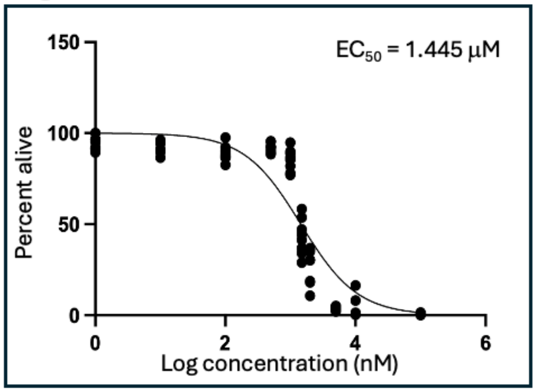

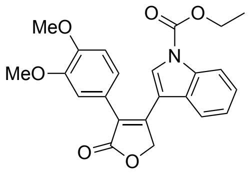

21

Figure S14a.

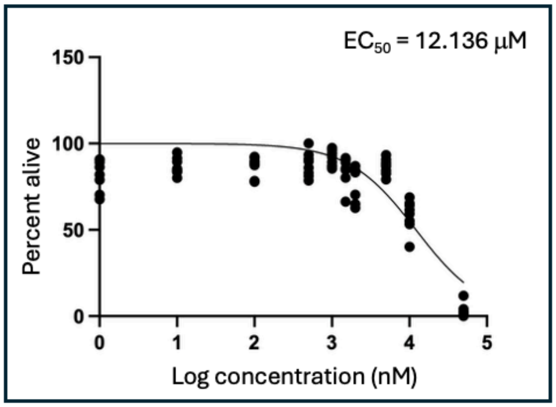

Figure S14b.

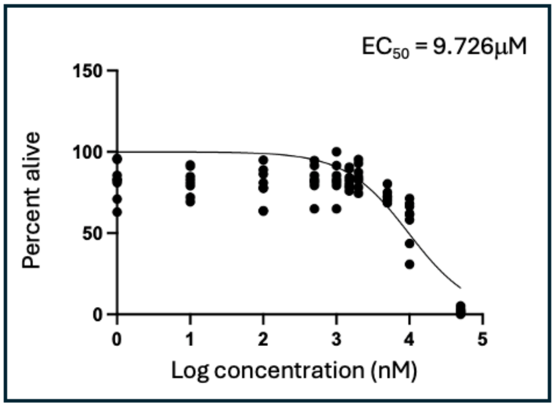

Figure S14c.

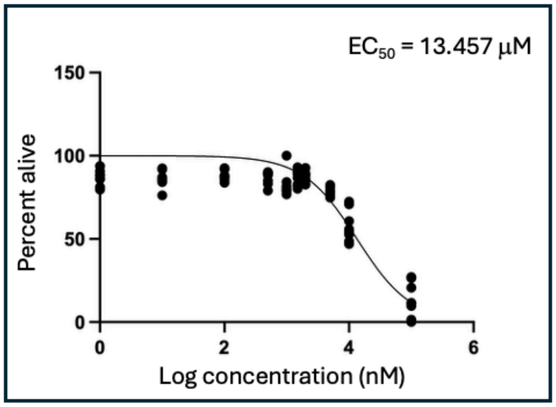

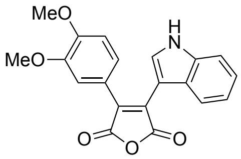

31

Figure S15a.

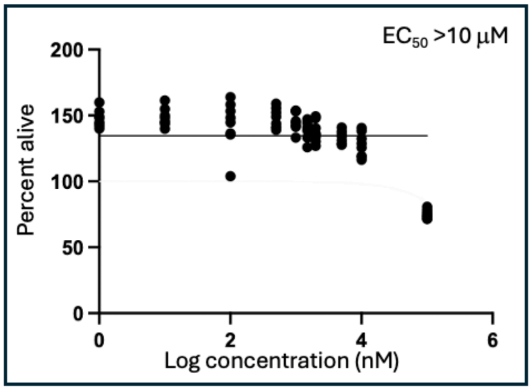

Figure S15b.

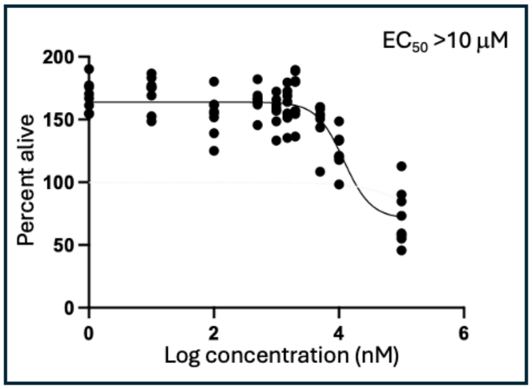

Figure S15c.

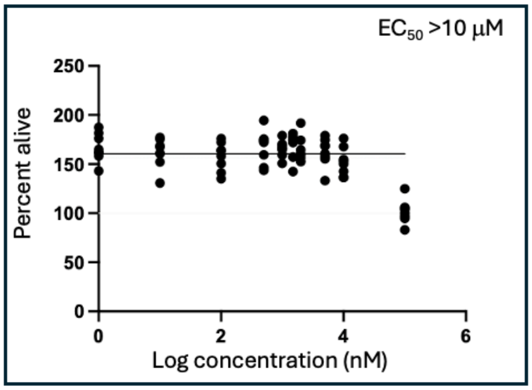

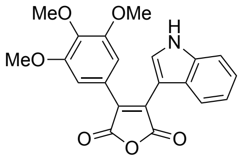

32

Figure S16a.

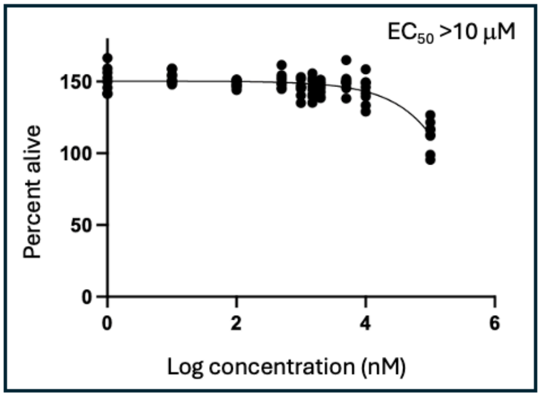

Figure S16b.

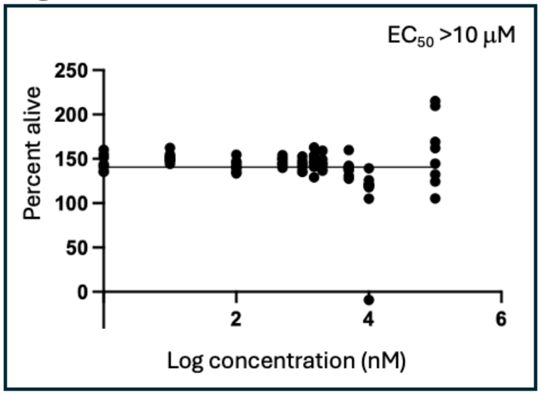

Figure S16c.

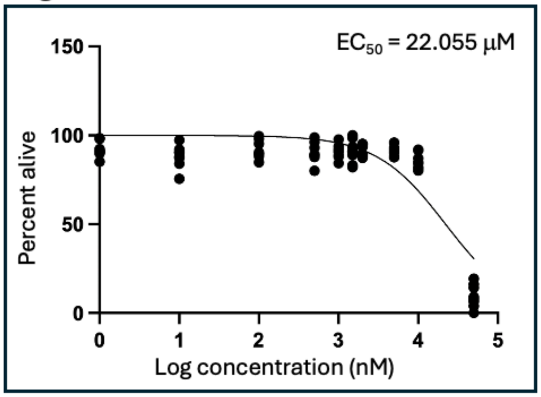

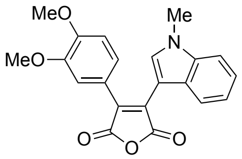

33

Figure S17a.

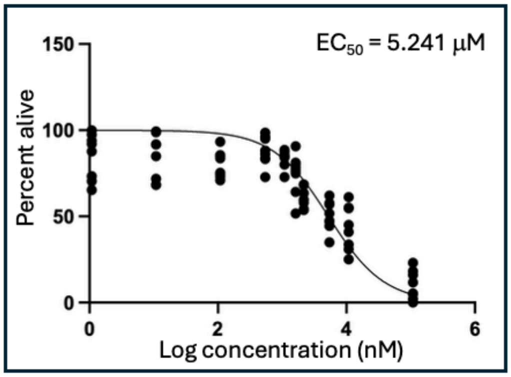

Figure S17b.

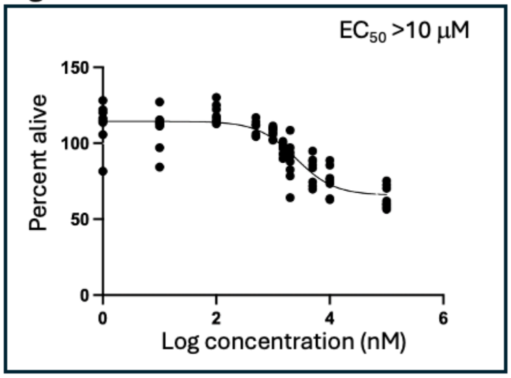

Figure S17c.

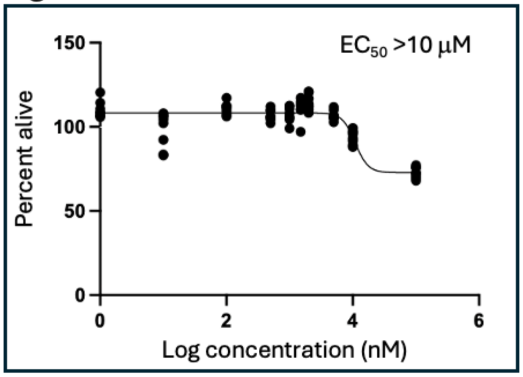

Figure S17d.

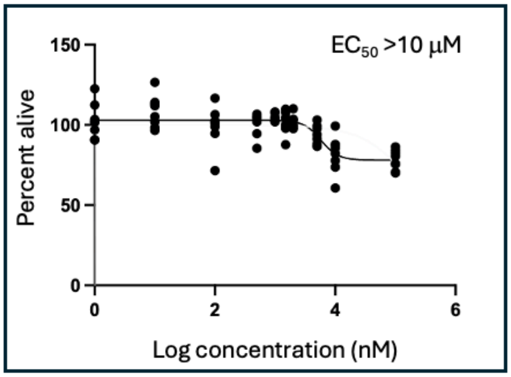

Figure S17e.

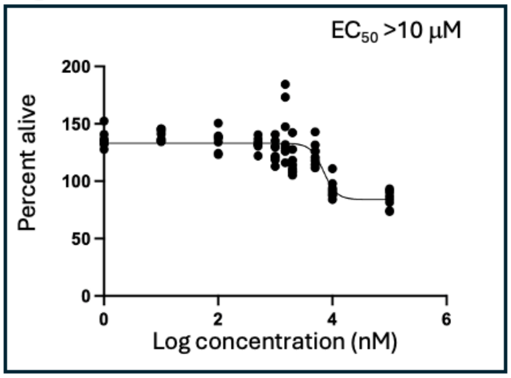

Figure S17f.

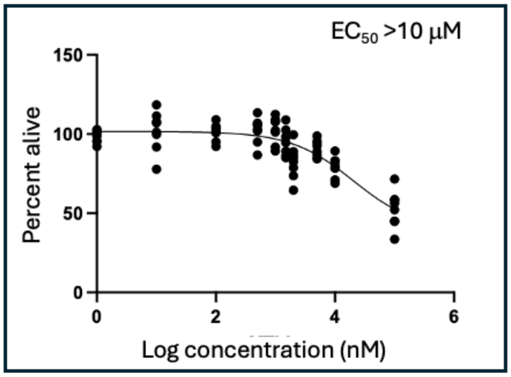

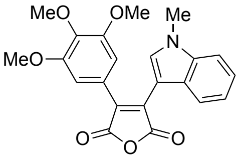

34

Figure S18a.

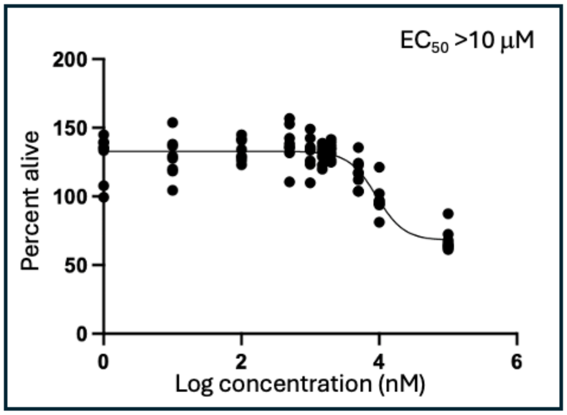

Figure S18b.

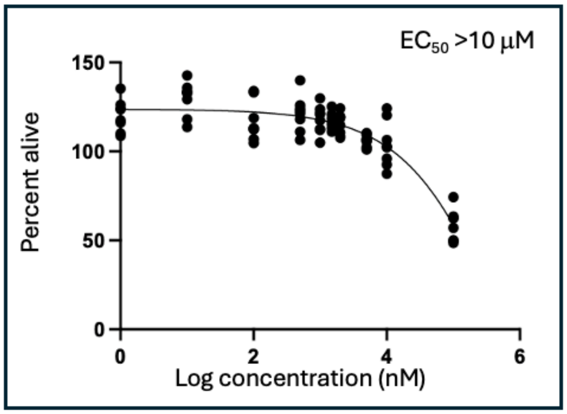

Figure S18c.

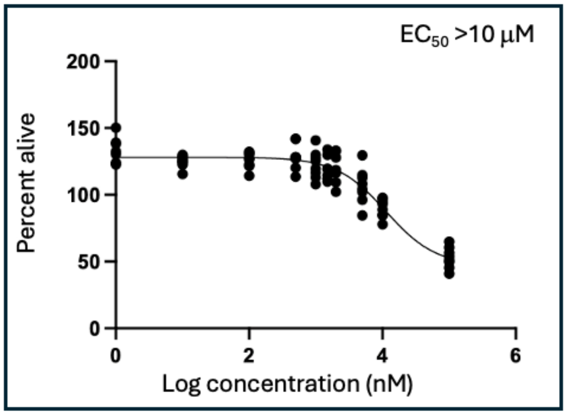

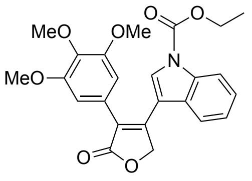

35

Figure S19a.

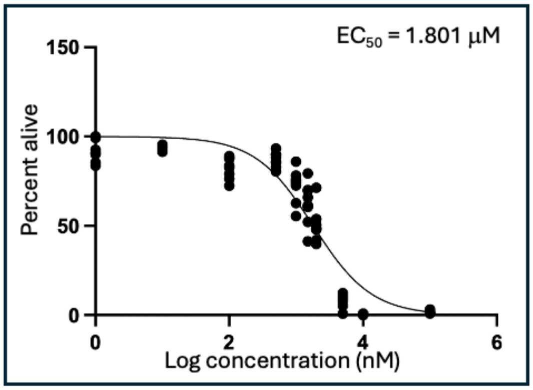

Figure S19d.

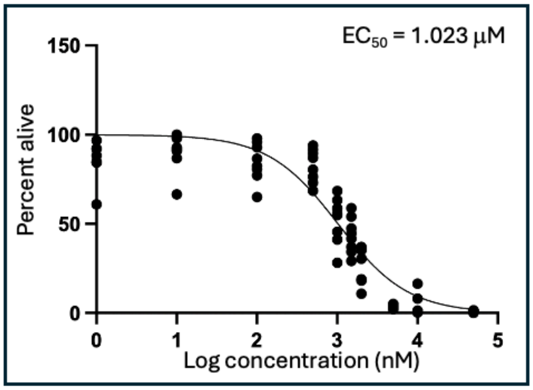

Figure S19b.

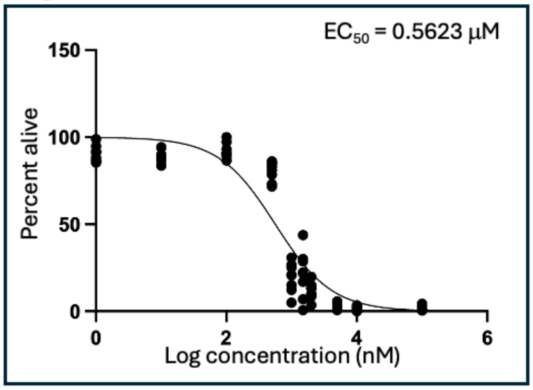

Figure S19e.

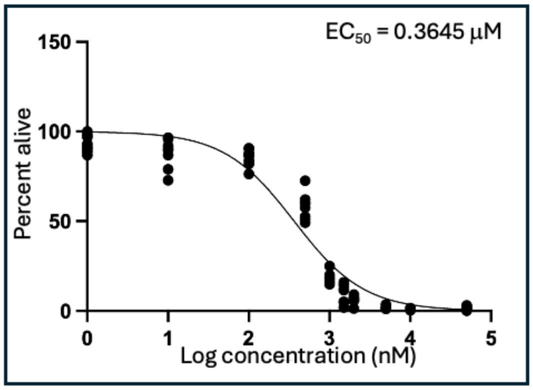

Figure S19c.

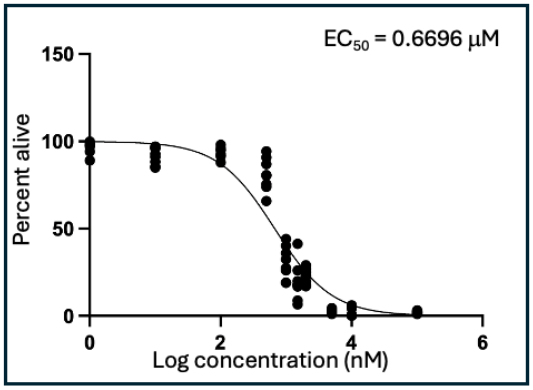

Figure S19f.

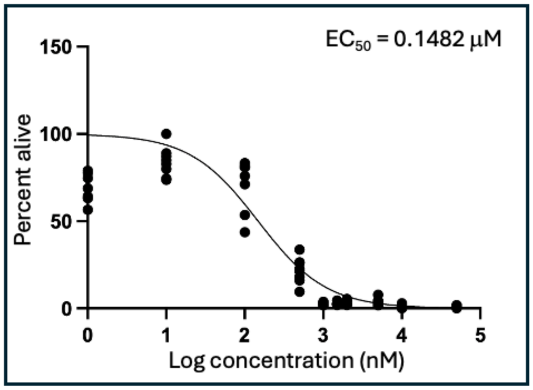

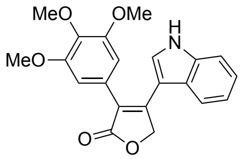

36

Figure S20a.

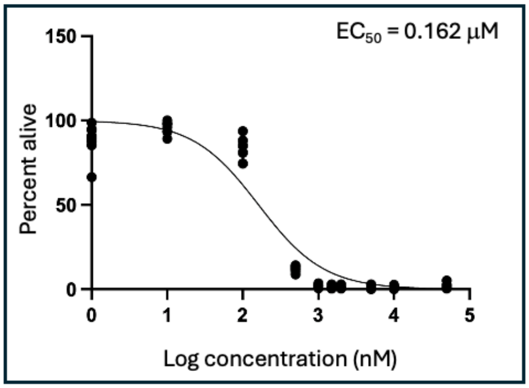

Figure S20d.

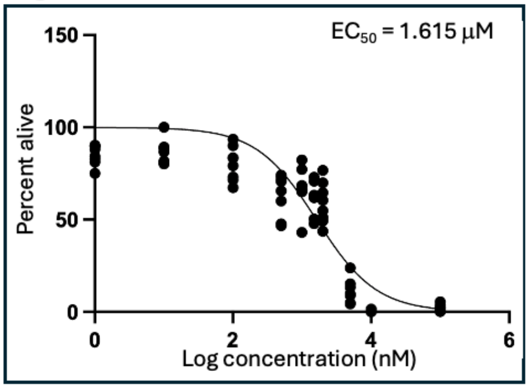

Figure S20b.

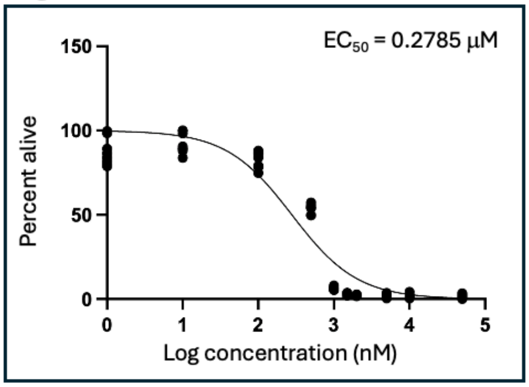

Figure S20c.

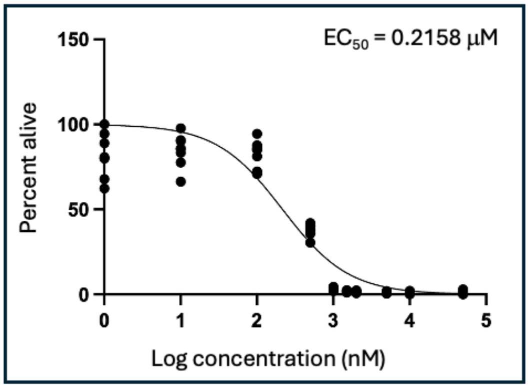

Synthesis.

General Considerations.

THF was dried by passage through a column of alumina utilizing a PureSolv 400 solvent purification system. Dry chlorobenzene was purchased and used as is. ^{1}H NMR and ^{13}C NMR chemical shifts are reported in parts per million (δ) using the solvent's residual proton or carbon signal ($CDCl_3$: δH 7.26 ppm, δC 77.3 ppm; d_6-DMSO: δH 2.50 ppm, δC 39.5 ppm) as an internal reference. Three different NMR spectrometer were used: 400 MHz Varian; 600 MHz AVANCE Bruker; 800 MHz AVANCE HD Bruker. The NMR spectra were analayzed using MestReNova (MNOVA). IR spectra were acquired using a Perkin Elmer Spectrum 100. HRMS were completed with positive electrospray ionization on a Bruker 12 Tesla APEX -Qe FTICR-MS with an Apollo II ion source. Flash chromatography was performed with silica gel (230-400 mesh), and thin-layer chromatography (TLC) was performed with glass-backed silica gel plates and visualized with UV at 254 nm. Commercially available compounds were used as is.

Compounds **3-8**, **13-14**, and **18-19** were available from our previously published work (**Figure S21**).[†,‡]

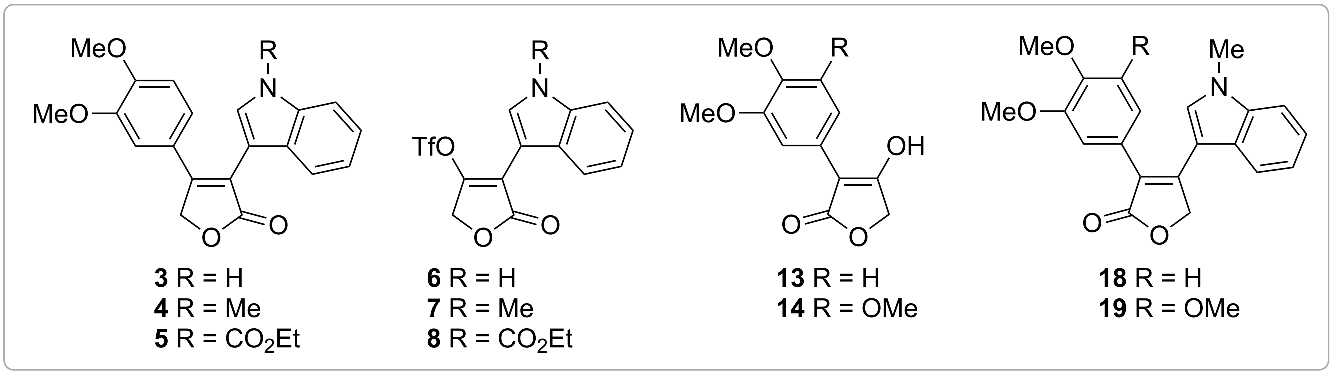

Figure S21. Previously available materials.

[†]*Bioorg. Med. Chem. Lett.* **2021**, *41*, 127991

[‡]*Bioorg. Med. Chem. Lett.* **2023**, *90*, 129347

Scheme S1.

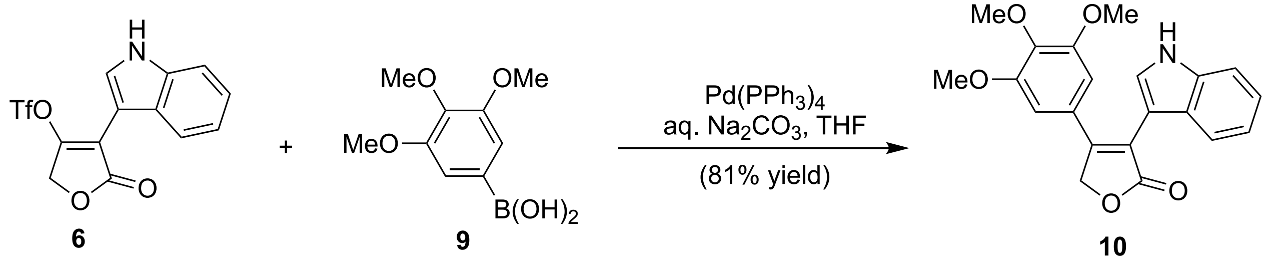

3-(1*H*-Indol-3-yl)-4-(3,4,5-trimethoxyphenyl)furan-2(5*H*)-one (10).

We mostly followed our previously published procedure.† To a rt mixture of previously prepared triflate **6**† (1.00 g, 2.88 mmol) and 3,4,5-trimethoxyphenylboronic acid (**9**) (1.22 g, 5.76 mmol) in THF (25 mL) under Ar was added $Ph(PPh_3)_4$ (0.333 g, 0.288 mmol). An aqueous solution of Na_2CO_3 was prepared by combining Na_2CO_3 (0.916 g, 8.70 mmol) and water 6 mL and this was added to the reaction mixture dropwise. The reaction mixture was stirred at rt for 30 min and then was heated to reflux for 4 h. Upon cooling the reaction mixture to rt, the reaction mixture was filtered through a short plug of silica gel with the aid of EtOAc (25 mL). The organic layer was treated with an aqueous solution of HCl (1.0 M, 100 mL). The mostly aqeuous layer was then extracted with EtOAc (4 x 100 mL). The combined organic layers were then washed with brine (200 mL) and dried with Na_2SO_4. Removal of the solvent vacuo gave a crude brown solid which was purified by flash column chromatography using a gradient of EtOAc/petroleum ether (1:3 to 4:1). Fractions containing the product were combined and concentrated in vacuo giving the desired product **10** (0.85 g, 2.3 mmol, 81% yield) as a tan amorphous solid:

MP 195-200 (dec) °C

R_f = 0.43 (1:1 EtOAc/petroleum ether)

IR (ATR, neat) 3344, 1736 cm^{-1}

^{1}H NMR (400 MHz, D_6-DMSO) δ 11.56 (br s, 1H), 7.69 (d, *J* = 2.8 Hz, 1H), 7.46 (d, *J* = 8.0 Hz, 1H), 7.10 (ddd, *J* = 8.1, 8.0, 1.2 Hz, 1H), 6.86 (ddd, *J* = 8.0, 7.0, 1.0 Hz, 1H), 6.73 (d, *J* = 8.0 Hz, 1H), 6.72 (s, 2H), 5.45 (s, 2H), 3.64 (s, 3H), 3.34 (s, 6H) ppm

^{13}C NMR (100 MHz, D_6-DMSO) δ 174.0, 152.7, 152.5, 138.9, 136.1, 127.3, 126.5, 124.3, 121.5, 120.5, 119.0, 118.3, 112.0, 105.4, 104.4, 70.6, 60.1, 55.3 ppm

HRMS (FTICR-MS) calcd for $C_{21}H_{19}NO_5{\bullet}Na^+$ 388.115544, found 388.115783

†*Bioorg. Med. Chem. Lett.* **2021**, *41*, 127991

Scheme S2.

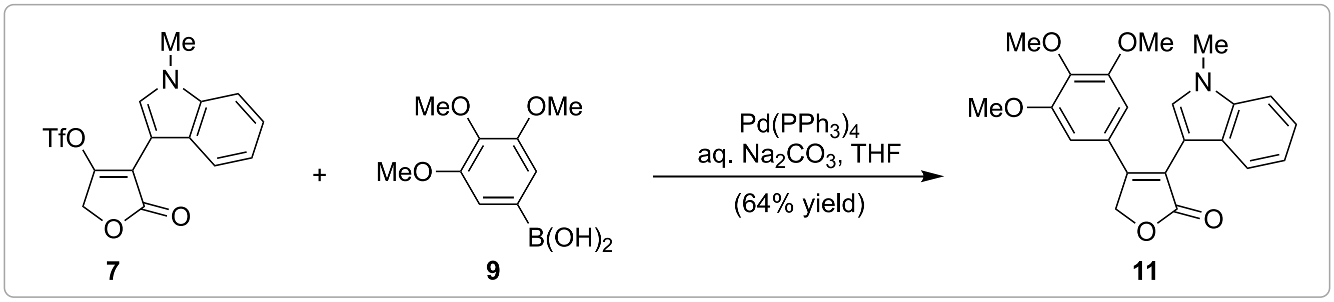

3-(1-methyl-1*H*-indol-3-yl)-4-(3,4,5-trimethoxyphenyl)furan-2(5*H*)-one (11).

We mostly followed our previously published procedure.† To a rt mixture of previously prepared triflate **7**† (1.08 g, 3.00 mmol) and 3,4,5-trimethoxyphenylboronic acid (**9**) (1.27 g, 6.00 mmol) in THF (30 mL) under Ar was added $Ph(PPh_3)_4$ (0.347 g, 0.300 mmol). An aqueous solution of Na_2CO_3 was prepared by combining Na_2CO_3 (0.954 g, 9.00 mmol) and water (6 mL) and this was added to the reaction mixture dropwise. The reaction mixture was stirred at rt for 30 min and then was heated to reflux for 4 h. Upon cooling the reaction mixture to rt, the reaction mixture was filtered through a short plug of silica gel with the aid of EtOAc (25 mL). The organic layer was treated with an aqueous solution of HCl (1.0 M, 200mL). The mostly aqeuous layer was then extracted with EtOAc (3 x 150 mL). The combined organic layers were then washed with brine (400 mL) and dried with Na_2SO_4. Removal of the solvent vacuo gave a crude brown solid (2.0 g) which was purified by flash column chromatography using a gradient of EtOAc/petroleum ether (1:4 to 2:1). Fractions containing the product were combined and concentrated in vacuo giving the desired product **11** (0.73 g, 0.19 mmol, 64% yield) as a tan amorphous solid.

MP 188-191 °C

R_f = 0.47 (1:1 EtOAc/petroleum ether)

IR (ATR, neat) 1744, 1731 cm^{-1}

^{1}H NMR (400 MHz, $CDCl_3$) δ 7.68 (s, 1H), 7.33 (dt, *J* = 8.3, 0.9 Hz, 1H), 7.18 (ddd, *J* = 8.3, 7.0, 1.1 Hz, 1H), 6.92 (ddd, *J* = 8.0, 7.0, 1.0 Hz, 1H), 6.76 (dt, *J* = 8.1, 1.0 Hz, 1H), 5.25 (s, 2H), 3.88 (s, 3H), 3.85 (s, 3H), 3.43 (s, 6H) (s, 6H) ppm

^{13}C NMR (100 MHz, $CDCl_3$) δ 175.0, 153.3, 150.5, 139.9, 137.2, 131.7, 127.1, 125.1, 122.3, 122.2, 120.0, 119.4, 109.8, 105.4, 104.7, 70.9, 61.2, 56.0, 33.5 ppm

HRMS (FTICR-MS) calcd for $C_{22}H_{21}NO_5{\bullet}Na^+$ 402.131193, found 402.131235

†*Bioorg. Med. Chem. Lett.* **2021**, *41*, 127991

Scheme S3.

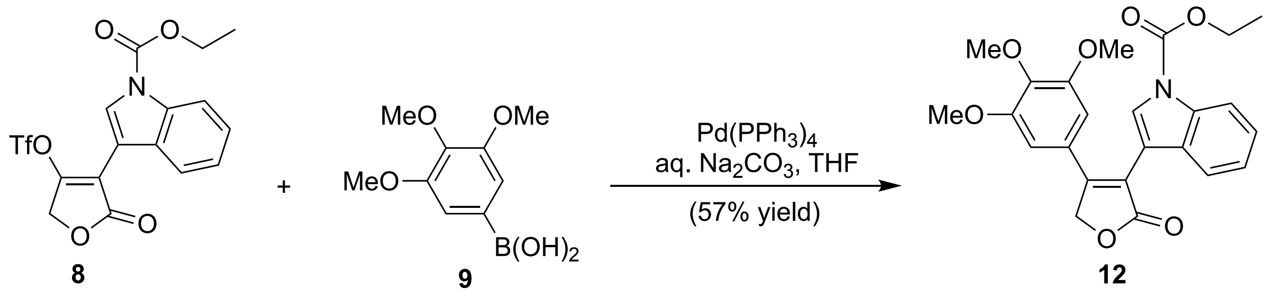

Ethyl 3-(2-oxo-4-(3,4,5-trimethoxyphenyl)-2,5-dihydrofuran-3-yl)-1*H*-indole-1-carboxylate (12). We mostly followed our previously published procedure.† To a rt mixture of previously prepared triflate **8**† (0.39 g, 0.93 mmol) and 3,4,5-trimethoxyphenylboronic acid (**9**) (0.30 g, 1.4 mmol) in THF (10 mL) under Ar was added Ph(PPh_3)$_4$ (110 mg, 0.093 mmol). An aqueous solution of Na_2CO_3 was prepared by combining Na_2CO_3 (0.30 g, 2.8 mmol) and water (1 mL) and this was added to the reaction mixture dropwise. The reaction mixture was stirred at rt for 30 min and then was heated to reflux for 2 h. Upon cooling the reaction mixture to rt, the reaction mixture was filtered through a short plug of silica gel with the aid of EtOAc (25 mL). The organic layer was treated with an aqueous solution of HCl (1.0 M, 25 mL). The mostly aqeuous layer was then extracted with EtOAc (3 x 40 mL). The combined organic layers were then washed with brine (100 mL) and dried with Na_2SO_4. Removal of the solvent vacuo gave a crude brown solid (0.88 g) which was purified by flash column chromatography using a gradient of EtOAc/petroleum ether (1:3 to 1:1). Fractions containing the product were combined and concentrated in vacuo giving the desired product **12** (230 mg, 0.53 mmol, 57% yield) as a yellow/orange oil which solidified upon standing. An analytical sample was obtained by recystallization from EtOH which gave **12** as light yellow crystals:

MP 148-149 °C

R_f = 0.56 (1:1 EtOAc/petroleum ether)

IR (ATR, neat) 1745, 1733, 1645 cm^{-1}

^{1}H NMR (800 MHz, D_6-DMSO) δ 8.18 (d, J = 8.0 Hz, 1H), 8.00 (s, 1H), 7.35 (ddd, J = 8.4, 7.3, 1.1 Hz), 7.11 (ddd, J = 8.1, 7.2, 1.0 Hz, 1H), 6.88 (dd, J = 8.0, 0.8 Hz, 1H), 6.71 (s, 2H), 5.52 (s, 2H), 4.49 (q, J = 7.2 Hz, 2H), 3.65 (s, 3H), 3.39 (s, 6H), 1.42 (t, J = 7.2 Hz, 3H) ppm

^{13}C NMR (200 MHz, D_6-DMSO) δ 173.1, 157.3, 152.7, 150.1, 139.5, 134.6, 127.1, 126.6, 125.6, 124.9, 122.7, 121.0, 116.2, 114.9, 110.8, 105.8, 70.9, 63.7, 60.1, 55.5, 14.1 ppm

HRMS (FTICR-MS) calcd for $C_{24}H_{23}NO_7$•Na^+ 460.136673, found 460.136646

†*Bioorg. Med. Chem. Lett.* **2021**, *41*, 127991

Scheme S4.

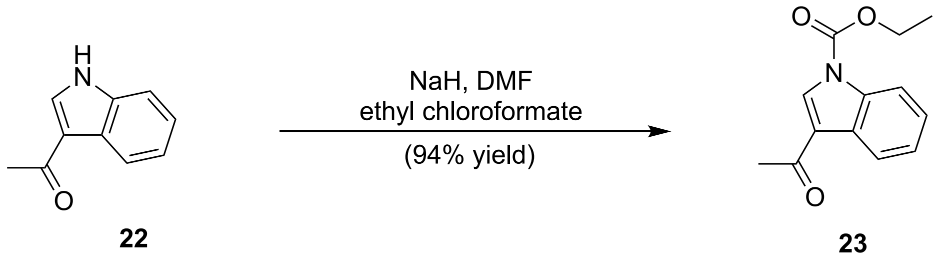

Ethyl 3-acetyl-1*H*-indole-1-carboxylate (23).

Compound **23** is known,† and we prepared this compound by adapting the procedure by Coudert and co-workers for the acylation of indole.‡ To a 0 °C stirred mixture of 3-acetylindole (**22**) (3.18 g, 20.0 mmol) and DMF (100 mL), dry sodium hydride (0.72 g, 30.0 mmol, caution: highly flammable) was added and this was allowed to stir for 15 min. To this, neat ethyl chloroformate (2.87 mL, 30.0 mmol) was added dropwise via syringe. The reaction was allowed to warm slowly to room temperature by letting the ice bath melt and stirring was continued for 20 h. The reaction was quenched by pouring into ice/water mixture (100 mL) and the resulting aqueous layer was extracted with diethyl ether (4 x 100 mL). The combined organic layers were washed with brine (400 mL) and dried over sodium sulfate. Removal of the solvent in vacuo gave the desired product **23** as a white amorphous solid (4.33 g, 18.7 mmol, 94% yield). The spectral data was consistent with the literature.

MP 115-117 °C

R_f = 0.36 (1:4 EtOAc/petroleum ether)

IR (ATR, neat) 1748, 1660 cm^{-1}

^{1}H NMR (400 MHz, $CDCl_3$) δ 8.36-8.39 (m, 1H), 8.26 (s, 1H), 8.15-8.17 (m, 1H), 7.35-7.43 (m, 2H), 4.57 (q, J = 7.2 Hz, 2H), 2.58 (s, 3H), 1.52 (t, J = 7.2 Hz, 3H) ppm

^{13}C NMR (100 MHz, $CDCl_3$) δ 194.1, 150.8, 135.8, 132.3, 127.6, 126.0, 124.9, 123.0, 121.4, 115.2, 64.5, 28.0, 14.6 ppm

CAS# 1211498-82-6

†*Angew. Chem. Int. Ed.* **2012**, *51*, 8304

‡*Tetrahedron* **2004**, *60*, 10039

Scheme S5.

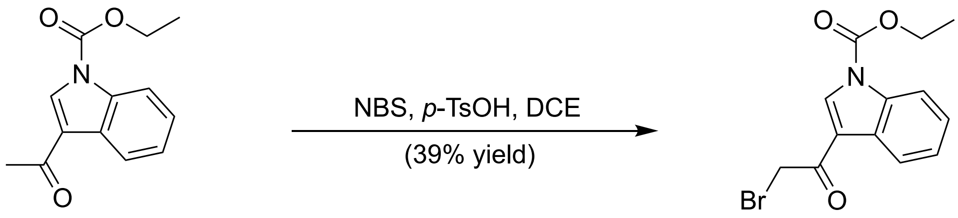

23 **24**

Ethyl 3-(2-bromoacetyl)-1*H*-indole-1-carboxylate (24).

A modification of a procedure by Mattson and co-workers that gave α-bromoacetophenone was utilized.† Indole **23** (1.26 g, 5.44 mmol), *N*-bromosuccinimide (5.98 g, 5.98 mmol), *p*-TsOH (0.190 g, 1.00 mmol) and DCE (100 mL) were combined and the mixture was heated at 70 °C for 1 h. The reaction was allowed to cool to rt and then washed with water (100 mL), saturated bicarbonate (100 mL), and brine (100 mL). Removal of the solvent in vacuo the crude product as an orange amorphous solid. Purification by flash column chromatography (gradient: 1:30 to 1:15 EtOAc/petroleum ether) gave the desired product **24** as a white amorphous solid (1.20 g, 3.87 mmol, 39% yield):

MP 110-112 °C

R_f = 0.45 (1:4 EtOAc/petroleum ether)

IR (ATR, neat) 1732, 1652 cm^{-1}

^{1}H NMR (400 MHz, $CDCl_3$) δ 8.38 (s, 1H), 8.34-8.37 (m, 1H), 8.17-8.19 (m, 1H), 7.40-7.45 (m, 2H), 4.59 (q, *J* = 7.2 Hz. 2H), 4.36 (s, 2H), 1.53 (t, *J* = 7.2 Hz, 3H) ppm

^{13}C NMR (100 MHz, $CDCl_3$) δ 187.5, 150.6, 135.8, 132.8, 127.5, 126.4, 125.2, 123.0, 118.1, 115.3, 64.8, 31.7, 14.6 ppm

HRMS (FTICR-MS) calcd for $C_{13}H_{12}BrNO_3$•Na^+ 331.989277, found 331.989297

†*Angew. Chem. Int. Ed.* **2014**, *53*, 14538

Scheme S6.

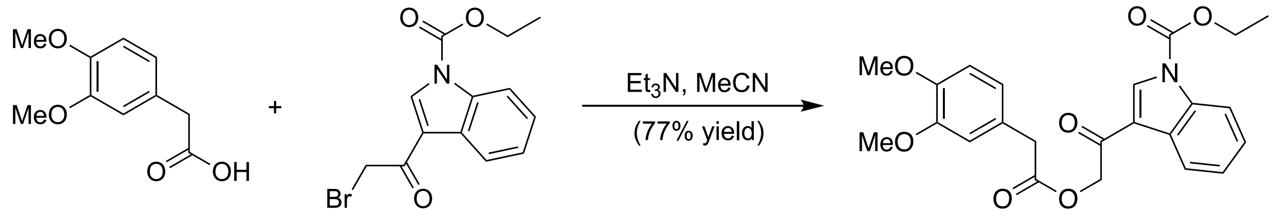

25 **24** **27**

Ethyl 3-(2-(2-(3,4-dimethoxyphenyl)acetoxy)acetyl)-1*H*-indole-1-carboxylate (27).

A modification of a procedure by Tron and co-workers leading to diaryl ketoesters was utilized.† To a rt stirred solution of 3,4-dimethoxyphenylacetic acid (**25**) (0.51 g, 2.6 mmol) and bromoketone **24** (0.40 g, 1.3 mmol) dissolved in MeCN (10 mL) was added Et_3N (0.54 mL, 3.9 mmol). The reaction mixture was stirred at rt for 18 h by which time TLC (1:1 EtOAc/petroleum ether) showed the disappearance of **24**. The solvent was removed in vacuo and the resulting residue was taken up in diethyl ether (50 mL). The organic layer was washed with an aqueous solution of $KHSO_4$ (1.0 M, 50 mL), a saturated aqueous solution of $NaHCO_3$ (50 mL), and brine (50 mL). The organic layer was then dried with Na_2SO_4. Removal of the solvent in vacuo gave the desired product **27** as a light orange amorphous solid (0.44 g, 1.0 mmol, 77% yield):

MP 90-96 °C

R_f = 0.53 (1:1 EtOAc/petroleum ether)

IR (ATR, neat) 1753, 1728, 1669 cm^{-1}

^{1}H NMR (400 MHz, D_6-DMSO) δ 8.75 (s, 1H), 8.14-8.20 (m, 2H), 7.45 (ddd, *J* = 8.4, 7.3, 1.4 Hz, 1H), 7.39 (dt, *J* = 7.4, 1.2 Hz, 1H), 6.96 (d, *J* = 2.0 Hz, 1H), 6.90 (d, *J* = 8.2 Hz, 1H), 6.84 (dd, *J* = 8.2, 2.0 Hz), 5.41 (s, 2H), 4.52 (q, *J* = 7.2 Hz, 2H), 3.76 (s, 2H), 3.75 (s, 3H), 3.73 (s, 3H), 1.43 (t, *J* = 7.2 Hz, 3H) ppm

^{13}C NMR (100 MHz, D_6-DMSO) δ 189.2, 171.0, 149.9, 148.6, 147.8, 134.8, 133.2, 126.7, 126.4, 125.8, 124.6, 121.7, 121.5, 116.3, 114.9, 113.2, 111.7, 66.6, 64.4, 55.5, 55.4, 14.0 ppm

HRMS (FTICR-MS) calcd for $C_{23}H_{23}NO_7$•Na^+ 448.136673, found 448.136705

†*J. Med. Chem.* **2006**, *49*, 5372

Scheme S7.

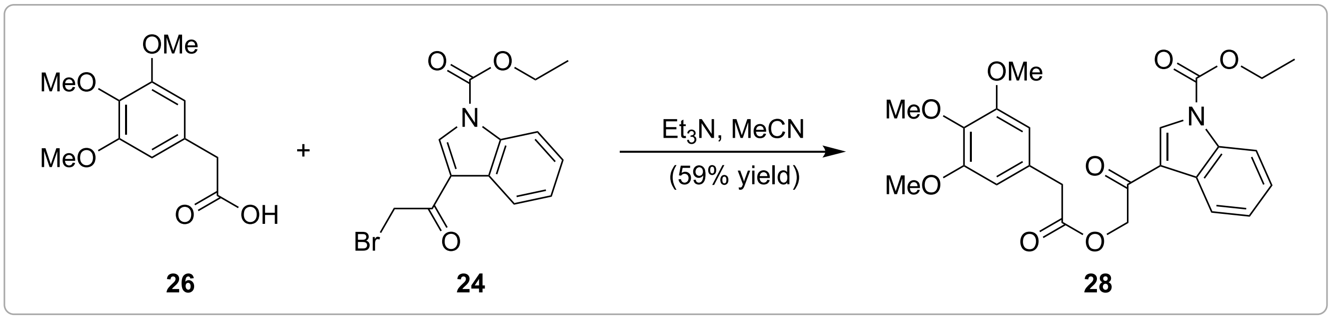

Ethyl 3-(2-(2-(3,4,5-trimethoxyphenyl)acetoxy)acetyl)-1*H*-indole-1-carboxylate (28).

A modification of a procedure by Tron and co-workers leading to diaryl ketoesters was utilized.†. To a rt stirred solution of 3,4,5-trimethoxyphenylacetic acid (**26**) (0.474 g, 2.10 mmol) and bromoketone **24** (0.325 g, 1.05 mmol) dissolved in MeCN (10 mL) was added Et_3N (0.44 mL, 3.1 mmol). The reaction mixture was stirred at rt for 25 h by which time TLC (1:1 EtOAc/petroleum ether) showed the disappearance of **24**. The solvent was removed in vacuo and the resulting residue was taken up in diethyl ether (50 mL). The organic layer was washed with an aqueous solution of $KHSO_4$ (1.0 M, 50 mL), a saturated aqueous solution of $NaHCO_3$ (50 mL), and brine (50 mL). The organic layer was then dried with Na_2SO_4. Removal of the solvent in vacuo gave the desired product **28** as a yellow powder (0.281 g, 0.617 mmol, 59% yield):

MP 121-126 °C

R_f = 0.50 (1:1 EtOAc/petroleum ether)

IR (ATR, neat) 1738, 1701, 1680 cm^{-1}

^{1}H NMR (400 MHz, D_6-DMSO) δ 8.78 (s, 1H), 8.19 (ddd, *J* = 7.7, 1.5, 0.7 Hz, 1H), 8.16 (dt, *J* = 8.2, 1.0 Hz, 1H), 7.46 (ddd, *J* = 8.4, 7.2, 1.4 Hz, 1H), 7.39 (ddd, *J* = 7.8, 7.3, 1.2 Hz, 1H), 6.69 (s, 2H), 5.44 (s, 2H), 4.52 (q, *J* = 7.2 Hz, 2H), 3.78 (s, 6H), 3.65 (s, 3H), 1.44 (q, *J* = 7.2 Hz, 3H) ppm

^{13}C NMR (100 MHz, D_6-DMSO) δ 189.2, 170.7, 152.7, 149.9, 136.3, 134.8, 133.2, 129.7, 126.7, 125.8, 124.6, 121.6, 116.3, 114.9, 106.7, 66.6, 64.3, 59.9, 55.8, 14.0 ppm

HRMS (FTICR-MS) calcd for $C_{24}H_{25}NO_8{\bullet}Na^+$ 478.147237, found 478.147264

†*J. Med. Chem.* **2006**, *49*, 5372

Scheme S8.

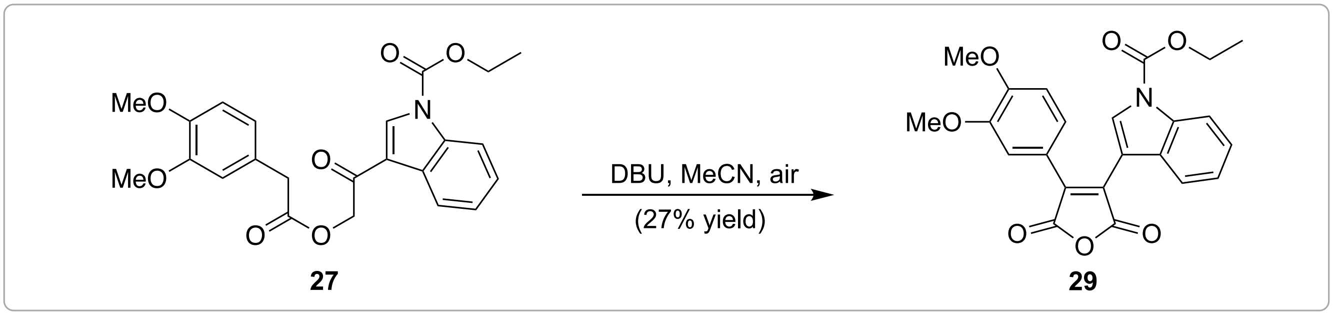

Ethyl 3-(4-(3,4-dimethoxyphenyl)-2,5-dioxo-2,5-dihydrofuran-3-yl)-1*H*-indole-1-carboxylate (29). A modification of a procedure leading to 3,4-diarylmaleic anhydrides by Pal and co-workers was followed.† To a mixture of ketoester **27** (0.279 g, 0.656 mmol) in MeCN (10 mL) was added DBU (0.31 mL, 2.0 mmol). Open to the air, the mixture was stirred for 5 h. The solvent was removed in vacuo and the residue was taken up in EtOAc (60 mL). The organic layer was washed with an aqueous solution of $KHSO_4$ (1.0 M, 50 mL), brine (50 mL), and the dried over Na_2SO_4. Removal of the solvent in vacuo gave an orange amorphous solid (0.25 g), which was purified by flash column chromatography using a gradient of EtOAc/petroleum ether (1:15 to 1:6). Fractions containing the product were combined and concentrated in vacuo to give the desired product **29** as an orange amorphous solid (77 mg, 0.18 mmol, 27% yield):

MP 185-188 °C
R_f = 0.51 (1:2 EtOAc/petroleum ether)
IR (ATR, neat) 1826, 1742 cm^{-1}
^{1}H NMR (400 MHz, $CDCl_3$) δ 8.33 (s, 1H), 8.25 (d, *J* = 8.4 Hz, 1H), 7.35 (dd, *J* = 8.5, 2.0 Hz, 1H), 7.31 (ddd, *J* = 8.4, 7.3, 1.1 Hz, 1H), 7.03 (d, *J* = 2.1 Hz, 1H), 6.98 (ddd, *J* = 8.1, 7.2, 1.0 Hz), 6.83 (d, *J* = 8.4 Hz), 6.57 (d, *J* = 8.0 Hz, 1H), 4.57 (q, *J* = 7.2 Hz, 2H), 3.90 (s, 3H), 3.43 (s, 3H), 1.52 (t, *J* = 7.2 Hz, 3H) ppm
^{13}C NMR (100 MHz, $CDCl_3$) δ 165.63, 165.61, 151.9, 150.5, 148.8, 135.8, 134.9, 130.6, 129.3, 125.9, 125.8, 124.8, 123.7, 122.5, 120.8, 115.8, 113.1, 111.1, 109.6, 64.4, 56.2, 55.8, 14.6 ppm
HRMS (FTICR-MS) calcd for $C_{23}H_{19}NO_7{\bullet}Na^+$ 444.105373, found 444.105447

†*Synlett* **2002**, 13, 947

Scheme S9.

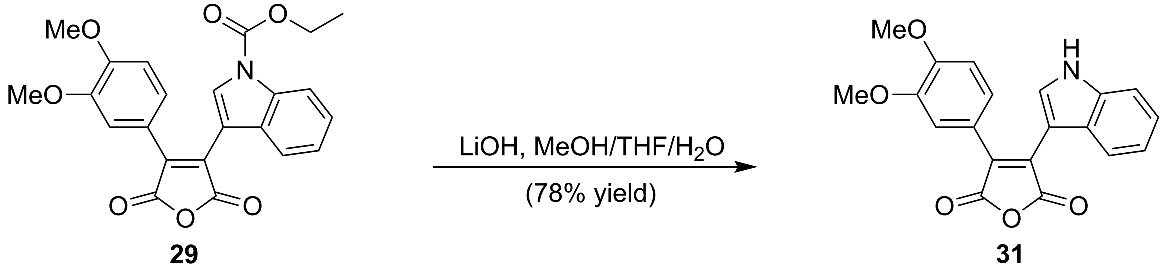

3-(3,4-Dimethoxyphenyl)-4-(1*H*-indol-3-yl)furan-2,5-dione (31).

A modification of a literature procedure involving the deprotection of *N*-carboethoxy-substituted fused indoles by Kurth and co-workers was followed.† To a rt stirred heterogenous mixture of anhydride **29** (120 mg, 0.28 mmol) in 3 mL MeOH/3 mL THF/1.5 mL H_2O was added LiOH monohydrate (35 mg, 0.84 mmol). The reaction mixture was stirred at rt for 22 h. The reaction mixture was treated with an aqueous solution of HCl (1.0 M, 15 mL) and then extracted with EtOAc (3 x 20 mL). The combined organic layers were washed with brine (50 mL) and dried over Na_2SO_4. Removal of the solvent in vacuo a crude orange oil (0.14 g), which was purified by flash column chromatography using a gradient of EtOAc/petroleum ether (1:8 to 1:2). Fractions containing the product were combined and concentrated in vacuo to give the desired product **31** as a bright red powder (76 mg, 0.21 mmol, 78% yield):

MP 195-197 °C

R_f = 0.27 (1:2 EtOAc/petroleum ether)

IR (ATR, neat) 3313, 1824, 1747, 1737 cm^{-1}

^{1}H NMR (400 MHz, $CDCl_3$) δ 8.93 (br s, 1H), 8.10 (d, *J* = 3.0 Hz, 1H), 7.41-7.44 (m, 1H), 7.33 (ddd, *J* = 8.5, 2.1, 0.6 Hz, 1H), 7.18-7.22 (m, 1H), 7.06 (d, *J* = 2.0 Hz), 6.89-6.93 (m, 1H), 6.86 (d, *J* = 8.4 Hz, 1H), 6.55 (d, *J* = 8.1 Hz, 1H), 3.91 (s, 3H), 3.46 (s, 3H) ppm

^{13}C NMR (100 MHz, $CDCl_3$) δ 166.7, 166.4, 151.0, 148.6, 136.5, 131.6, 131.3, 130.4, 124.2, 123.8, 123.0, 121.7, 113.0, 112.1, 111.0, 105.9, 56.2, 55.8 ppm

HRMS (FTICR-MS) calcd for $C_{20}H_{15}NO_5$•Na^+ 372.084243, found 372.084268

†*Tetrahedron Lett.* **2015**, *56*, 5429

Scheme S10.

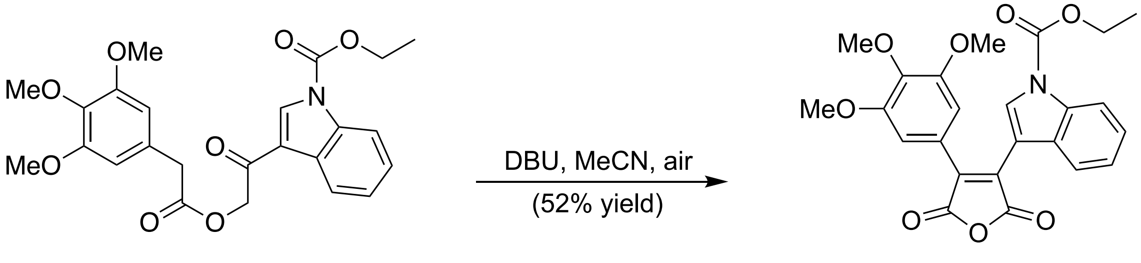

Ethyl 3-(2,5-dioxo-4-(3,4,5-trimethoxyphenyl)-2,5-dihydrofuran-3-yl)-1*H*-indole-1-carboxylate (30).

A modification of a procedure leading to 3,4-diarylmaleic anhydrides by Pal and co-workers was followed.† To a mixture of ketoester **28** (0.581 g, 1.28 mmol) in MeCN (40 mL) was added DBU (0.55 mL, 3.8 mmol). Open to the air, the mixture was stirred for 6 h. The solvent was removed in vacuo and the residue was taken up in EtOAc (40 mL). The organic layer was washed with an aqueous solution of $KHSO_4$ (1.0 M, 40 mL), brine (40 mL), and the dried over Na_2SO_4. Removal of the solvent in vacuo gave an orange amorphous solid. Trituration of the solid with diethyl ether (~10 mL) gave the desired product **30** as a yellow amorphous solid (0.282 g, 0.669 mmol, 52% yield):

MP 210-214 °C

R_f = 0.59 (1:2 EtOAc/petroleum ether)

IR (ATR, neat) 1825, 1758, 1742, 1621 cm^{-1}

^{1}H NMR (400 MHz, $CDCl_3$) δ 8.39 (s, 1H), 8.25 (d, *J* = 8.4 Hz, 1H), 7.32 (ddd, *J* = 8.5, 7.3, 1.2 Hz, 1H), 7.00 (ddd, *J* = 8.2, 7.2, 1.0 Hz, 1H), 6.84 (s, 2H), 6.52 (d, *J* = 8.0 Hz, 1H), 4.58 (q, *J* = 7.2 Hz, 2H), 3.88 (s, 3H), 3.49 (s, 6H), 1.53 (t, *J* = 7.2 Hz, 3H) ppm

^{13}C NMR (100 MHz, $CDCl_3$) δ 165.44, 165.41, 152.3, 150.5, 141.0, 135.8, 134.5, 131.1, 130.7, 126.0, 125.7, 123.7, 123.1, 122.7, 115.8, 109.5, 108.3, 64.5, 61.3, 56.2, 14.7 ppm

HRMS (FTICR-MS) calcd for $C_{24}H_{21}NO_8{\bullet}Na^+$ 474.115937, found 474.115978

†*Synlett* **2002**, 13, 947

Scheme S11.

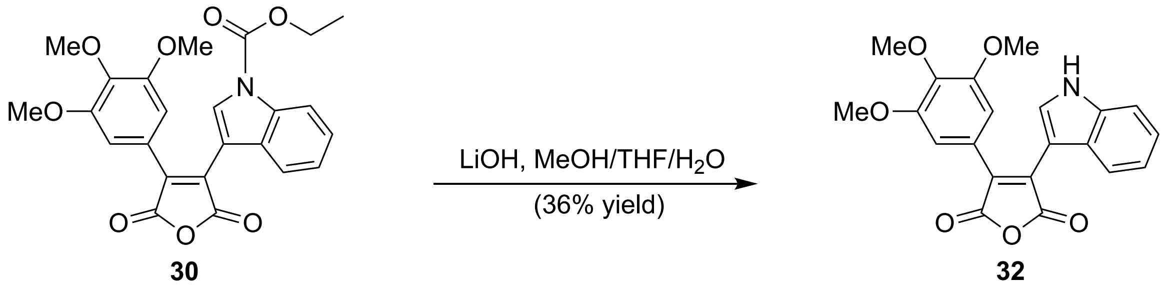

3-(1*H*-Indol-3-yl)-4-(3,4,5-trimethoxyphenyl)furan-2,5-dione (32).

A modification of a literature procedure involving the deprotection of *N*-carboethoxy-substituted fused indoles by Kurth and co-workers was followed.† To a rt stirred heterogenous mixture of anhydride **30** (90. mg, 0.20 mmol) in 2 mL MeOH/2 mL THF/1 mL H_2O was added LiOH monohydrate (24 mg, 0.60 mmol). The reaction mixture was stirred at rt for 23 h. The reaction mixture was treated with an aqueous solution of HCl (1.0 M, 10 mL) and then extracted with EtOAc (3 x 20 mL). The combined organic layers were washed with brine (50 mL) and dried over Na_2SO_4. Removal of the solvent in vacuo a crude orange oil (74 mg), which was purified by flash column chromatography using a gradient of EtOAc/petroleum ether (1:8 to 1:2). Fractions containing the product were combined and concentrated in vacuo to give the desired product **32** as a bright orange amorphous solid (27 mg, 0.071 mmol, 36% yield):

MP 194-197 °C

R_f = 0.27 (1:2 EtOAc/petroleum ether)

IR (ATR, neat) 3277, 1816, 1748 cm^{-1}

^{1}H NMR (400 MHz, $CDCl_3$) δ 8.91 (br s, 1H), 8.15 (d, *J* = 3.0 Hz), 7.43 (d, *J* = 8.1 Hz, 1H), 7.21 (ddd, *J* = 8.1, 7.0, 1.1 Hz, 1H), 6.92 (ddd, *J* = 8.1, 7.0, 1.0 Hz, 1H), 6.84 (s, 2H), 6.49 (d, *J* = 8.2 Hz, 1H), 3.81 (s, 3H), 3.51 (s, 6H) ppm

^{13}C NMR (100 MHz, $CDCl_3$) δ 166.5, 166.1, 153.1, 140.1, 136.5, 132.8, 131.7, 129.9, 124.1, 124.0, 123.6, 123.2, 121.7, 112.1, 108.0, 106.0, 61.3, 56.2 ppm

HRMS (FTICR-MS) calcd for $C_{21}H_{17}NO_6{\bullet}Na^+$ 402.094808, found 402.094826

†*Tetrahedron Lett.* **2015**, *56*, 5429

Scheme S12.

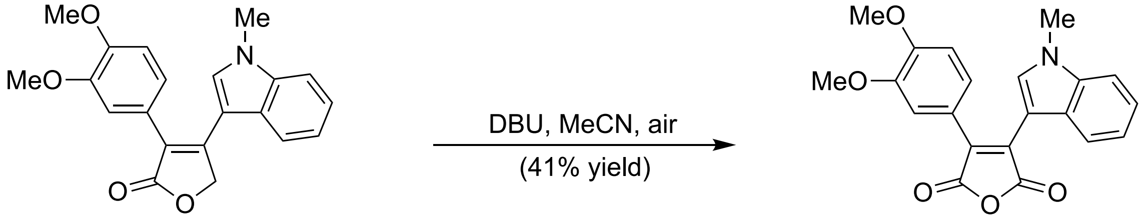

5 **33**

3-(3,4-Dimethoxyphenyl)-4-(1-methyl-1*H*-indol-3-yl)furan-2,5-dione (33).

To a rt stirred solution of furanone **5** (0.18 g, 0.52 mmol) in MeCN (10 mL) in a 20 mL reaction vial was added DBU (0.24 mL, 1.6 mmol) dropwise via syringe. The reaction mixture was stirred at rt open to the air for 16 h. The solvent was transferred to a round-bottom with the aid of MeOH and solvent was removed in vacuo giving a dark red oil. The oil was treated with aqueous solution of HCl (1.0 M, 15 mL). The solid that formed was isolated via filtration and dried in vacuo. The product was obtained as a bright orange amorphous solid (140 mg). Trituration of the solid with diethyl ether gave **33** as a dark orange powder (77 mg, 0.21 mmol, 41% yield):

MP 211-214 °C

R_f = 0.35 (1:2 EtOAc/petroleum ether)

IR (ATR, neat) 1817, 1744 cm^{-1}

^{1}H NMR (600 MHz, D_6-DMSO) δ 8.16 (s, 1H), 7.55 (d, J = 8.2 Hz, 1H), 7.18-7.22 (m, 2H), 7.01 (d, J = 8.5 Hz, 1H), 6.97 (d, J = 2.0 Hz, 1H), 6.86 (ddd, J = 8.1, 7.0, 1.0 Hz, 1H), 6.44 (d, J = 8.2 Hz, 1H), 3.94 (s, 3H), 3.79 (s, 3H), 3.31 (s, 3H) ppm

^{13}C NMR (150 MHz, D_6-DMSO) δ 166.1, 165.9, 150.1, 147.9, 137.2, 135.8, 131.6, 128.5, 123.9, 123.2, 122.6, 122.2, 121.4, 120.5, 112.9, 111.4, 110.9, 103.0, 55.6, 55.0, 33.2 ppm

HRMS (FTICR-MS) calcd for $C_{21}H_{17}NO_5{\bullet}Na^+$ 386.099893, found 386.099929

CAS# 1239911-48-8 (mentioned in the literature but not experimentally described)†

†*Bioorg. Med. Chem. Lett.* **2010**, *20*, 1693

Scheme S13.

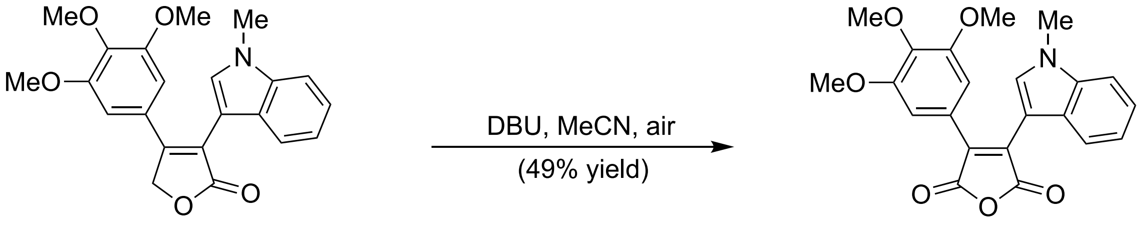

3-(1-Methyl-1*H*-indol-3-yl)-4-(3,4,5-trimethoxyphenyl)furan-2,5-dione (34).

To a rt stirred solution of furanone **11** (0.100 g, 0.263 mmol) in MeCN (10 mL) in a 20 mL reaction vial was added DBU (0.13 mL, 0.87 mmol) dropwise via syringe. The reaction mixture was stirred at rt open to the air for 16 h. The solvent was transferred round-bottom with the aid of MeOH and the solvent was removed in vacuo giving a dark red oil. The crude material was treated with an aqueous solution of HCl (1.0 M, 15 mL). The solid that formed was isolated via filtration and dried in vacuo. The product was obtained as a red amorphous solid (91 mg). Trituration of the solid with diethyl ether gave **34** as a bright red powder (51 mg, 0.13 mmol, 49% yield):

MP 170-174 °C (lit.† MP 175-177 °C)

R_f = 0.38 (1:2 EtOAc/petroleum ether)

IR (ATR, neat) 1813, 1745 cm^{-1}

^{1}H NMR (600 MHz, D_6-DMSO) δ 8.23 (s, 1H), 7.57 (d, J = 8.2 Hz), 1H), 7.22 (ddd, J = 8.2, 7.1, 1.1 Hz, 1H), 6.87 (ddd, J = 8.1, 7.1, 1.1 Hz, 1H), 6.33 (d, J = 8.0 Hz), 6.77 (s, 2H), 3.95, (s, 3H), 3.71 (s, 3H), 3.43 (s, 6H) ppm

^{13}C NMR (150 MHz, D_6-DMSO) δ 166.0, 165.7, 152.4, 137.2, 136.5, 132.9, 127.7, 124.2, 123.8, 122.7, 122.0, 120.6, 111.0, 107.6, 105.5, 103.0, 60.2, 55.6, 39.9, 33.2 ppm

HRMS (FTICR-MS) calcd for $C_{22}H_{19}NO_6{\bullet}Na^+$ 416.110458, found 416.110508

CAS# 2250360-33-7

†*Pharmaceuticals* **2017**, 10, 62

Scheme S14.

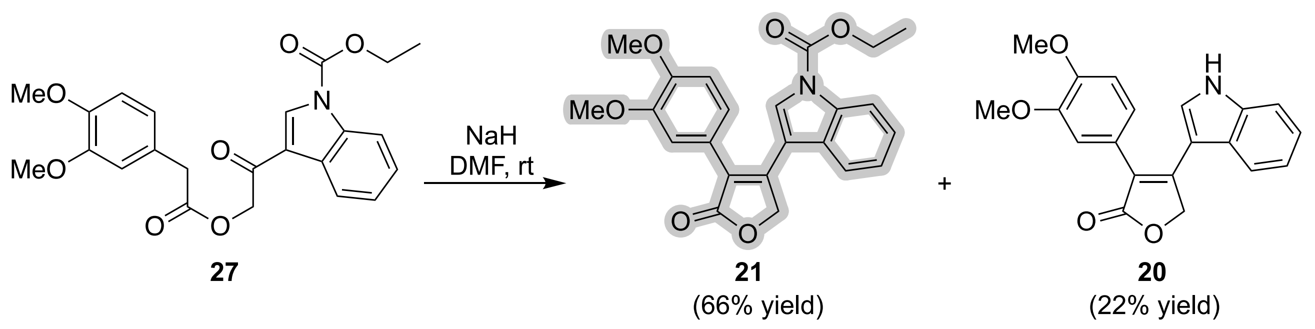

Ethyl 3-(4-(3,4-dimethoxyphenyl)-5-oxo-2,5-dihydrofuran-3-yl)-1*H*-indole-1-carboxylate (21).

A modification of a literature used to prepared 3,4-diarylfuranones by Knaus and co-workers was followed.† To a rt stirred solution of ketoester **27** (213 mg, 0.500 mmol) in DMF (5 mL) was added dry NaH (24 mg, 1.0 mmol, caution: flammable). The reaction mixture was stirred at rt for 1 h and then quenched by the addition of an aqueous solution of HCl (1.0 M, 20 mL). The aqueous layer was extracted with EtOAc (4 x 20 mL). The combined organic layers were washed with brine (60 mL) and dried over Na_2SO_4. Removal of the solvent in vacuo gave an oil that contained DMF. The crude oil was taken up in ether (100 mL) and then washed with water (60 mL), brine (100 mL) and dried over Na_2SO_4. Removal of the solvent in vacuo gave a crude orange amorphous solid (0.218 g). ^{1}H NMR analysis of the crude solid showed a mixture of **20** and **21**. Purification by flash column chromatography (gradient: 1:5 to 2:1 EtOAc/petroleum ether). Fractions containing **21** were combined and the solvent removed in vacuo to give the product **21** as a yellow oil (135 mg, 0.331 mmol, 66% yield). An analytical sample was obtained after recrystallization from EtOH to give **21** as bright yellow crystals:

MP 162-163 °C

R_f = 0.62 (1:1 EtOAc/petroleum ether)

IR (ATR, neat) 1740, 1640 cm^{-1}

^{1}H NMR (400 MHz, D_6-DMSO) δ 8.23 (s, 1H), 8.14 (dt, *J* = 8.4, 0.9 Hz, 1H), 7.33 (ddd, *J* = 8.4, 7.2, 1.2 Hz, 1H), 7.01-7.06 (m, 2H), 6.94 (d, *J* = 8.4 Hz, 1H), 6.92 (d, *J* = 2.0 Hz, 1H), 6.79 (dt, *J* = 8.0, 1.0 Hz, 1H), 5.40 (s, 2H), 4.49 (q, *J* = 7.2 Hz, 2H), 3.75 (s, 3H), 3.40 (s, 3H), 1.41 (t, *J* = 7.2 Hz, 3H) ppm

^{13}C NMR (100 MHz, D_6-DMSO) δ 172.9, 150.1, 149.9, 149.1, 148.2, 135.0, 127.1, 126.1, 125.1, 123.3, 123.1, 123.0, 121.8, 121.5, 114.9, 112.7, 112.6, 111.5, 70.6, 63.9, 55.5, 55.2, 14.0 ppm

HRMS (FTICR-MS) calcd for $C_{23}H_{21}NO_6{\bullet}Na^+$ 430.126108, found 430.126115

†*J. Heterocycl. Chem.* **2003**, *40*, 861

Scheme S15.

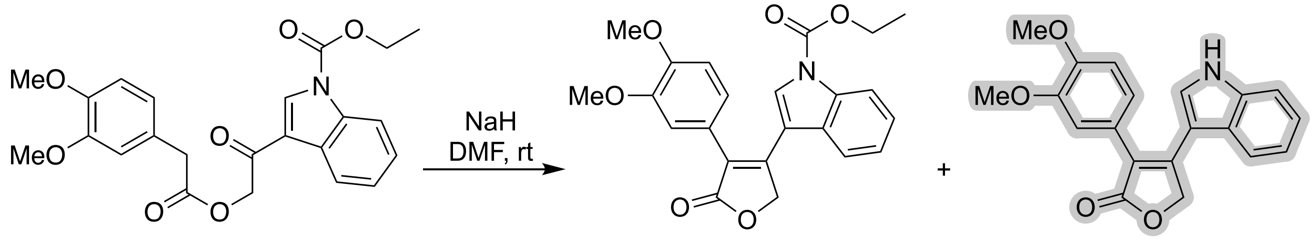

3-(3,4-Dimethoxyphenyl)-4-(1*H*-indol-3-yl)furan-2(5*H*)-one (20).

The procedure used to make **21** also produced lower R_f product **20**, which derived from the loss of the carboethoxy group from **21**. Chromatographic fractions containing **20** were combined and the solvent removed in vacuo to give deprotected indole **20** as an orange-brown amorphous solid (37 mg, 0.11 mmol, 22% yield). An analytical sample was obtained after recrystallization from EtOH to give **20** as a tan powder:

MP 202-206 °C

R_f = 0.30 (1:1 EtOAc/petroleum ether)

IR (ATR, neat) 3219, 1705 cm^{-1}

^{1}H NMR (400 MHz, D_6-DMSO) δ 11.83 (br s, 1H), 7.72 (d, J = 3.2 Hz, 1H), 7.45 (dt, J = 8.2, 0.9 Hz, 1H), 7.12 (ddd, J = 8.2, 6.2, 2.0 Hz, 1H), 6.99-7.01 (m, 2H), 6.88-6.93 (m, 3H), 5.41 (s, 2H), 3.78 (s, 3H), 3.48 (s, 3H) ppm

^{13}C NMR (100 MHz, D_6-DMSO) δ 173.6, 153.1, 148.6, 148.3, 136.5, 128.3, 124.3, 123.9, 122.2, 121.8, 120.9, 120.3, 118.2, 112.8, 112.3, 111.7, 106.9, 70.4, 55.5, 55.2 ppm

HRMS (FTICR-MS) calcd for $C_{20}H_{17}NO_4$•Na^+ 358.104979, found 358.104980

Scheme S16.

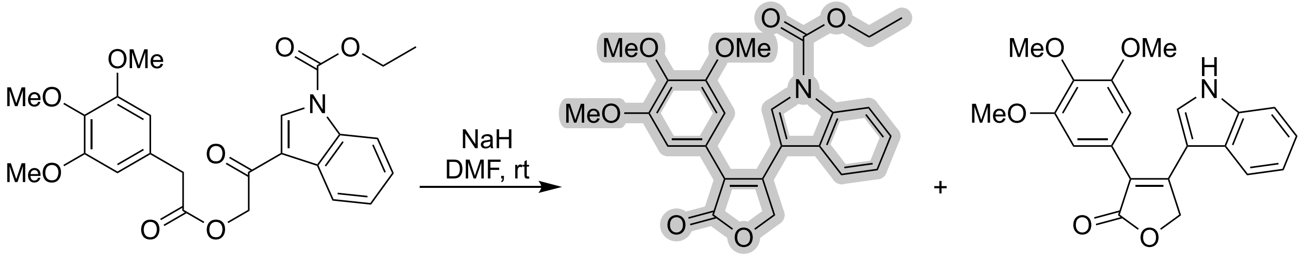

28 **35** (36% yield) **36** (19% yield)

Ethyl 3-(5-oxo-4-(3,4,5-trimethoxyphenyl)-2,5-dihydrofuran-3-yl)-1*H*-indole-1-carboxylate (35). A modification of a literature used to prepared 3,4-diarylfuranones by Knaus and co-workers was followed.† To a rt stirred solution of ketoester **28** (138 mg, 0.300 mmol) in DMF (5 mL) was added dry NaH (14 mg, 0.60 mmol, caution: flammable). The reaction mixture was stirred at rt for 1 h and then quenched by the addition of an aqueous solution of HCl (1.0 M, 20 mL). The aqueous layer was extracted with EtOAc (4 x 20 mL). The combined organic layers were washed with brine (60 mL) and dried over Na_2SO_4. Removal of the solvent in vacuo gave an oil that contained DMF. The crude oil was taken up in ether (60 mL) and then washed with water (50 mL), brine (50 mL) and dried over Na_2SO_4. Removal of the solvent in vacuo gave a crude orange amorphous solid (121 mg). ^{1}H NMR analysis of the crude solid showed a mixture of **35** and **36**. Purification by flash column chromatography (gradient: 1:4 to 2:1 EtOAc/petroleum ether). Fractions containing **35** were combined and the solvent removed in vacuo to give the product **35** as a yellow oil (47 mg, 0.11 mmol, 36% yield). An analytical sample was obtained after recrystallization from EtOH to give **35** as yellow needles:

MP 145-148 °C

R_f = 0.65 (1:1 EtOAc/petroleum ether)

IR (ATR, neat) 1741, 1645 cm^{-1}

^{1}H NMR (400 MHz, D_6-DMSO) δ 8.26 (s, 1H), 8.15 (d, *J* = 8.4 Hz, 1H), 7.35 (ddd, *J* = 8.4, 7.2, 1.2 Hz, 1H), 7.06 (ddd, *J* = 8.2, 7.2, 1.0 Hz, 1H), 6.78 (d, *J* = 8.0 Hz, 1H), 6.71 (s, 2H), 5.45 (s, 2H), 4.48 (q, *J* = 7.2 Hz, 2H), 3.67 (s, 3H), 3.48 (s, 6H), 1.41 (t, *J* = 7.2 Hz, 3H) ppm

^{13}C NMR (100 MHz, D_6-DMSO) δ 172.7, 152.6, 151.2, 149.9, 137.9, 134.9, 127.5, 126.00, 125.97, 125.2, 123.2, 123.0, 121.6, 114.9, 112.3, 106.8, 70.6, 63.9, 60.1, 55.7, 14.0 ppm

HRMS (FTICR-MS) calcd for $C_{24}H_{23}NO_7$•Na^+ 460.136673, found 460.136686

†*J. Heterocycl. Chem.* **2003**, *40*, 861

Scheme S17.

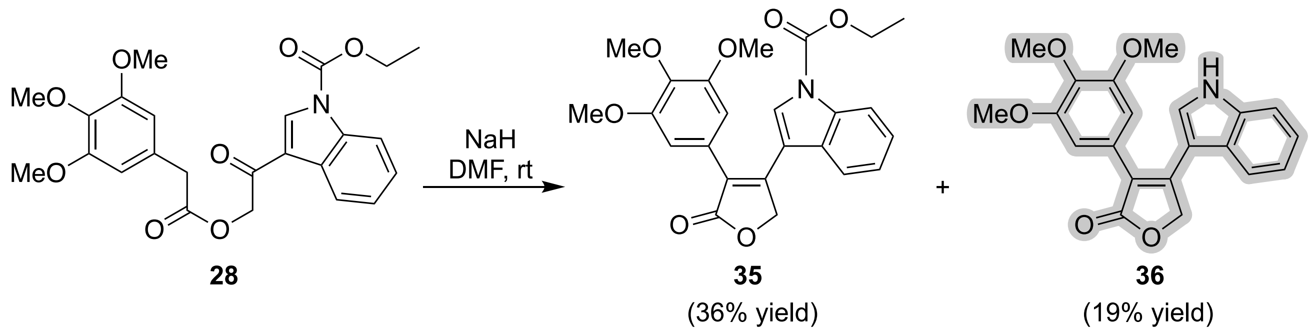

4-(1*H*-Indol-3-yl)-3-(3,4,5-trimethoxyphenyl)furan-2(5*H*)-one (36).

The procedure used to make **35** also produced lower R_f product **36**, which derived from the loss of the carboethoxy group from **35**. Chromatographic fractions containing **36** were combined and then removal of the solvent in vacuo gave **36** as an orange oily solid (21 mg, 0.057 mmol, 19% yield). An analytical sample was obtained after recrystallization from EtOH to give **36** as a tan powder:

MP 215-218 (dec) °C

R_f = 0.30 (1:1 EtOAc/petroleum ether)

IR (ATR, neat) 3206, 1702 cm^{-1}

^{1}H NMR (400 MHz, D_6-DMSO) δ 11.87 (br s, 1H), 7.76 (d, *J* = 3.0 Hz, 1H), 7.45 (dt, *J* = 8.2, 1.0 Hz, 1H), 7.14 (ddd, *J* = 8.1, 7.0, 1.1 Hz, 1H), 6.91 (ddd, *J* = 8.1, 7.0, 1.1 Hz, 1H), 6.82 (d, *J* = 8.1 Hz, 1H), 6.69 (s, 2H), 5.44 (s, 2H), 3.70 (s, 3H), 3.54 (s, 6H) ppm

^{13}C NMR (100 MHz, D_6-DMSO) δ 173.4, 153.9, 152.7, 137.3, 136.5, 128.7, 127.4, 123.8, 122.3, 121.0, 120.3, 118.1, 112.3, 106.8, 106.7, 70.3, 60.1, 55.7 ppm

HRMS (FTICR-MS) calcd for $C_{21}H_{19}NO_5{\bullet}Na^+$ 388.115543, found 388.115547

Figure S22a: ^{1}H NMR of 10

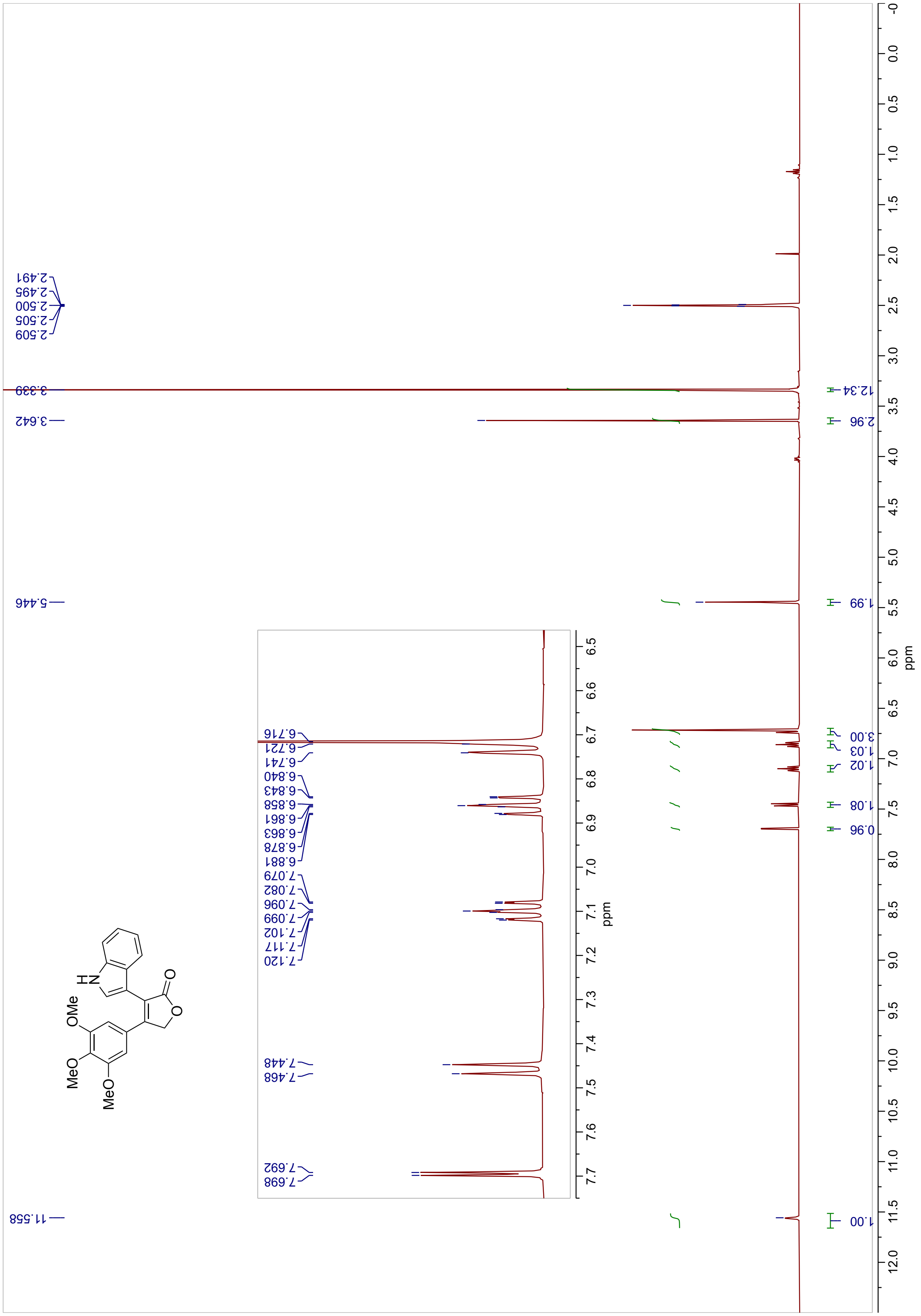

Figure S22b: ^{13}C NMR of 10

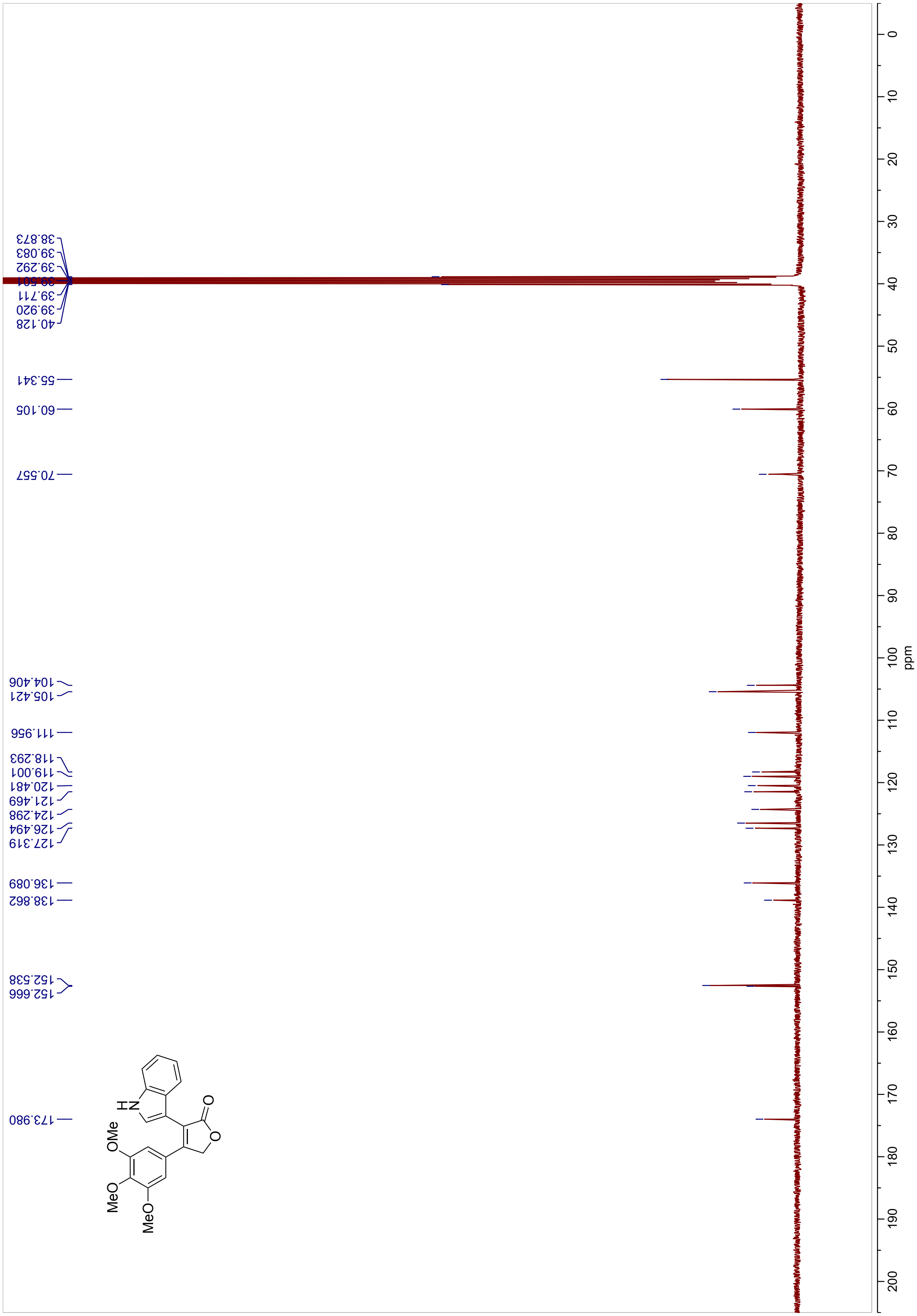

Figure S23a: ^{1}H NMR of 11

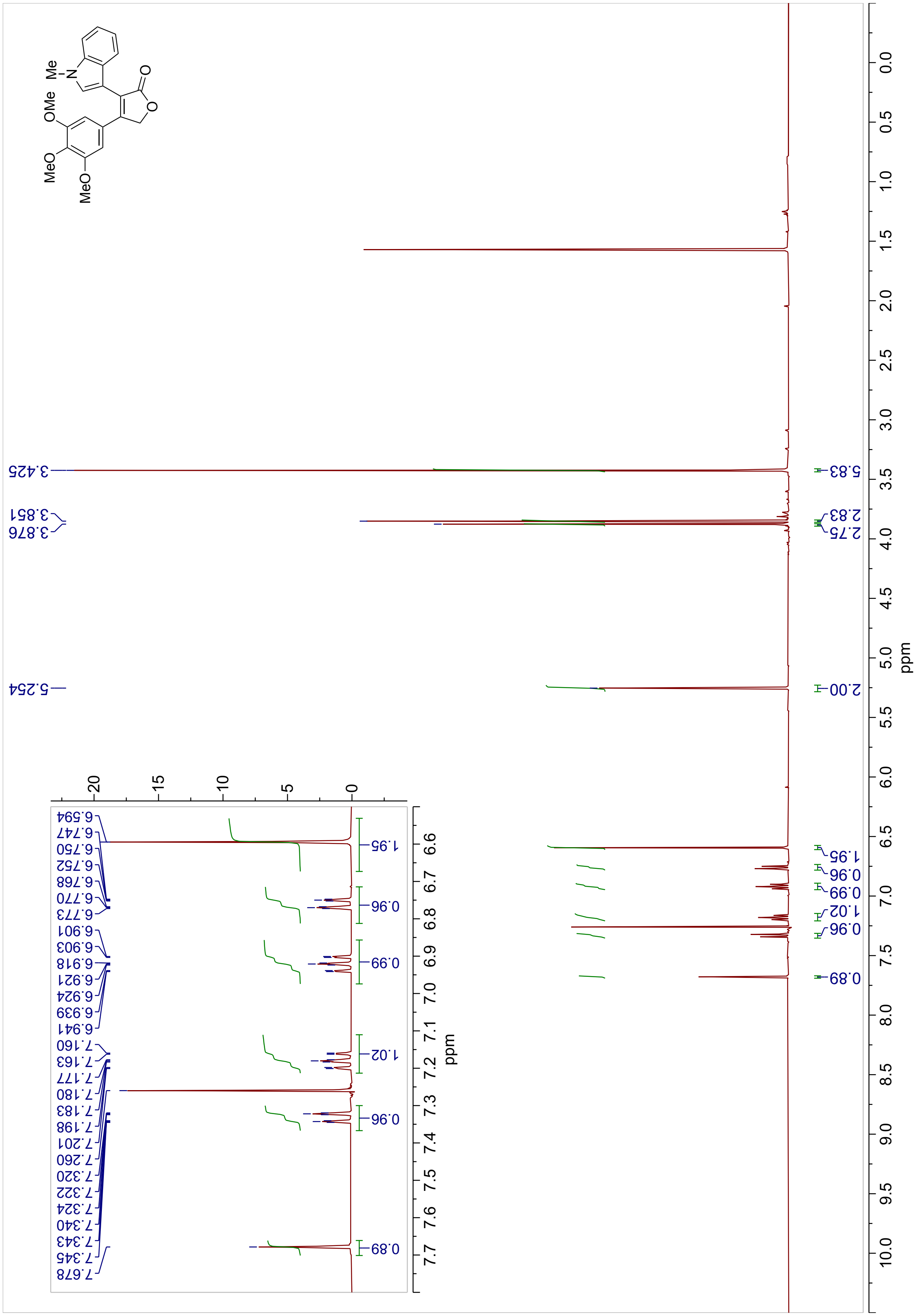

Figure S23b: ^{13}C NMR of 11

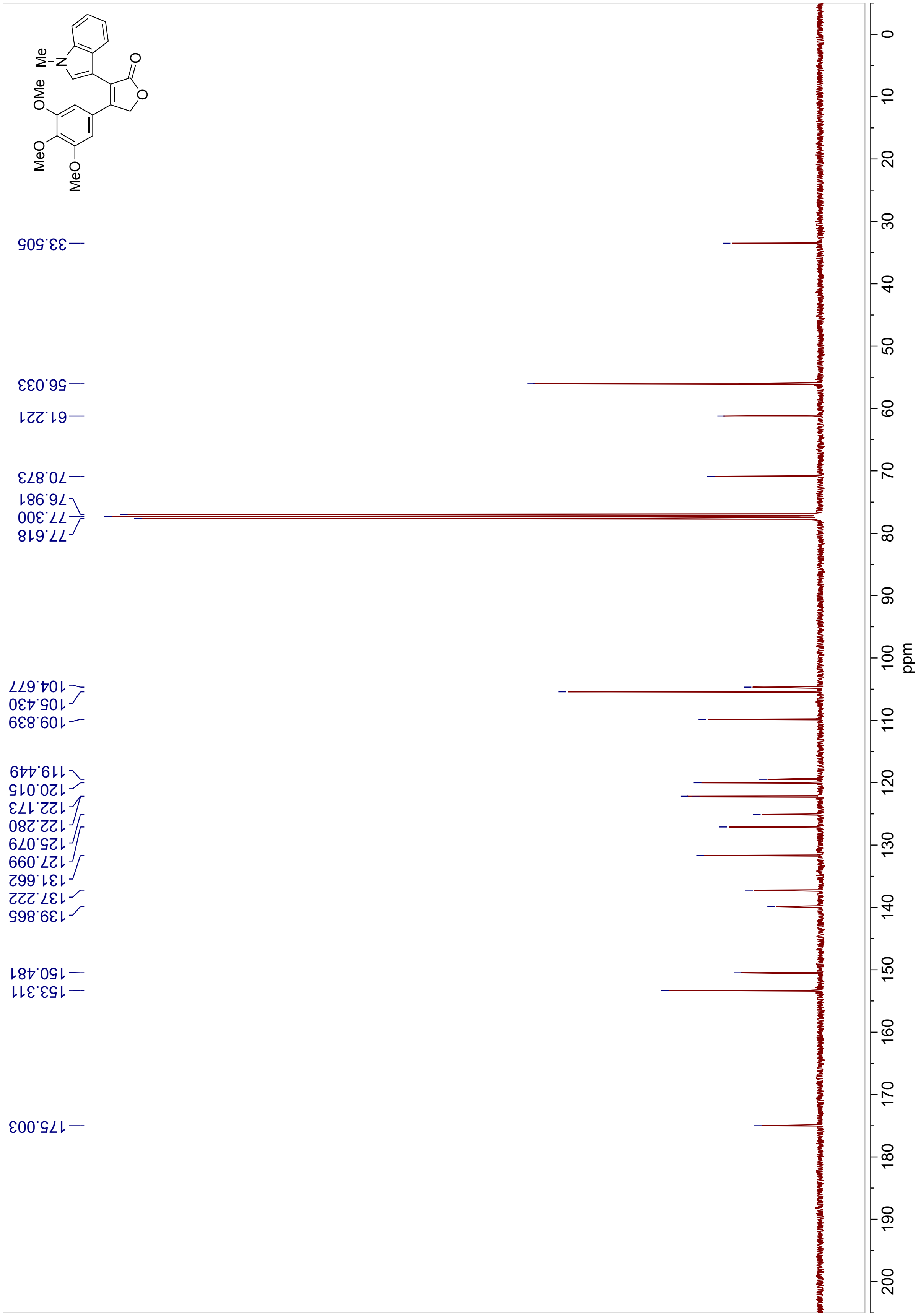

Figure S24a: ^{1}H NMR of 12

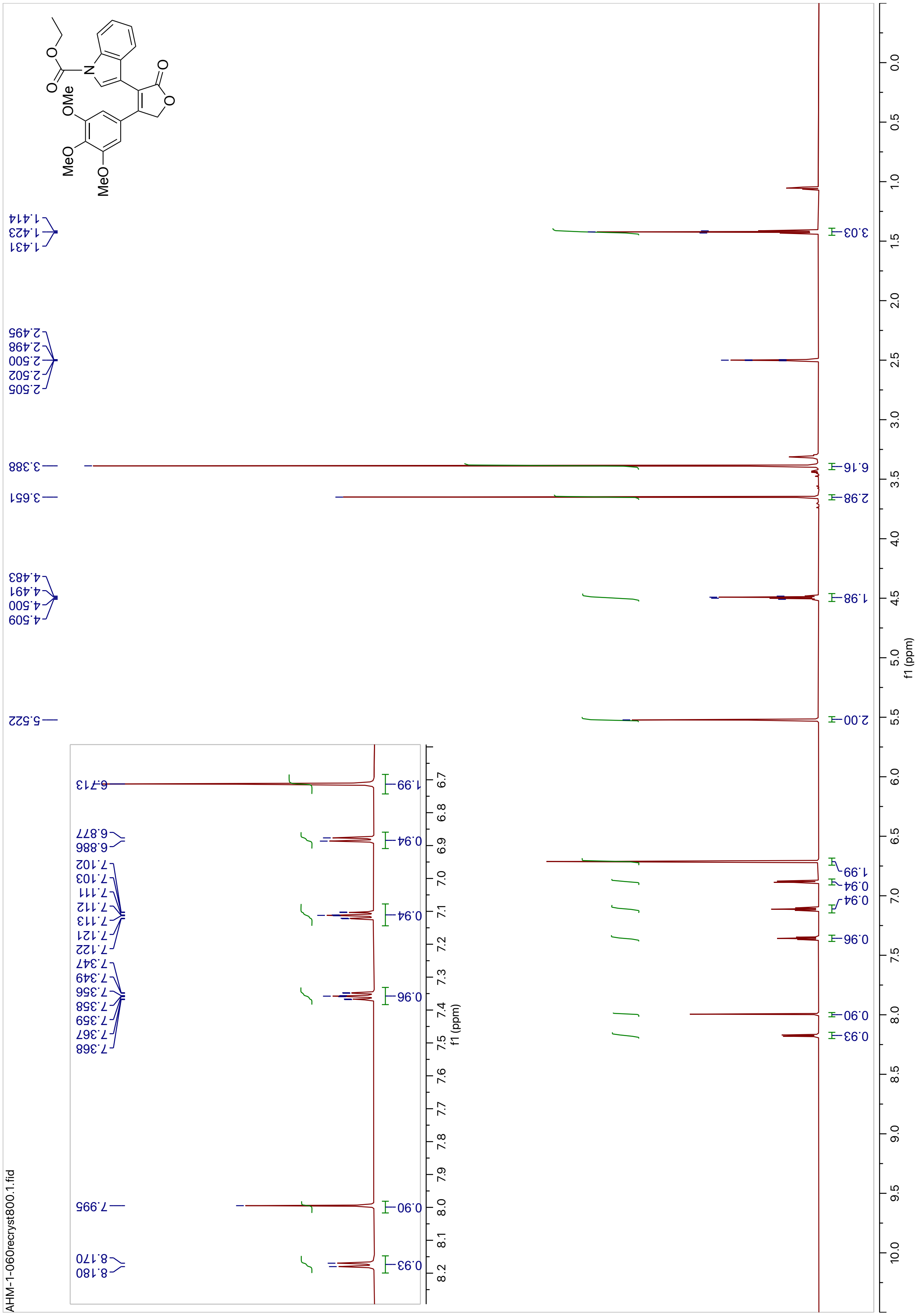

Figure S24b: ^{13}C NMR of 12

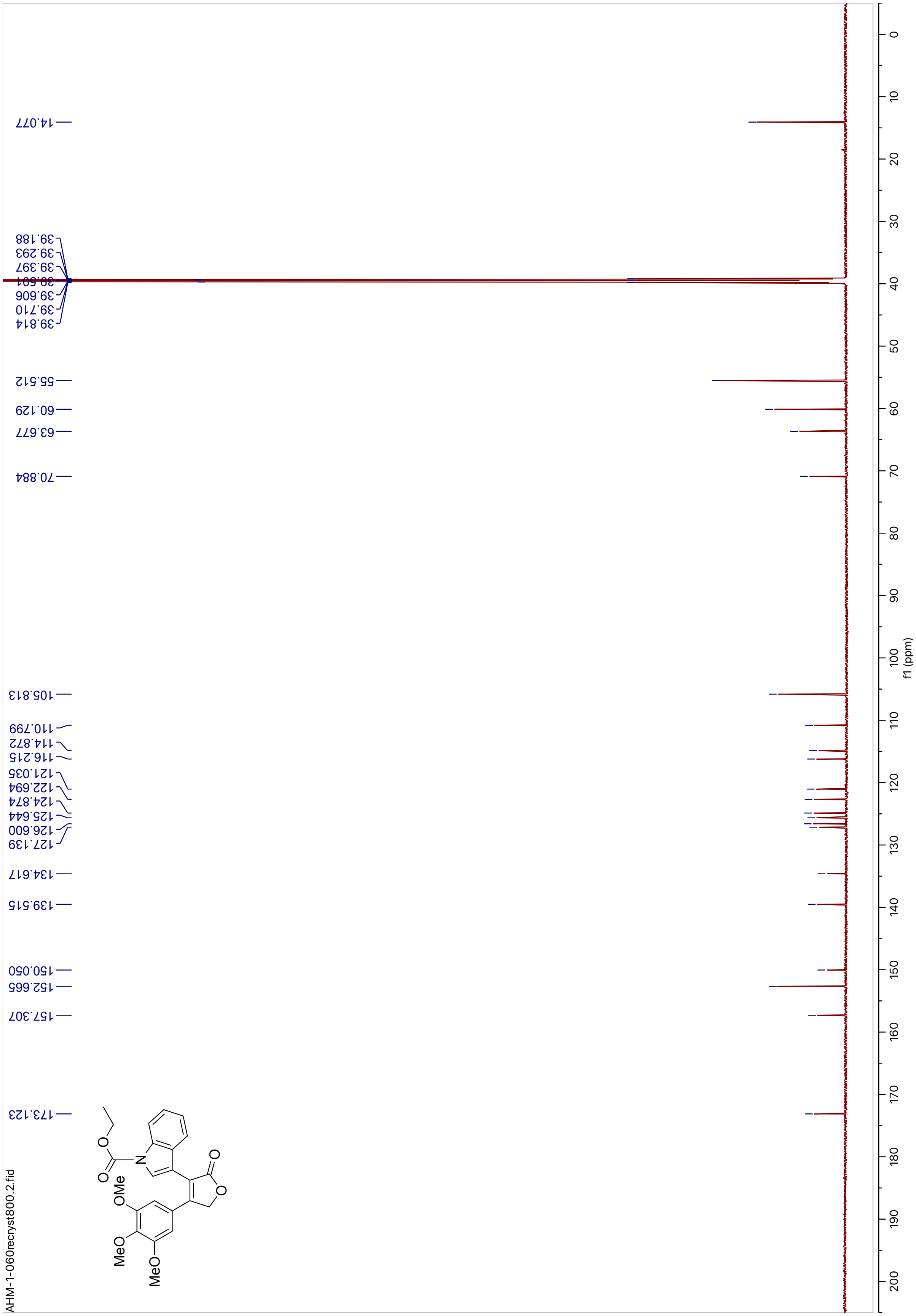

Figure S25a: ^{1}H NMR of 20

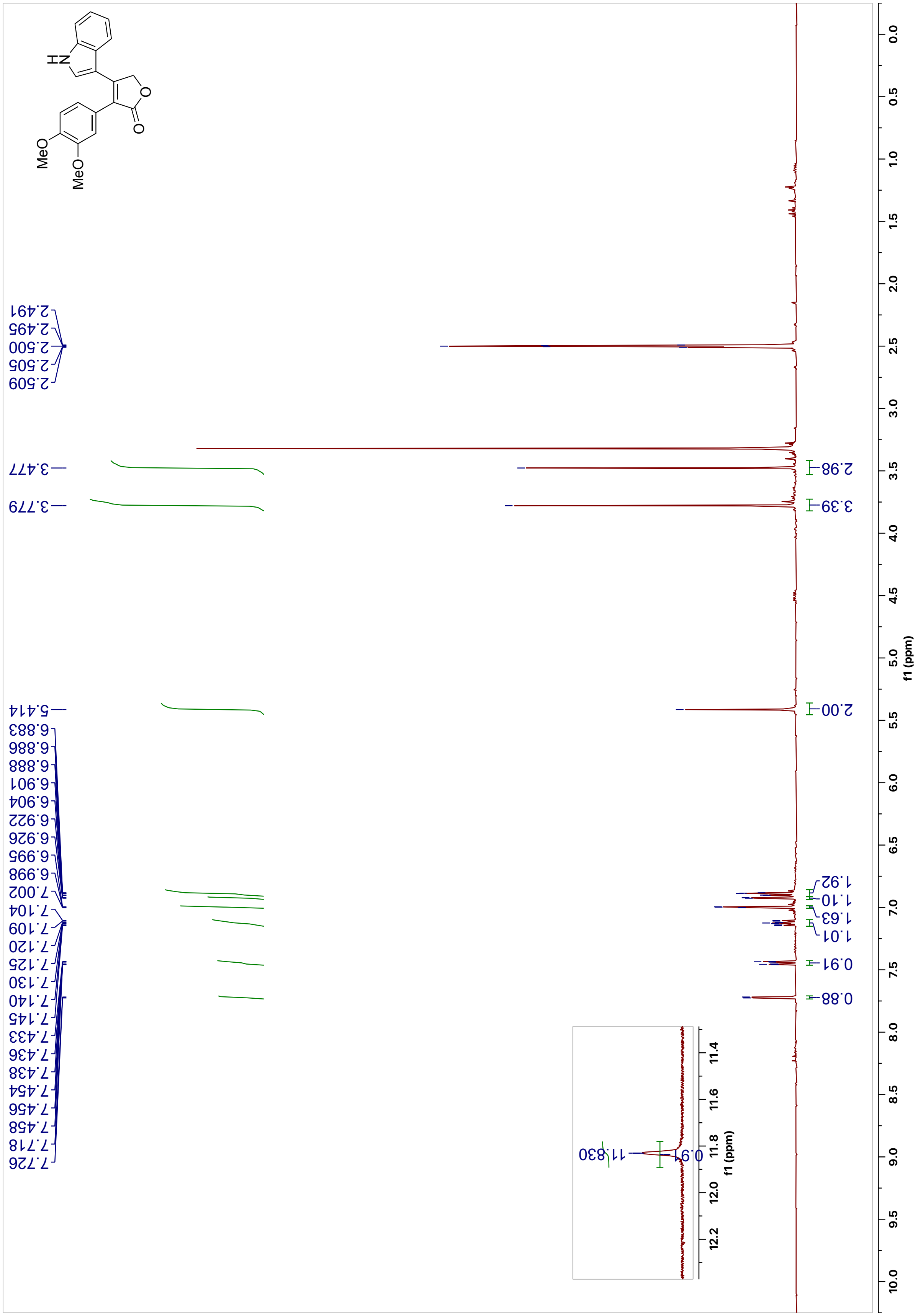

Figure S25b: ^{13}C NMR of 20

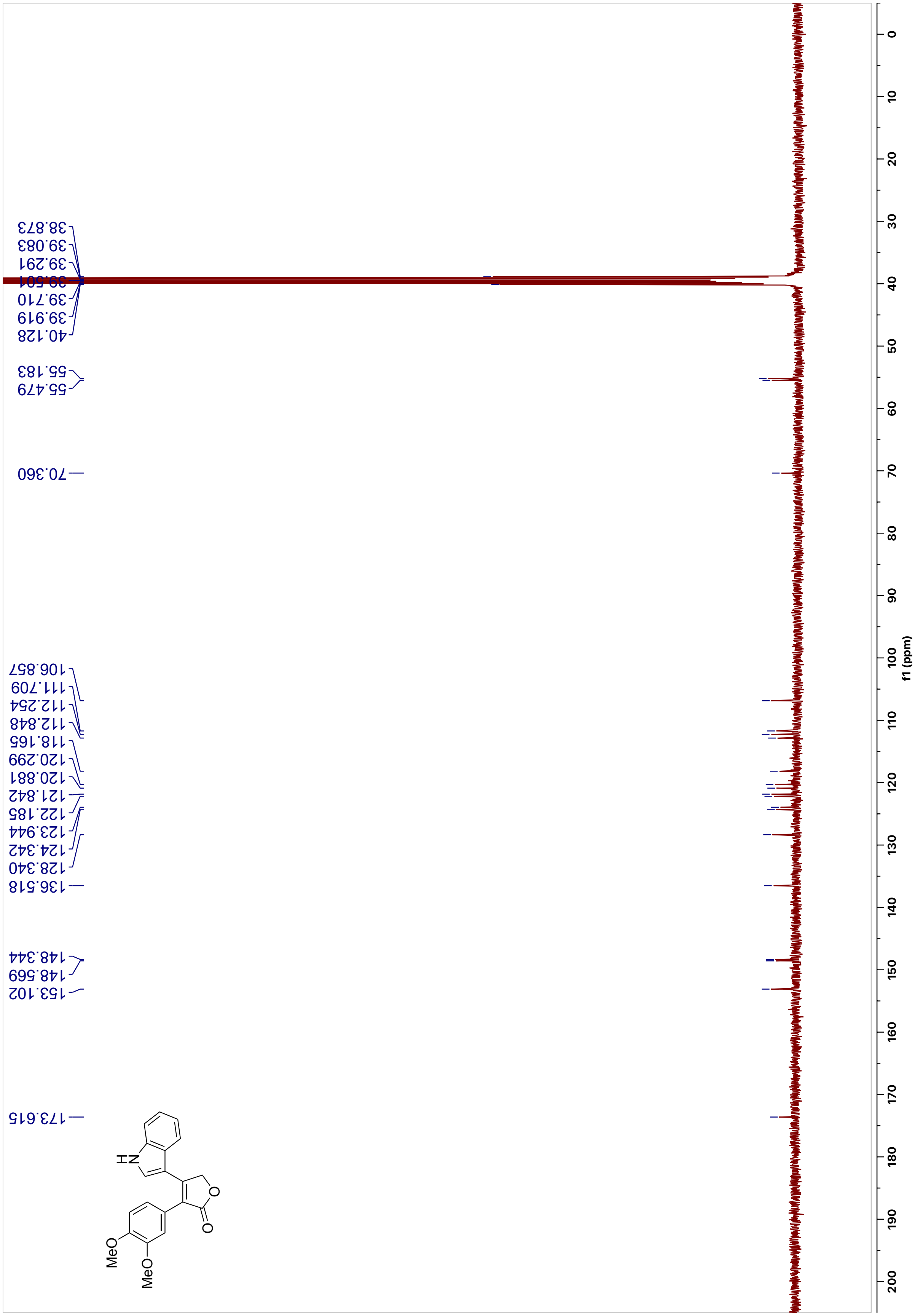

Figure S26a: ^{1}H NMR of 21

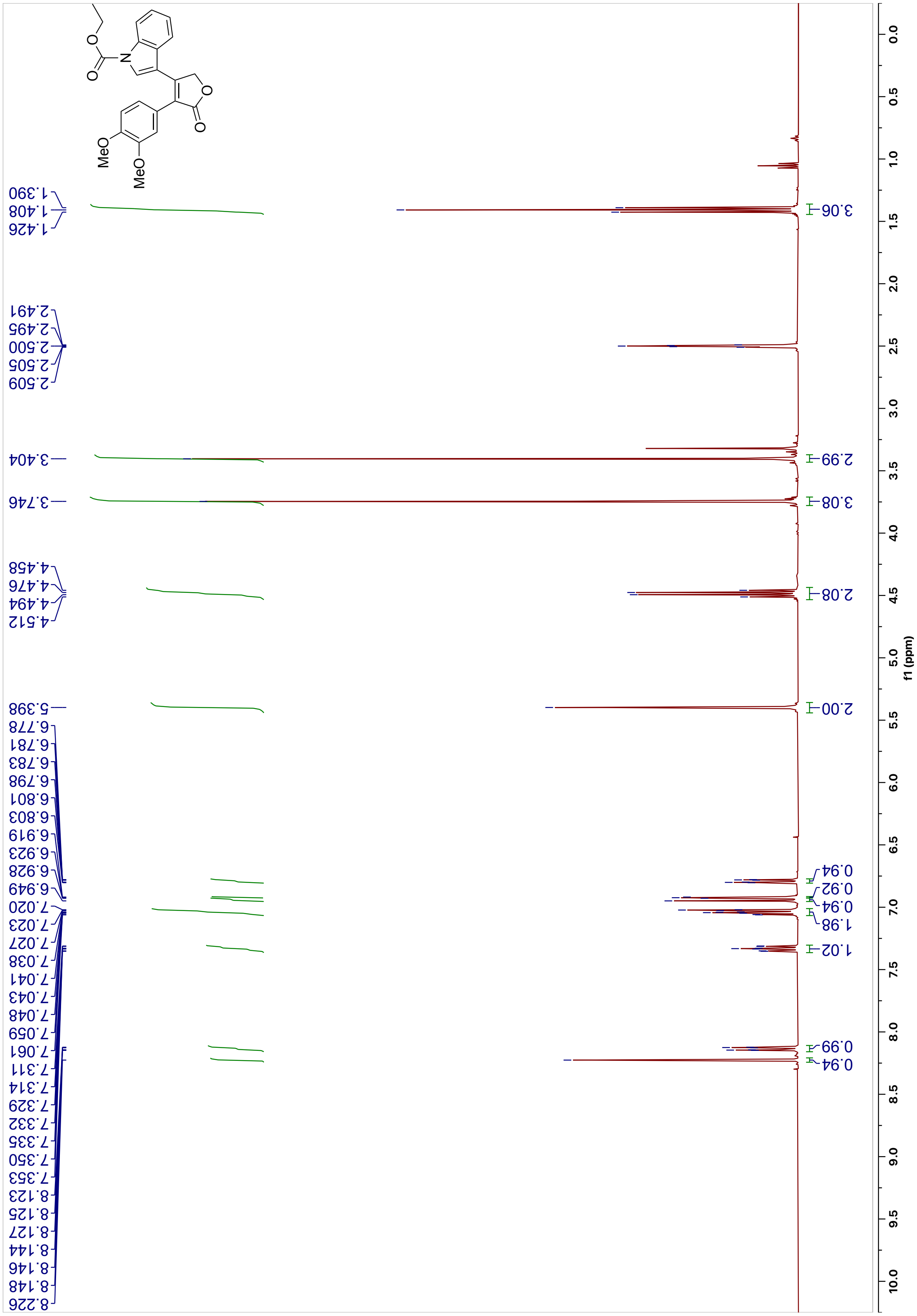

Figure S26b: ^{13}C NMR of 21

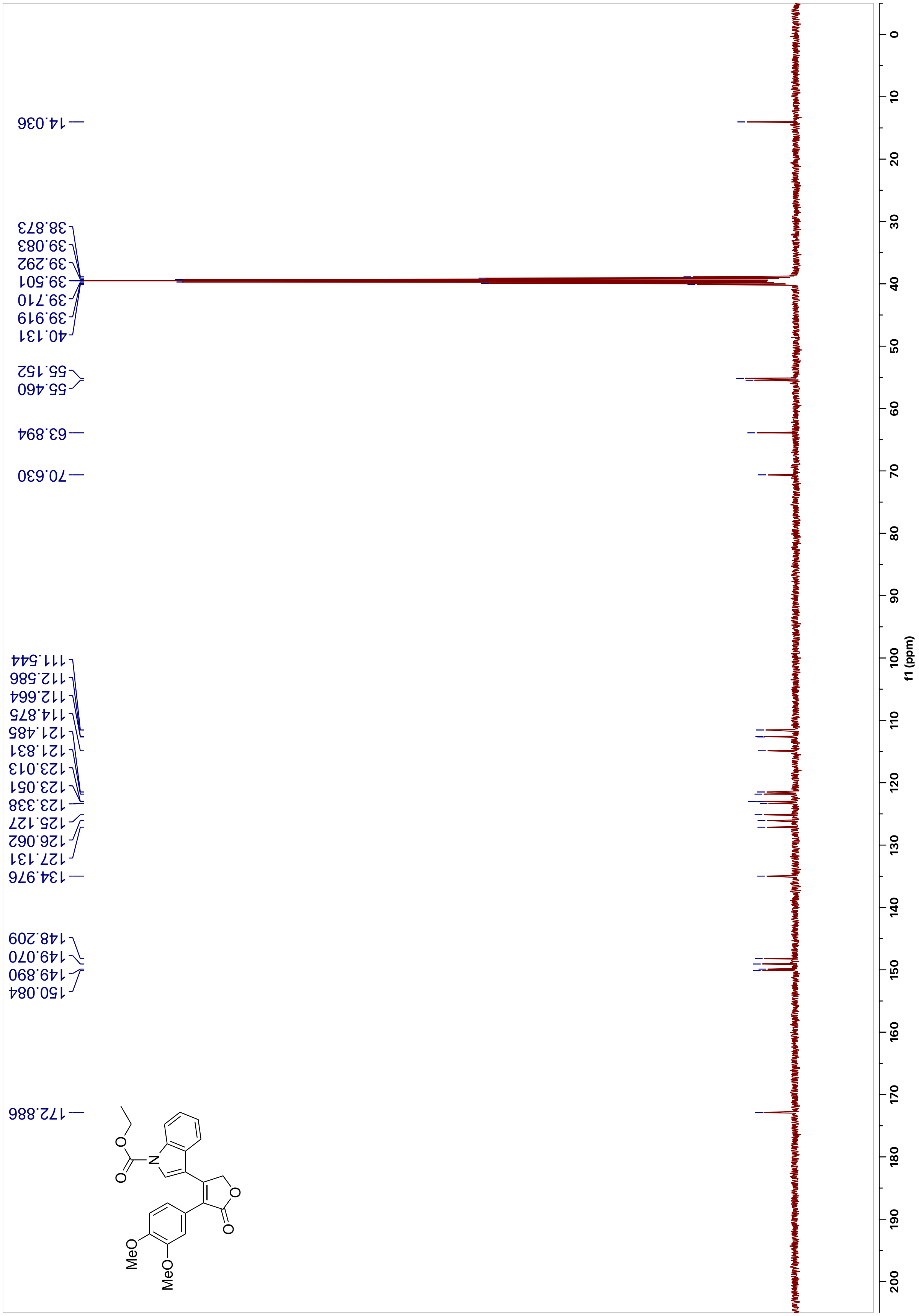

Figure S27a: ^{1}H NMR of 23

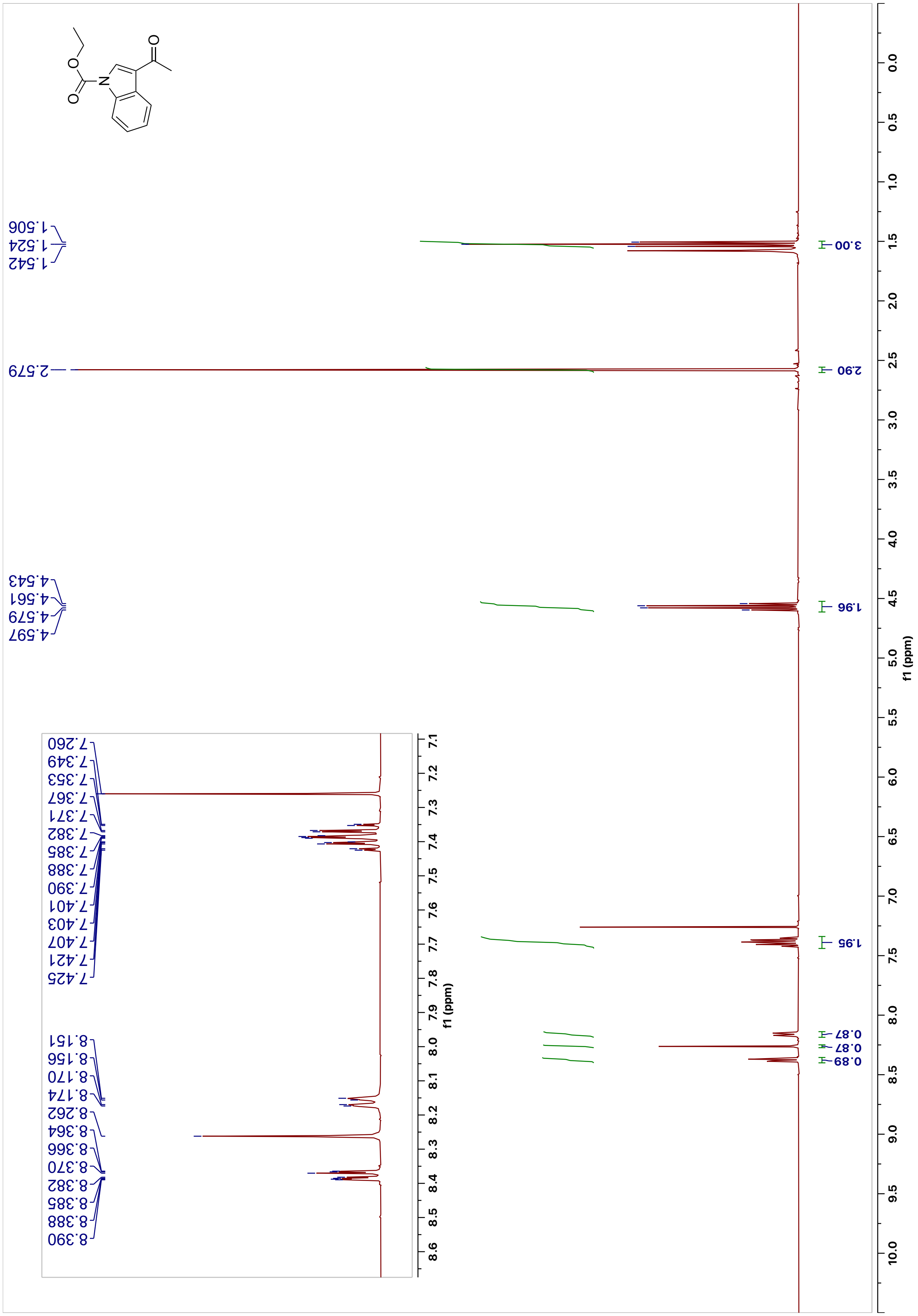

Figure S27b: ^{13}C NMR of 23

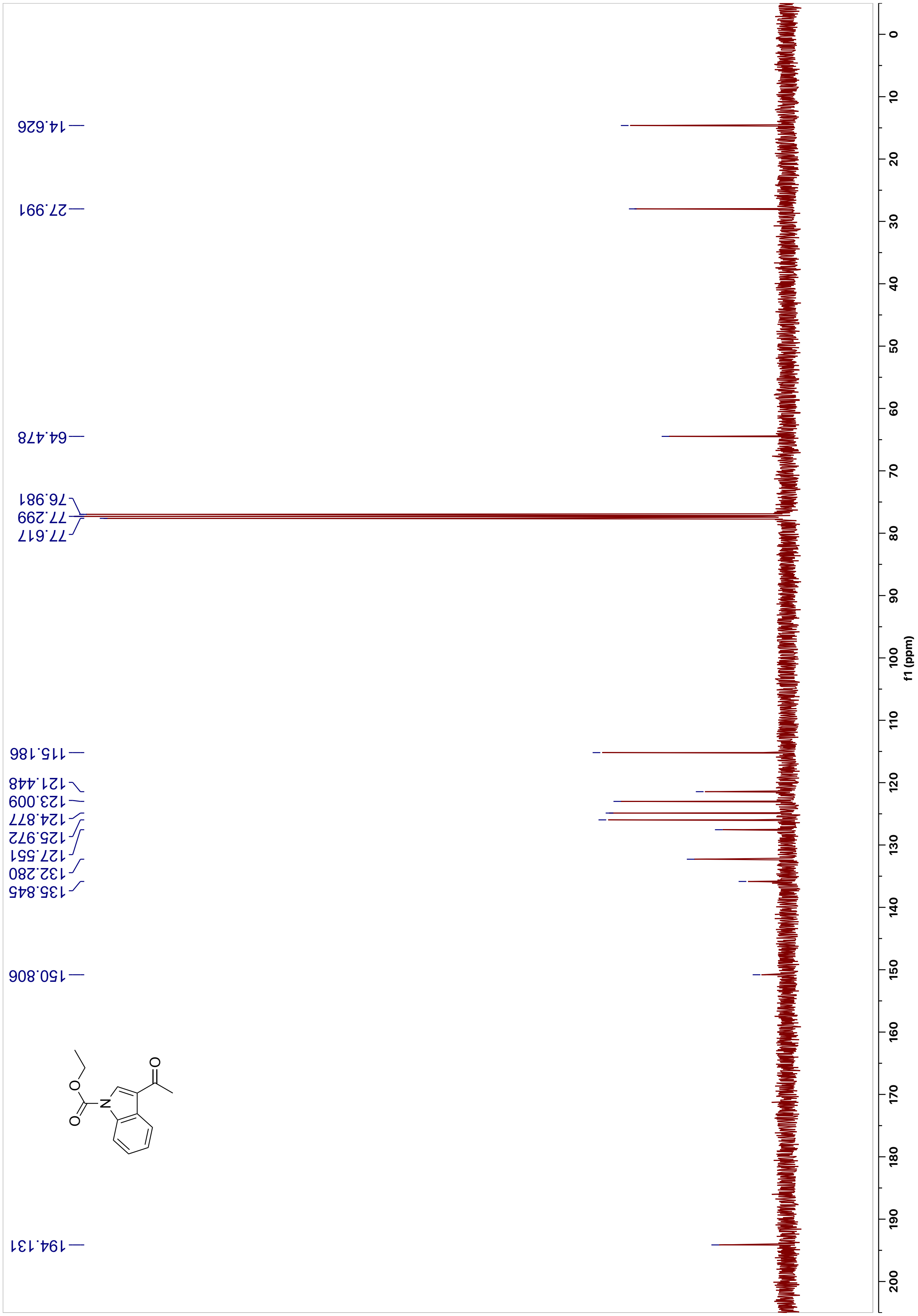

Figure S28a: ^{1}H NMR of 24

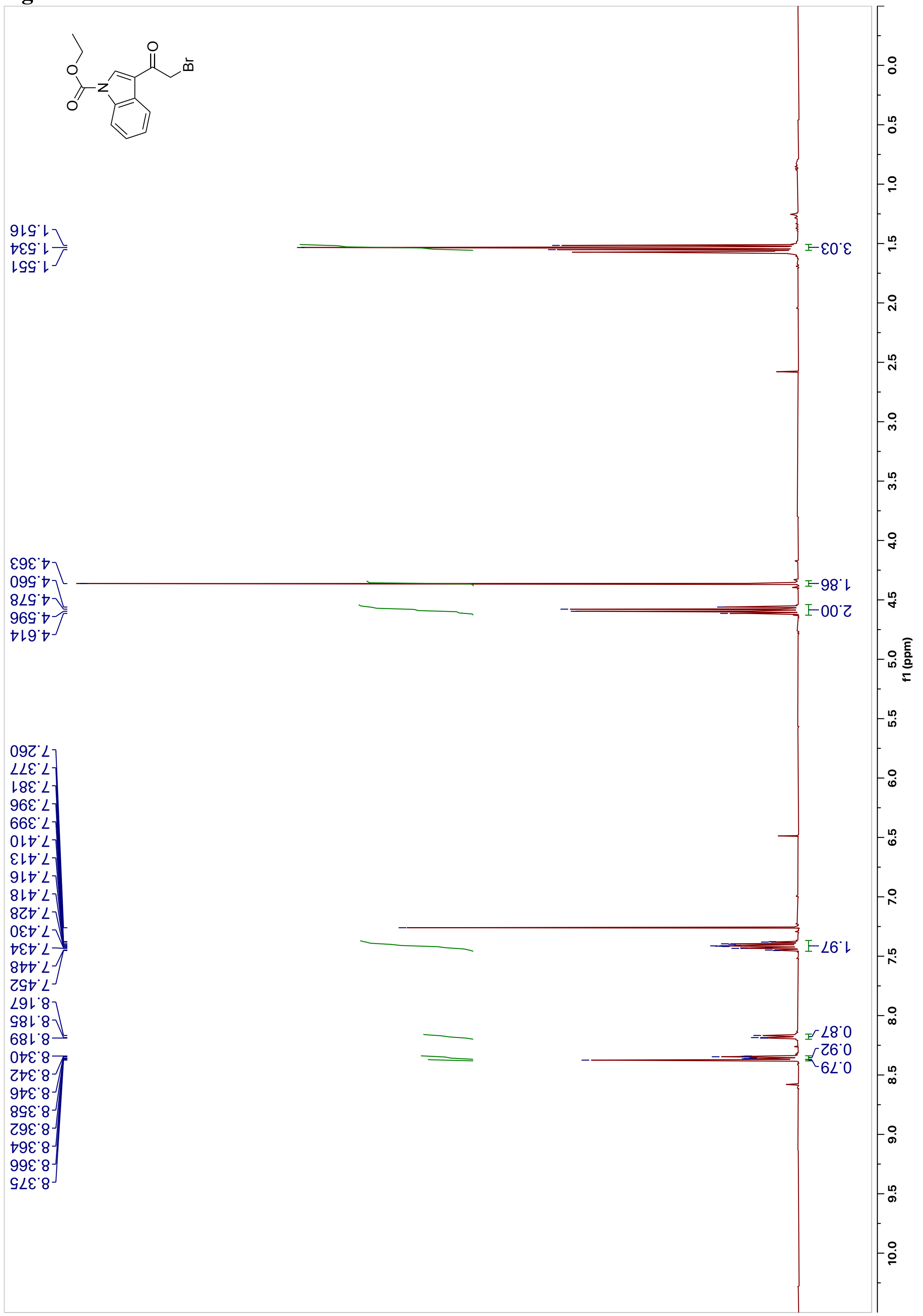

Figure S28b: ^{13}C NMR of 24

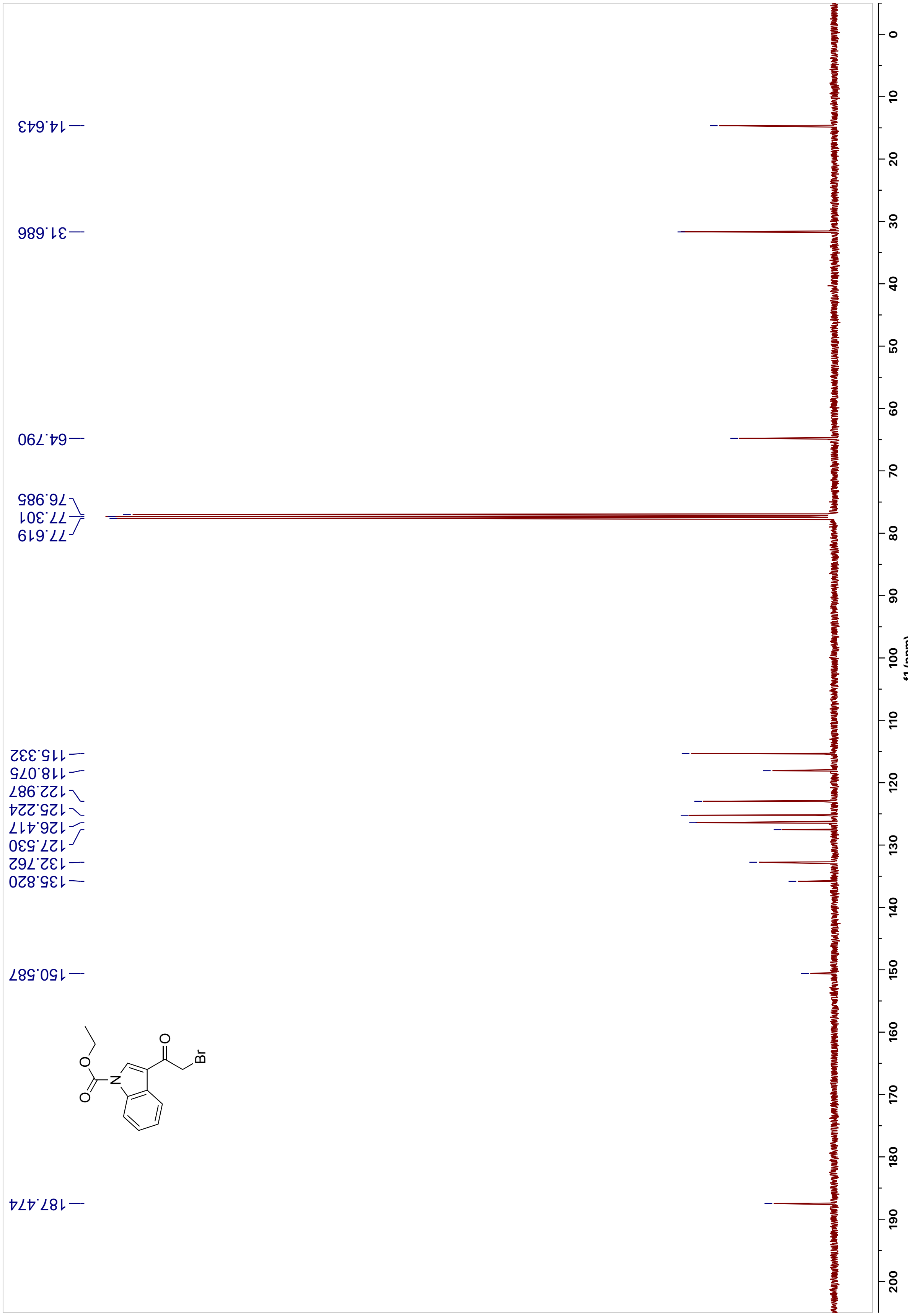

Figure S29a: ^{1}H NMR of 25

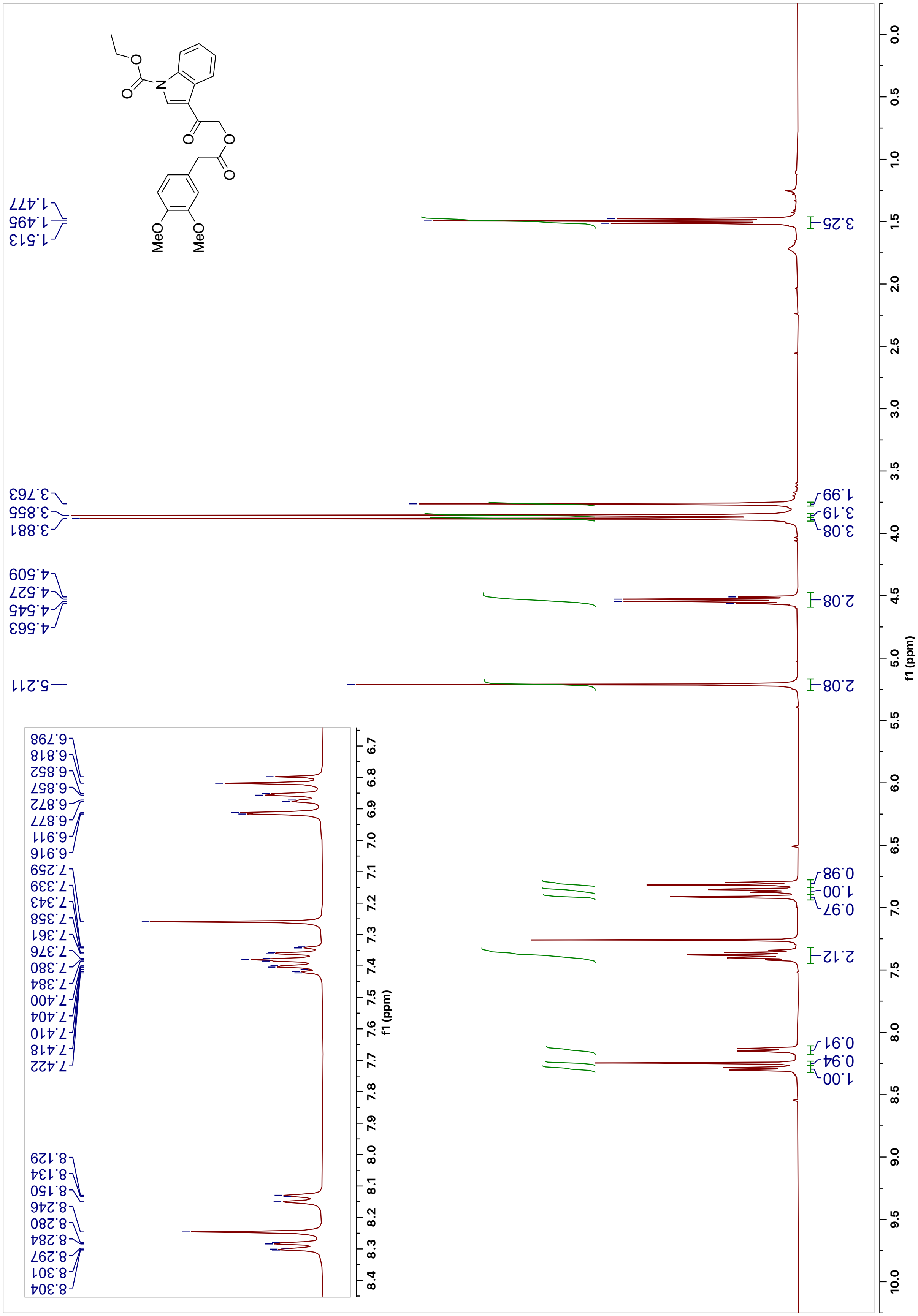

Figure S29b: ^{13}C NMR of 25

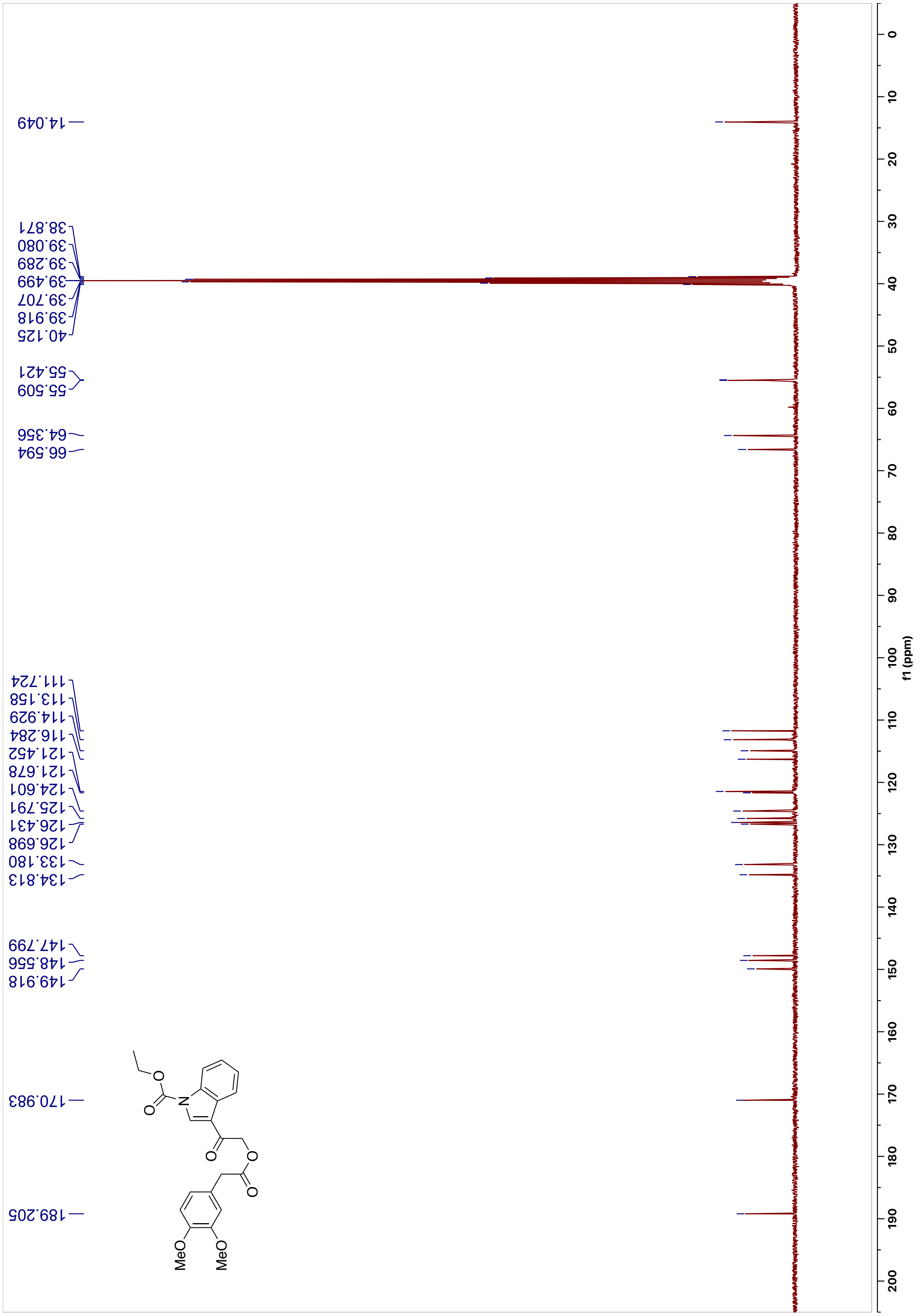

Figure S30a: ^{1}H NMR of 26

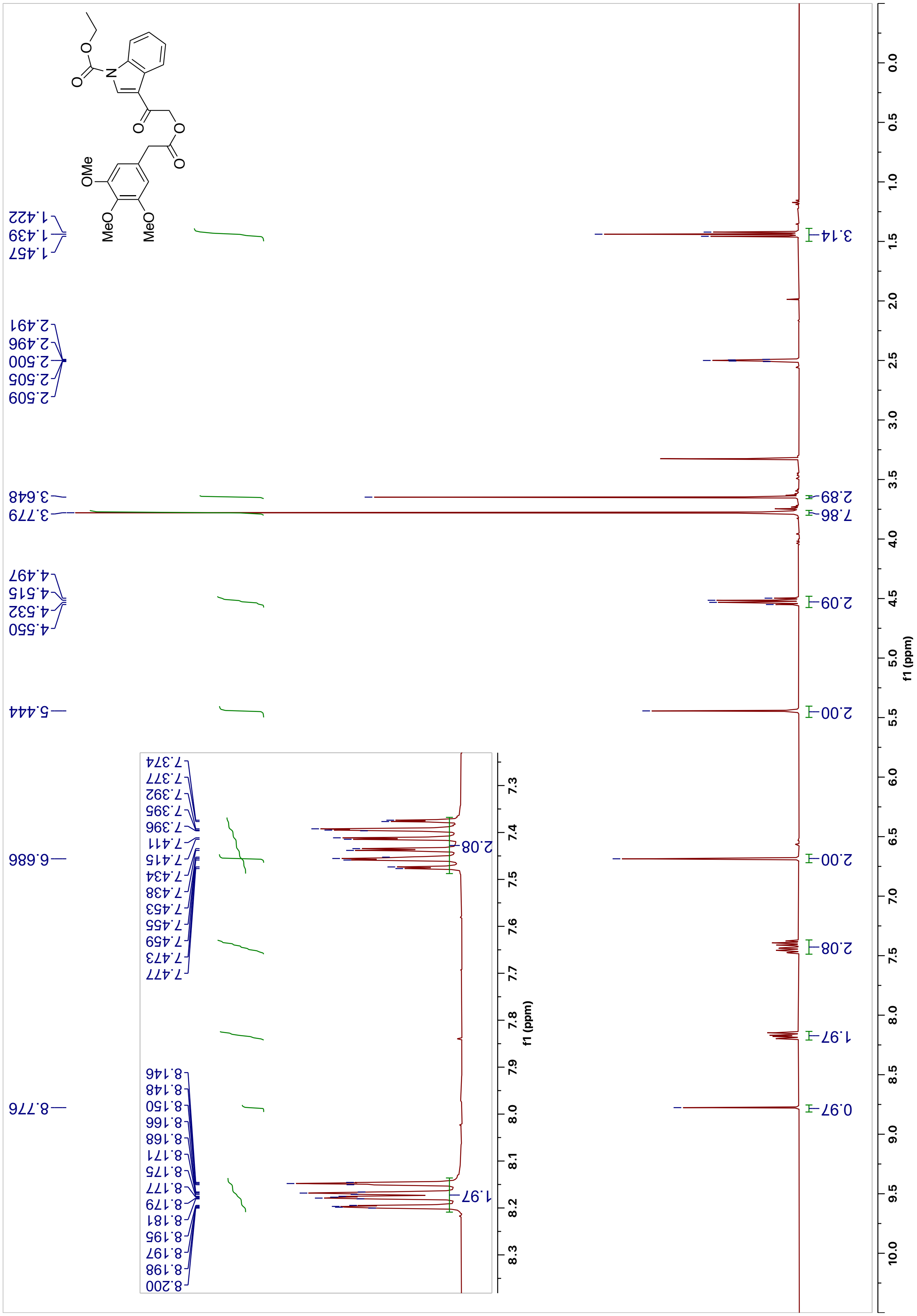

Figure S30b: ^{13}C NMR of 26

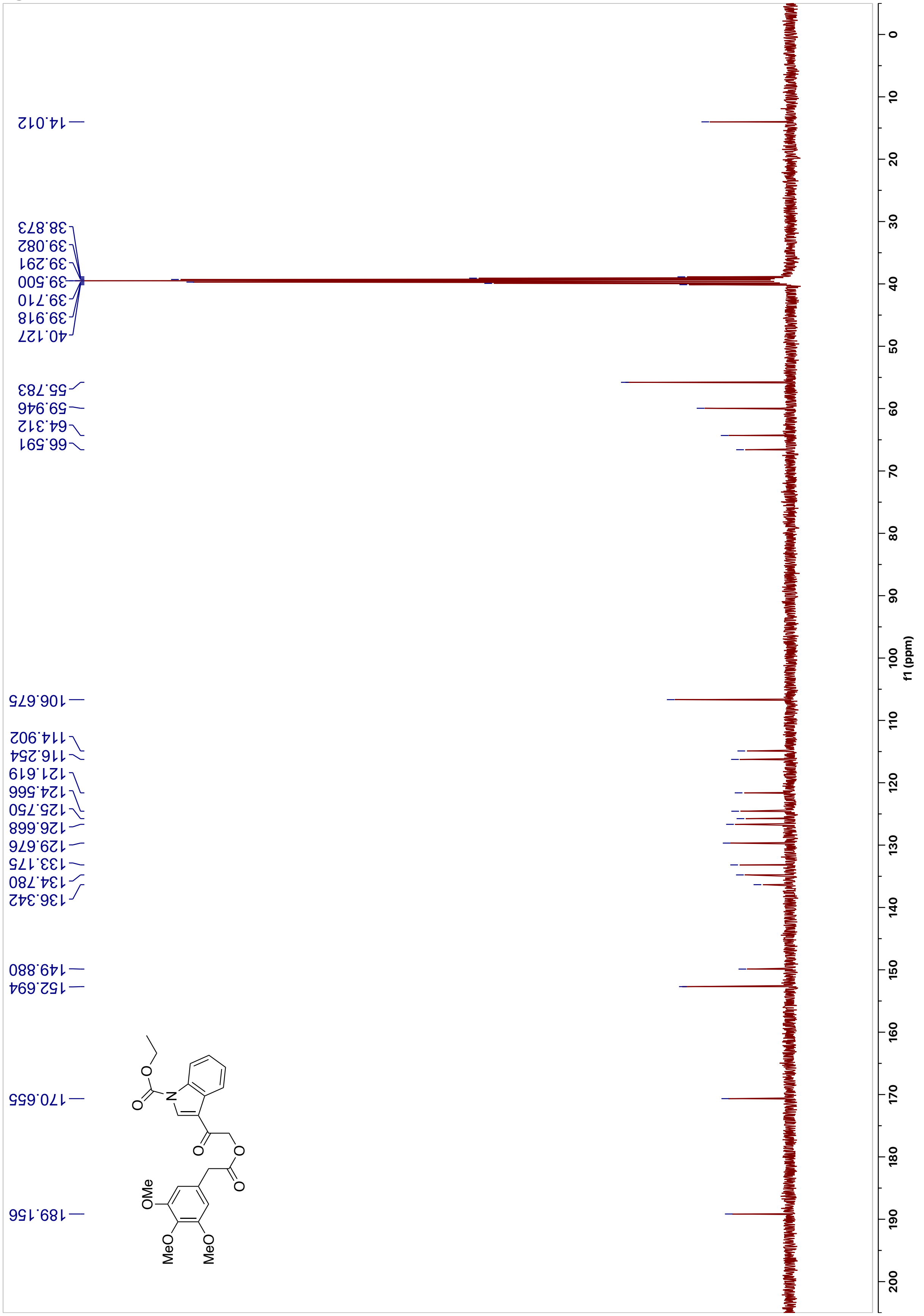

Figure S31a: ^{1}H NMR of 29

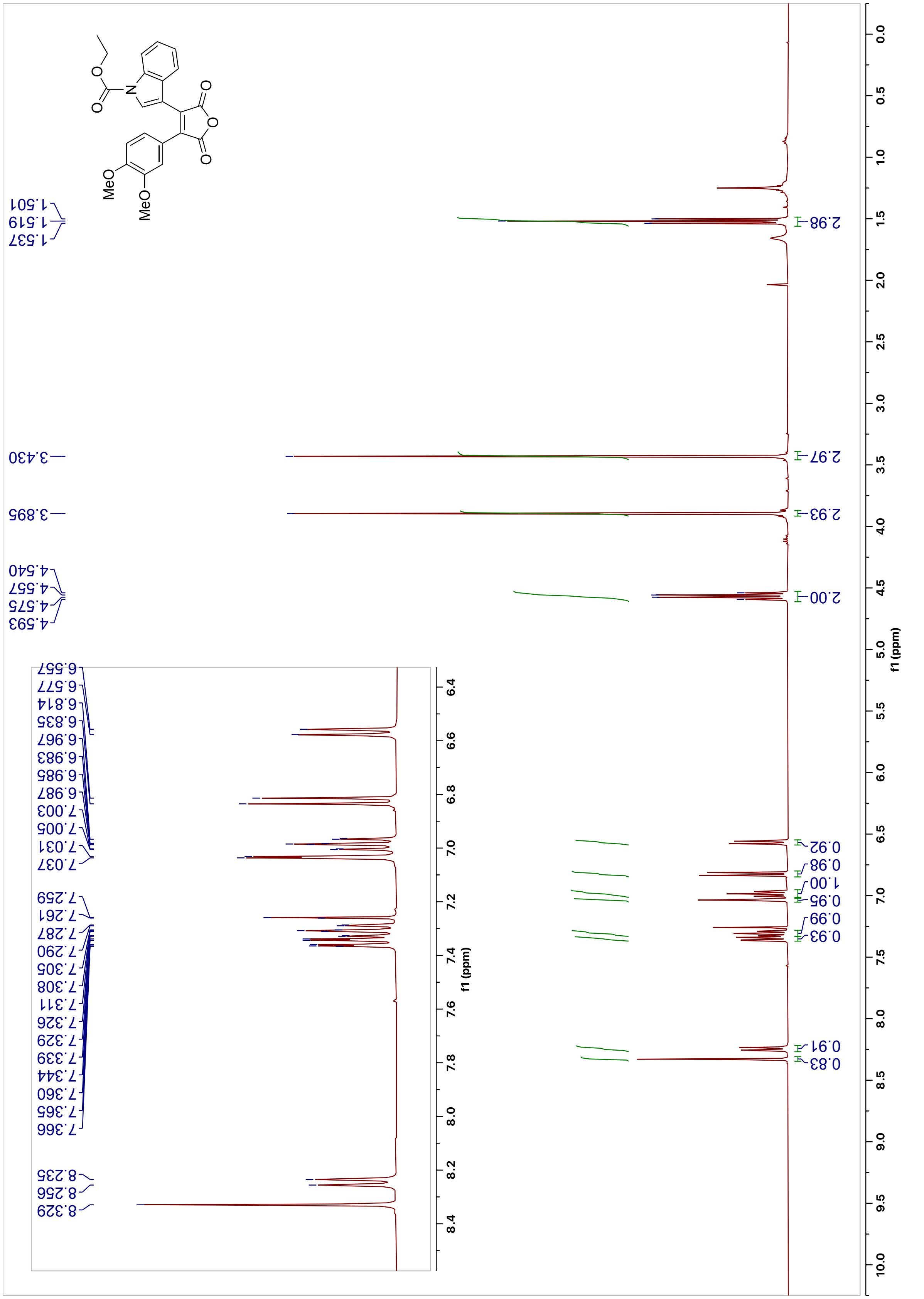

Figure S31b: ^{13}C NMR of 29

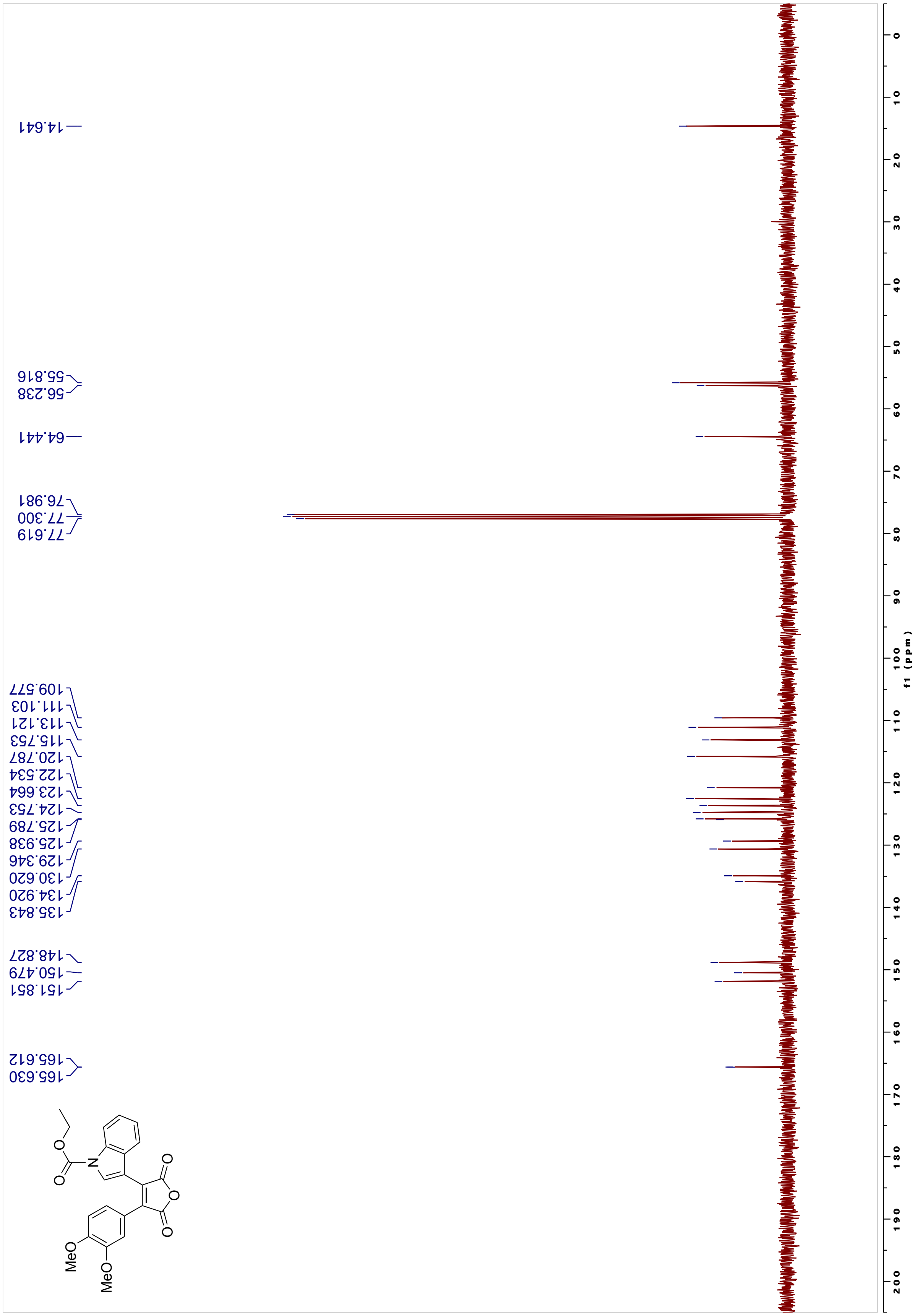

Figure S32a: ^{1}H NMR of 30

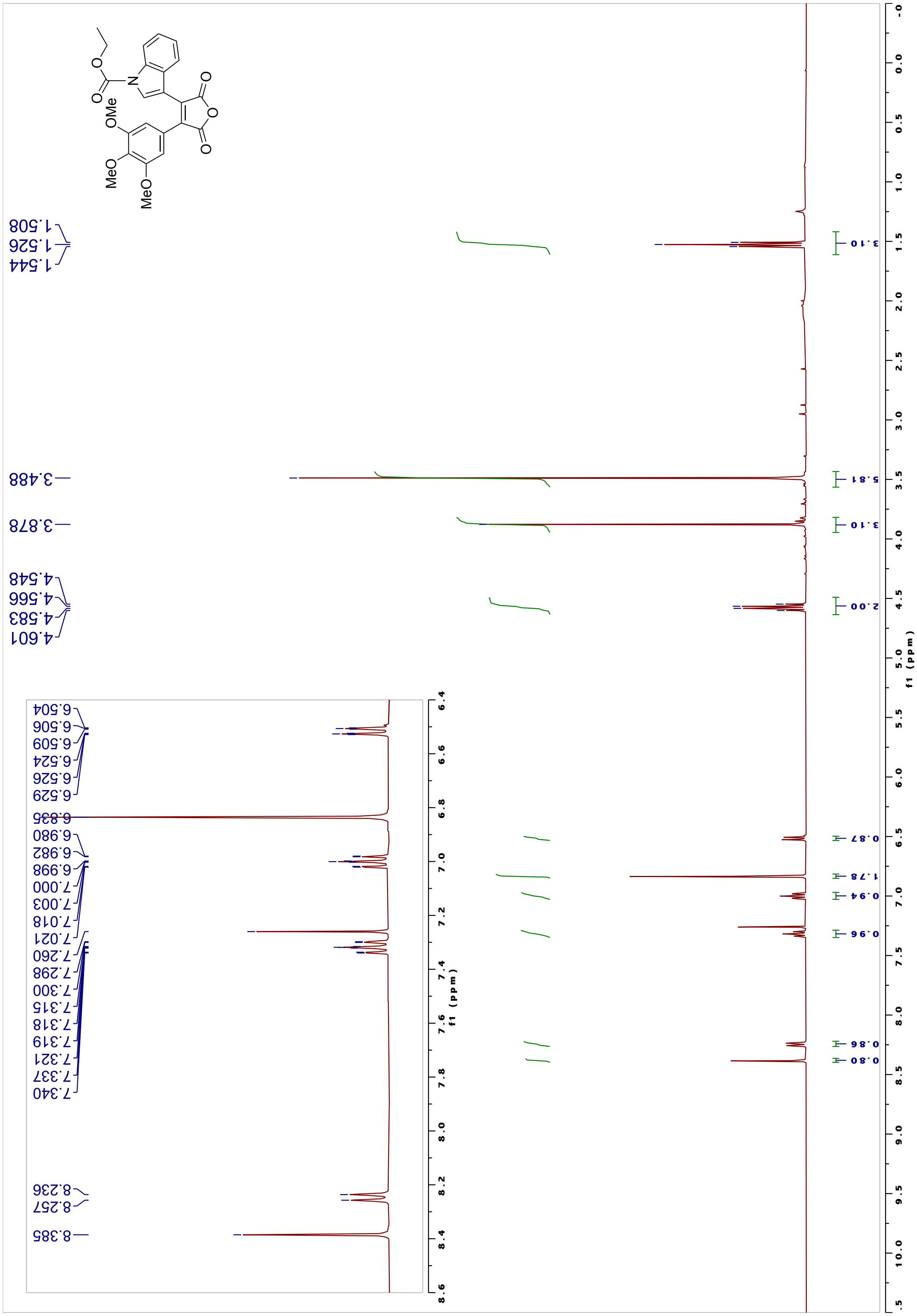

Figure S32b: ^{13}C NMR of 30

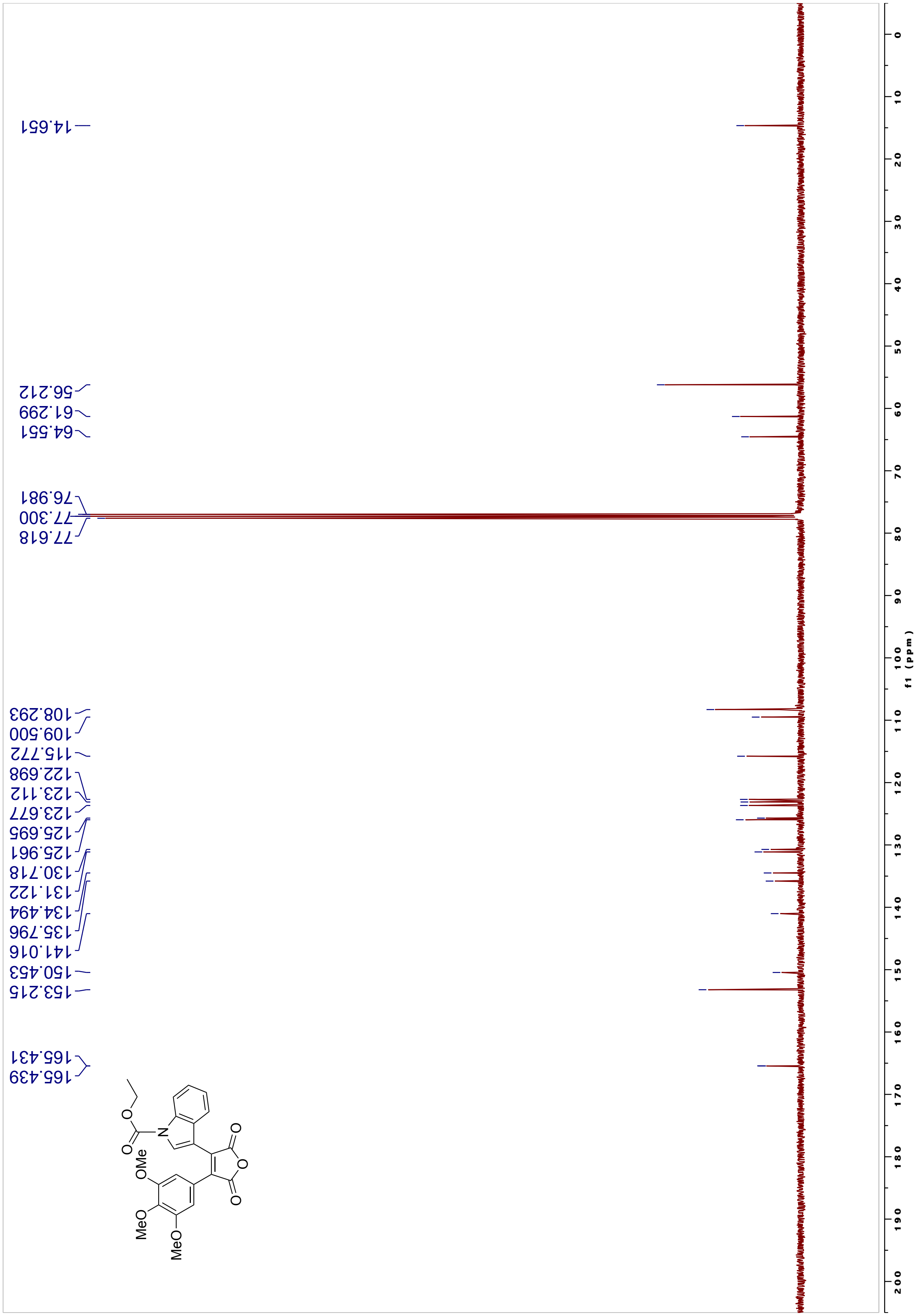

Figure S33a: ^{1}H NMR of 31

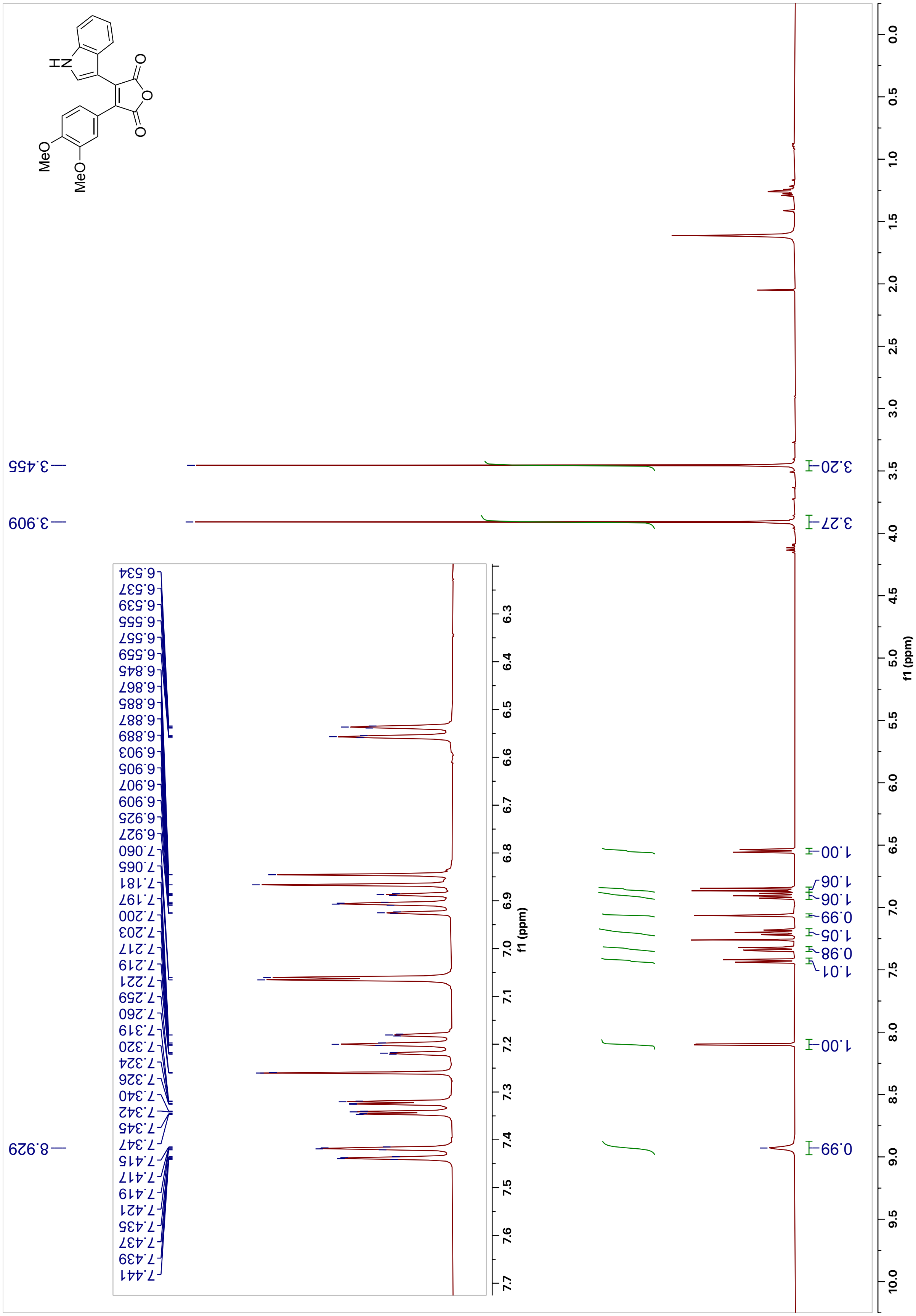

Figure S33b: ^{13}C NMR of 31

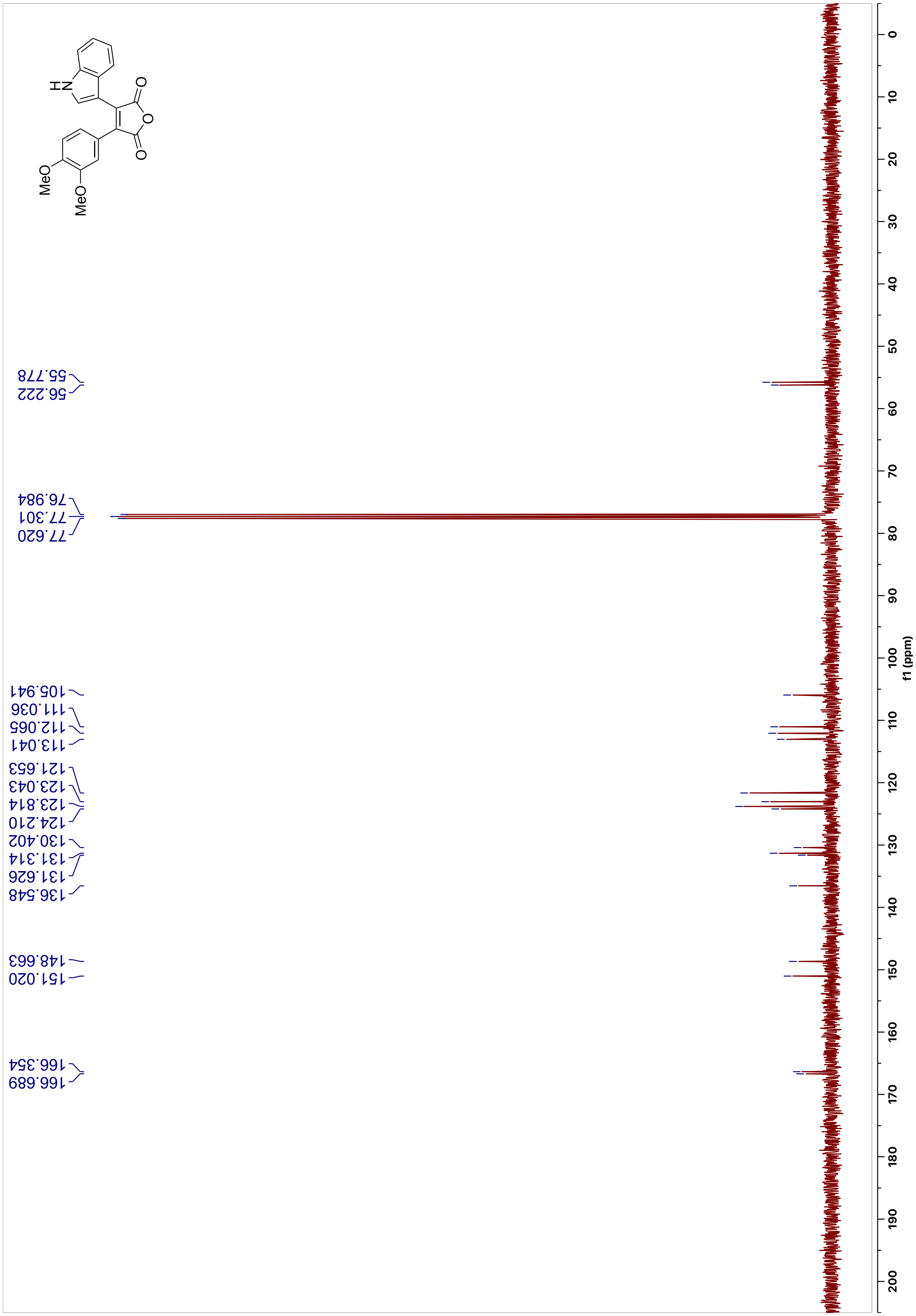

Figure S34a: ^{1}H NMR of 32

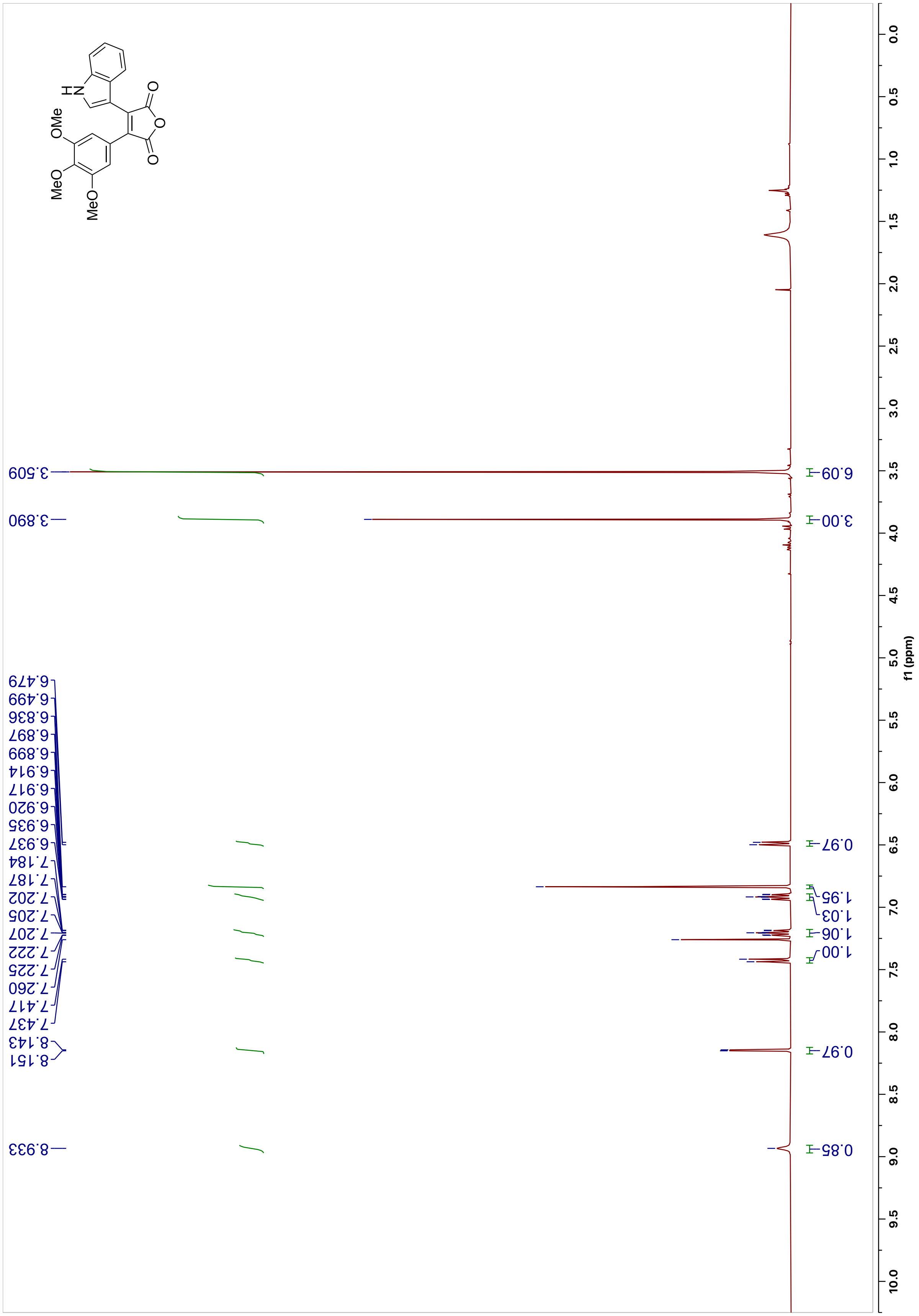

Figure S34b: ^{13}C NMR of 32

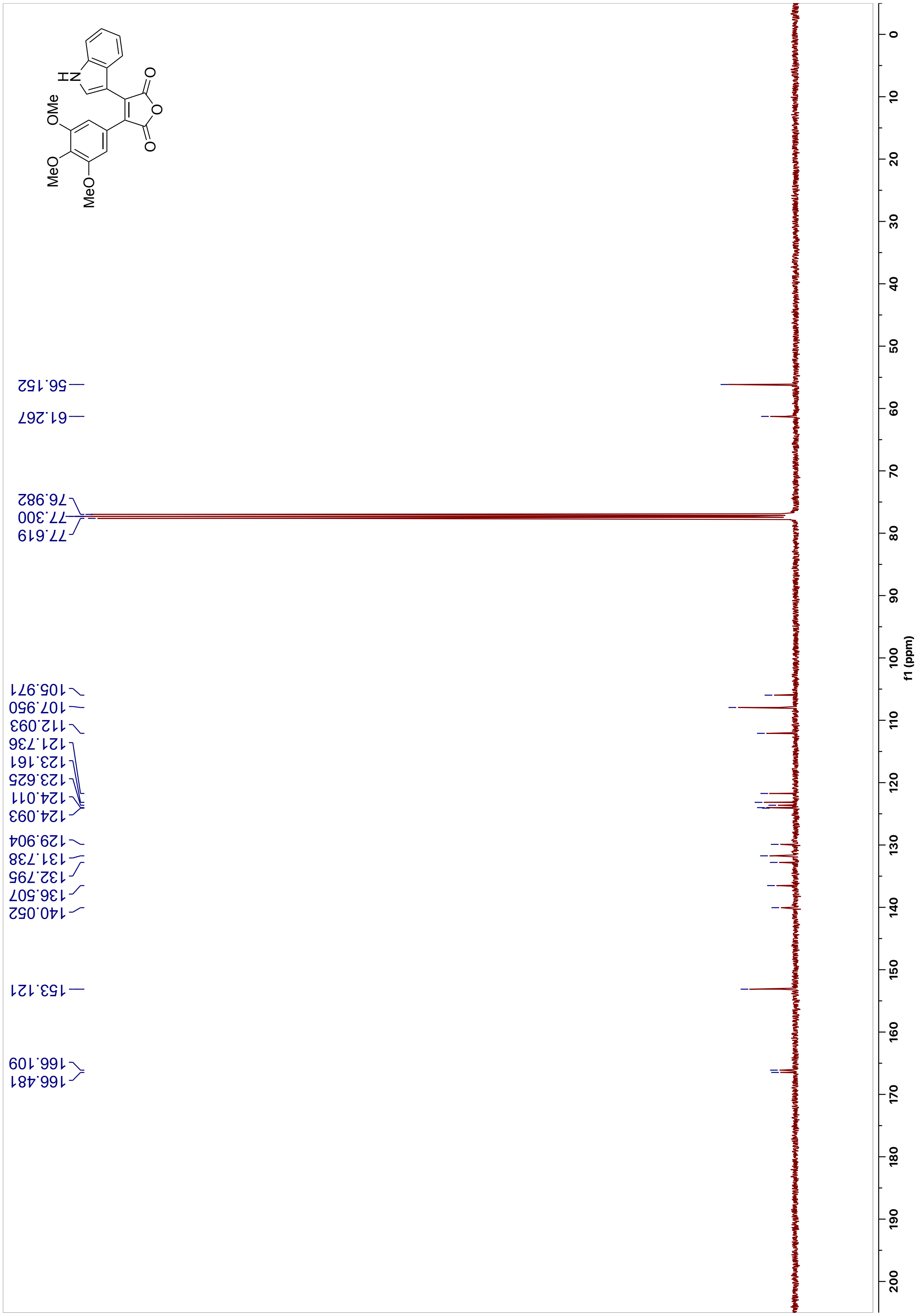

Figure S35a: ^{1}H NMR of 33

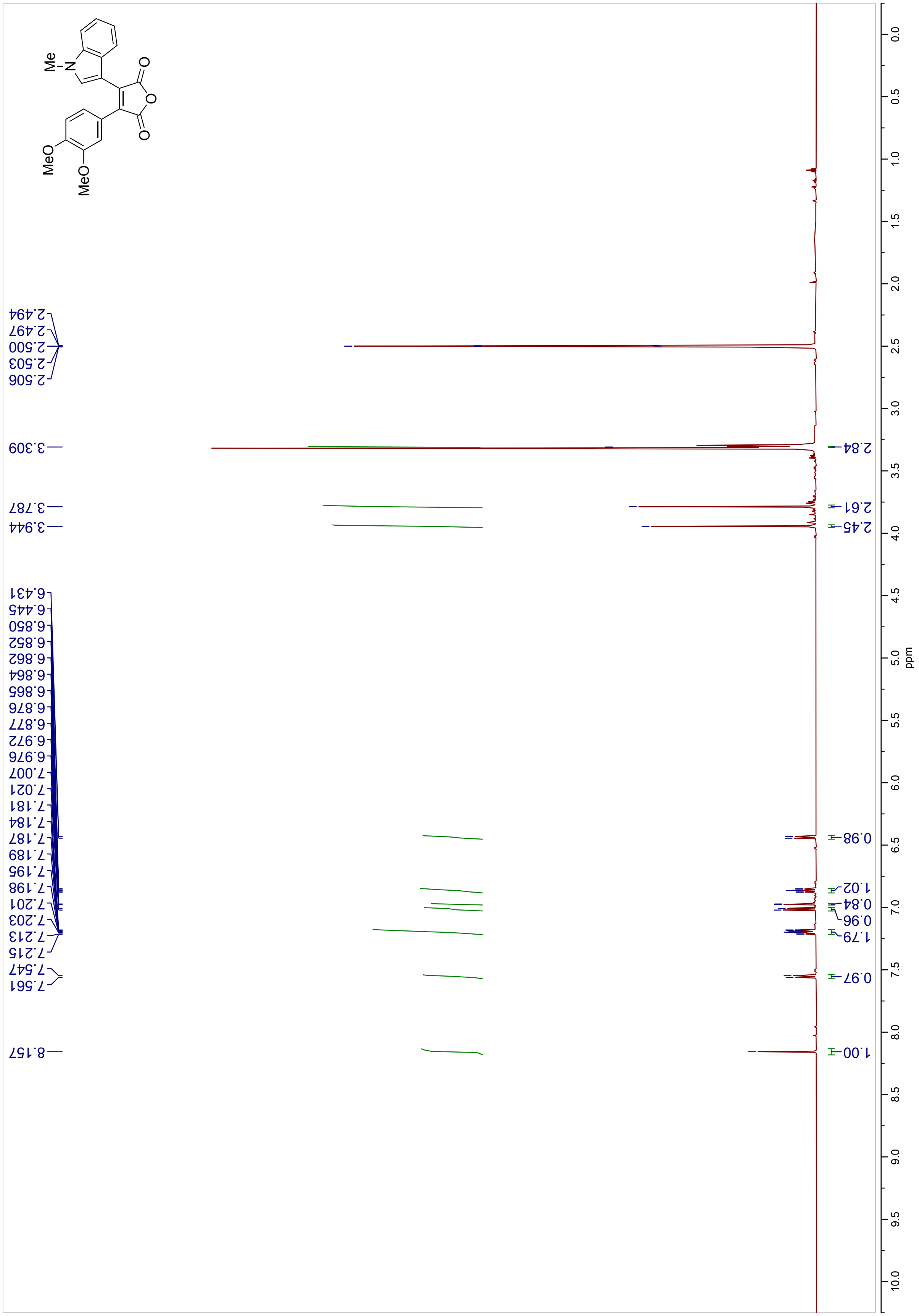

Figure S35b: ^{13}C NMR of 33

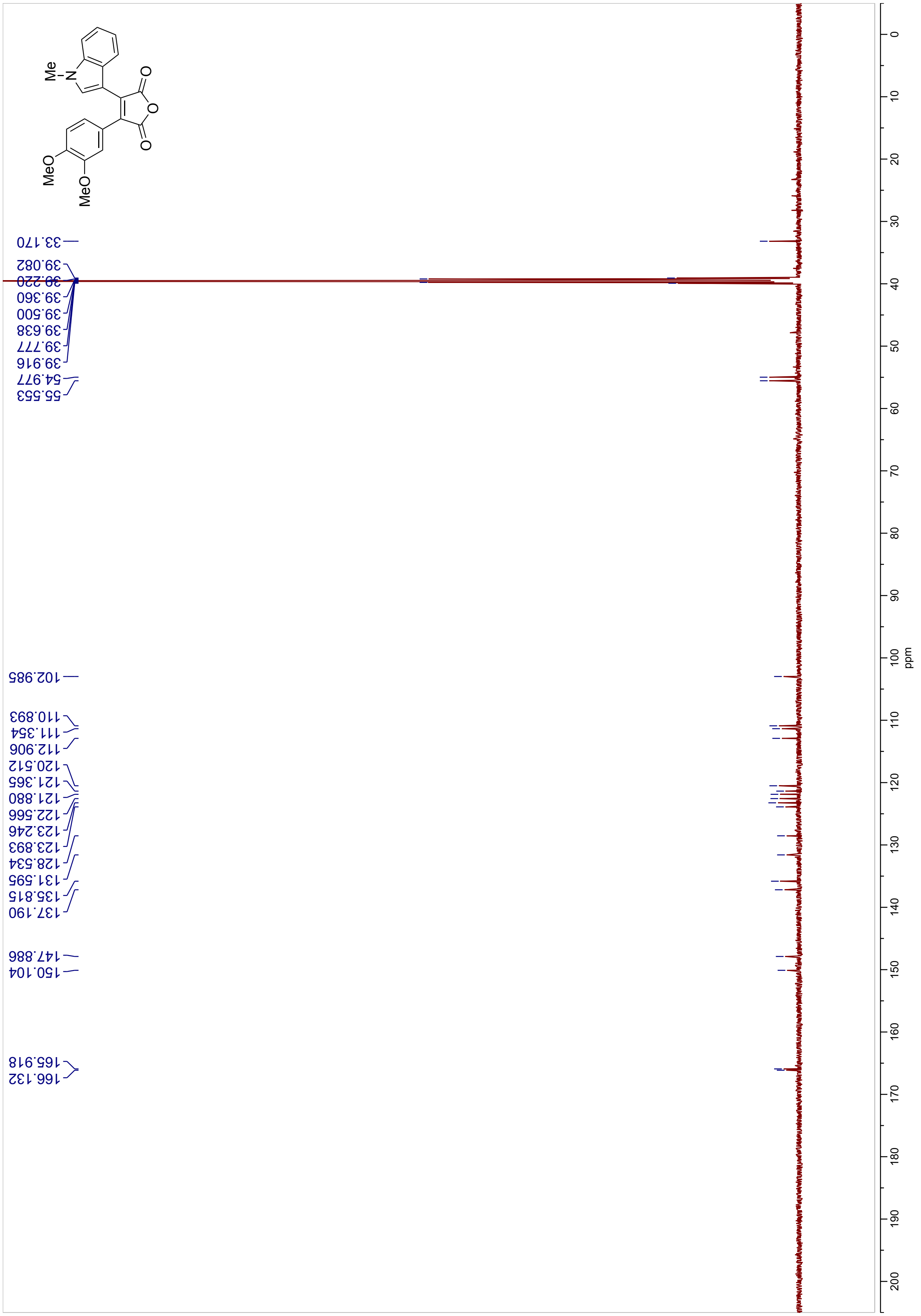

Figure S36a: ^{1}H NMR of 34

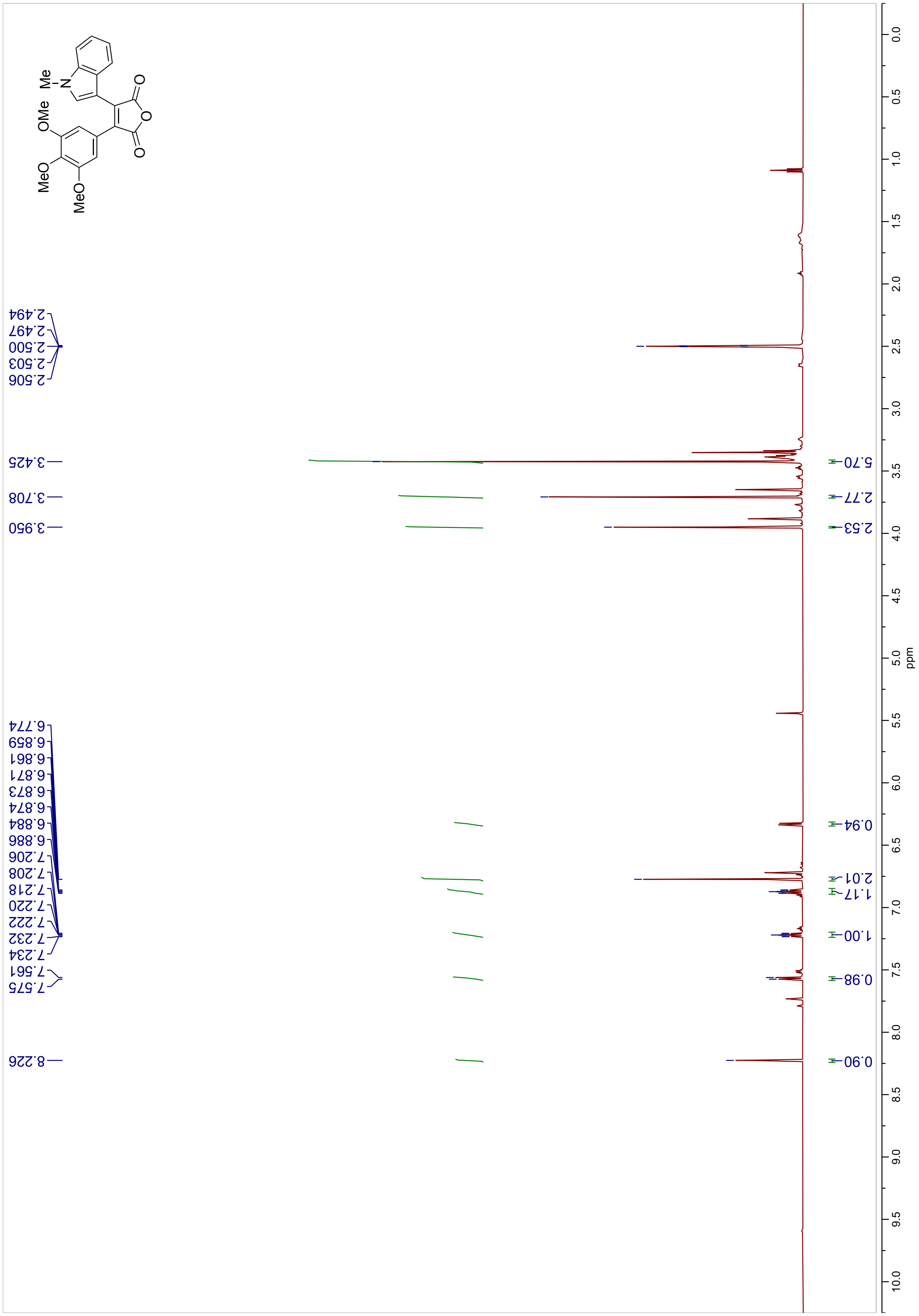

Figure S36b: ^{13}C NMR of 34

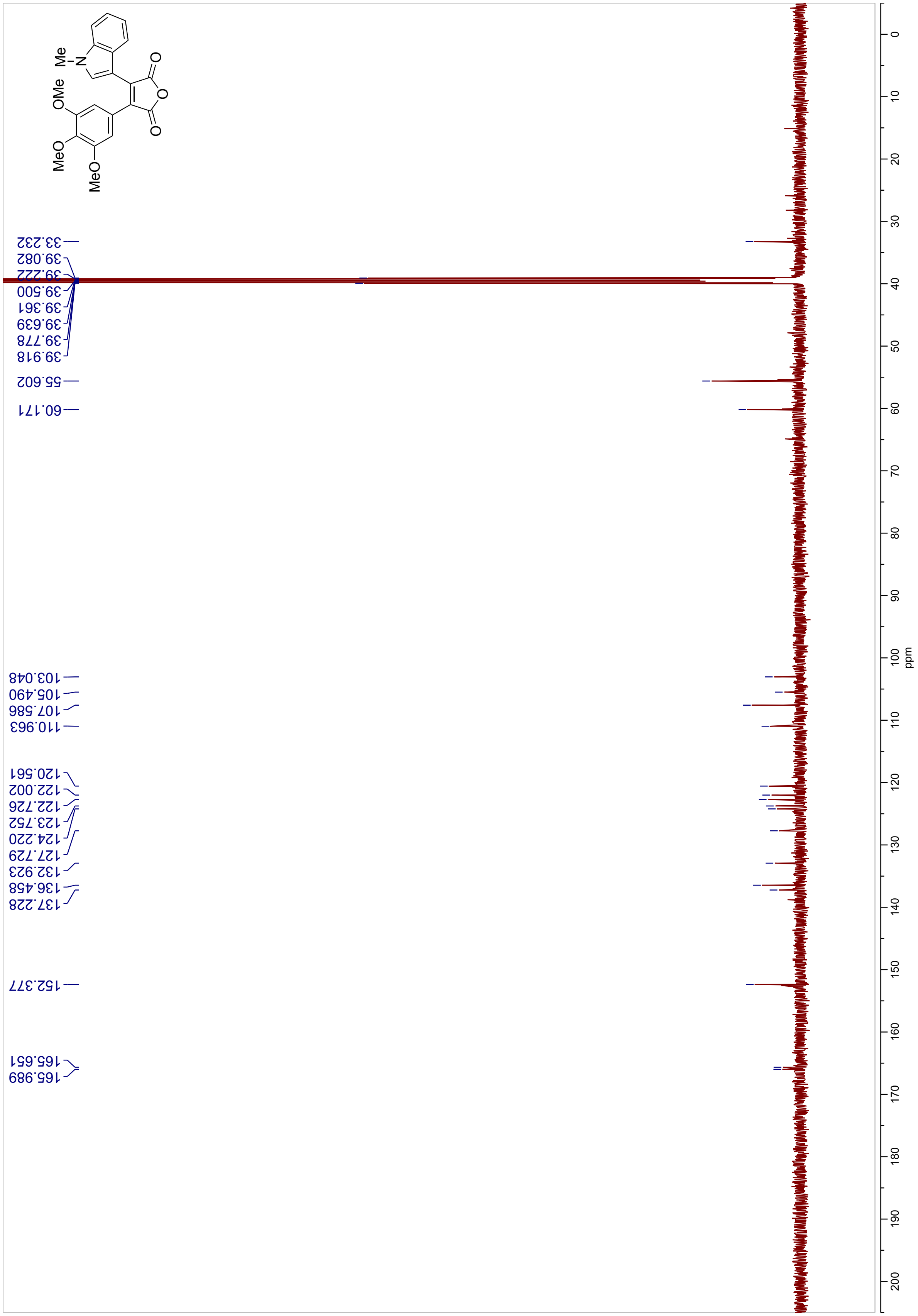

Figure S37a: ^{1}H NMR of 35

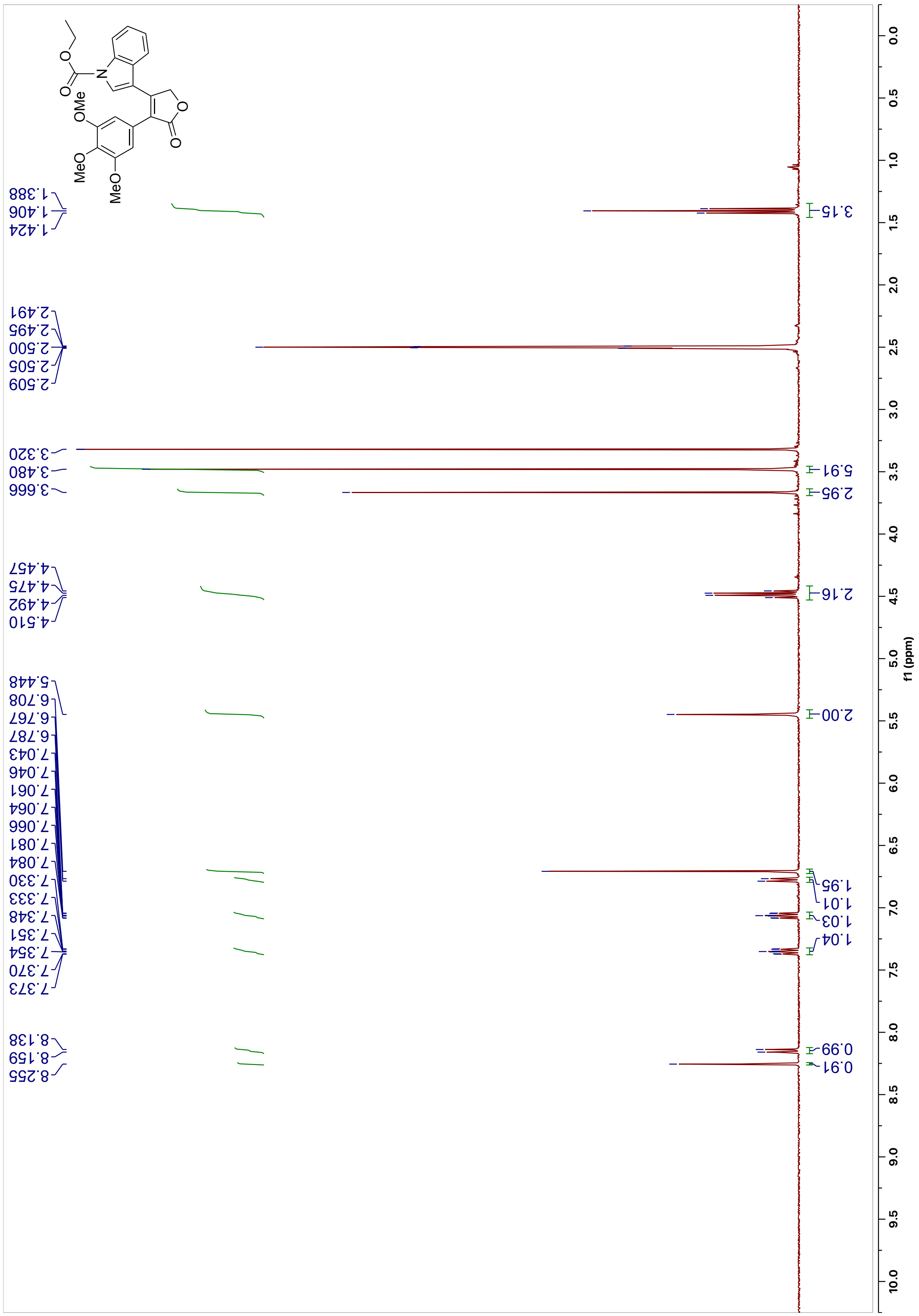

Figure S37b: ^{13}C NMR of 35

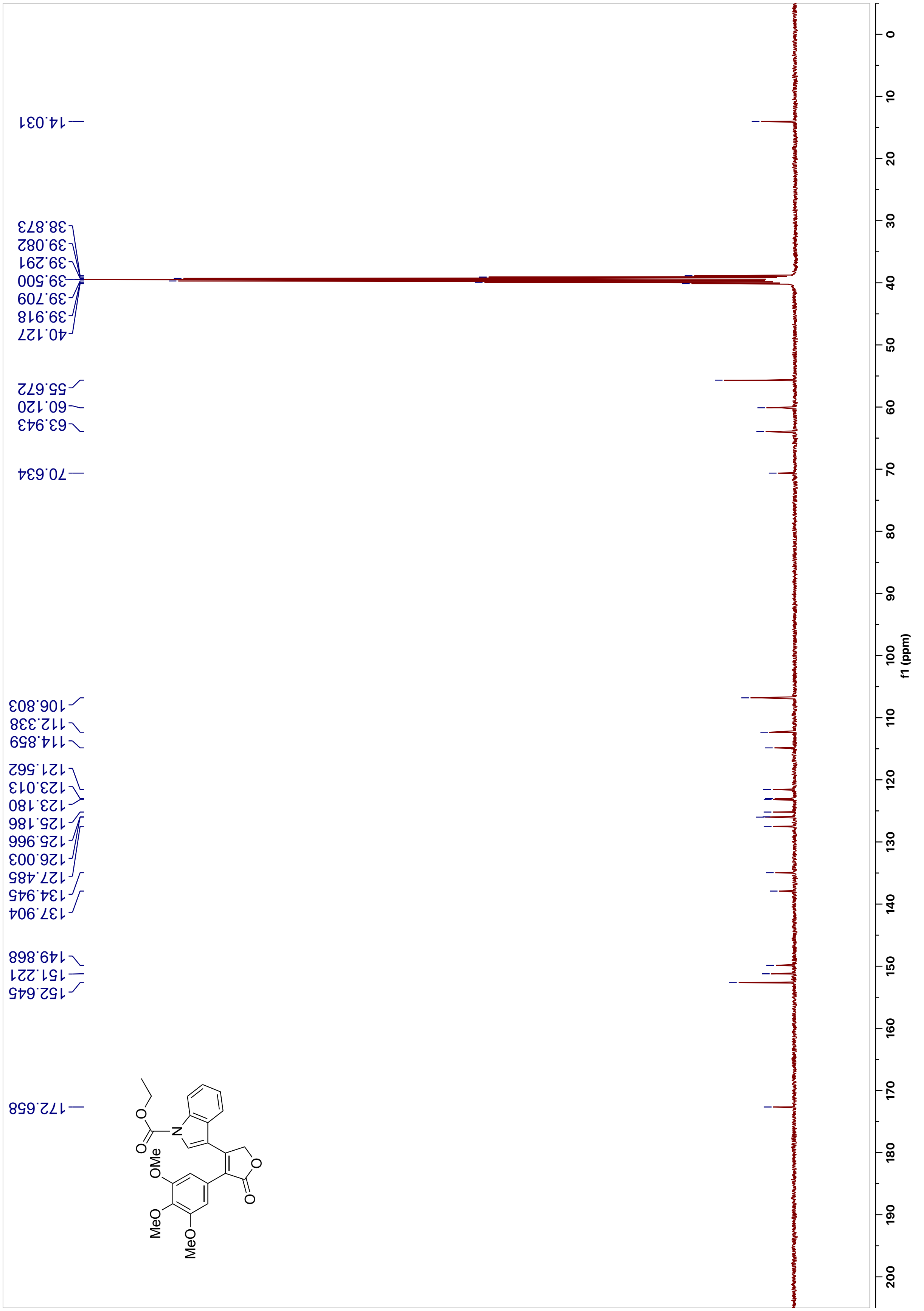

Figure S38a: ^{1}H NMR of 36

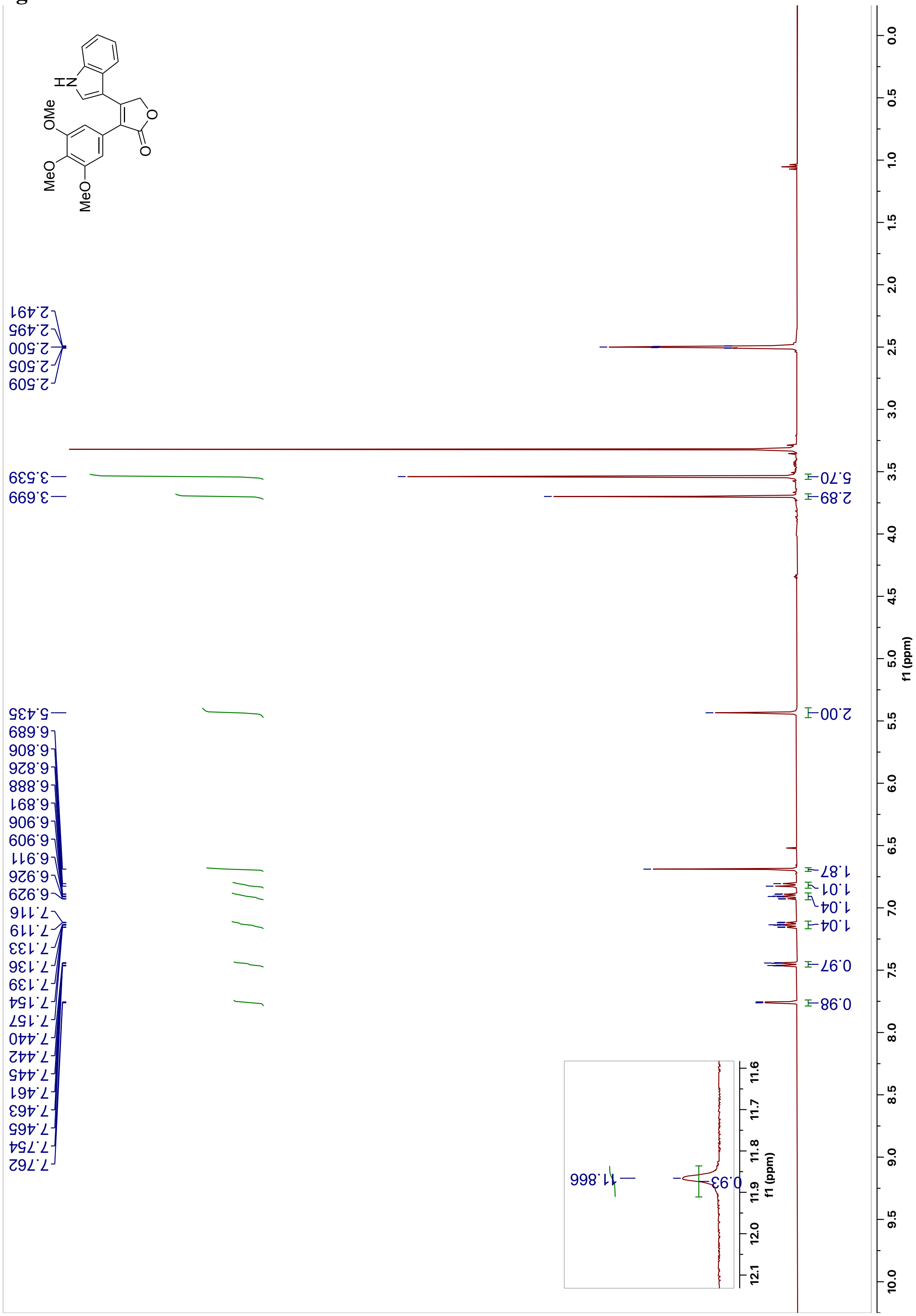

Figure S38b: ^{13}C NMR of 36

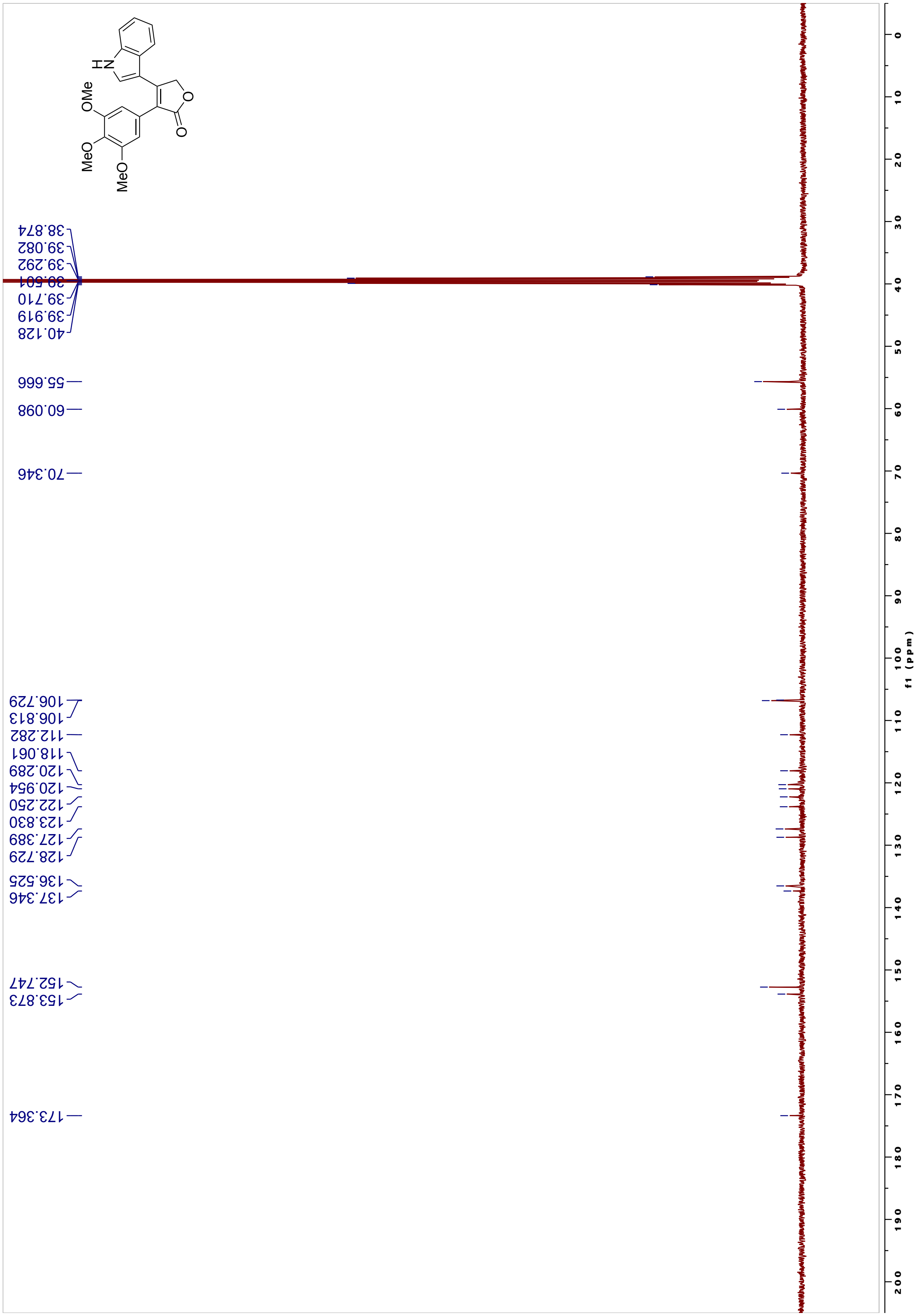

Supplement: Supplementary file 1 [file ao5c07360_si_001.pdf]
